# Supplementary material for: IL6-mediated HCoV-host interactome regulatory network and GO/Pathway enrichment analysis
Source: PLoS Comput Biol. 2020 Sep 30;16(9):e1008238. doi: 10.1371/journal.pcbi.1008238 (PMC7561109; doi:10.1371/journal.pcbi.1008238)
Supplement: S1 Data — Available also as an interactive website at https://precious.polito.it/covid-19/. (PDF) [file pcbi.1008238.s004.pdf]

GO CLASSIFICATION: 2
1 HCOV-HOST_SET 2
1.1 GO CLASSIFICATION 2
1.2 TABLES CLASSIFICATION 4
1.2.1 MOLECULAR FUNCTION 4
1.2.2 CELLULAR COMPONENT 6
1.2.3 BIOLOGICAL PROCESS 12
2 HCOV-CLUSTER_SET 24
2.1 GO CLASSIFICATION 24
2.2 TABLES CLASSIFICATION 26
2.2.1 MOLECULAR FUNCTION 26
2.2.2 CELLULAR COMPONENT 27
2.2.3 BIOLOGICAL PROCESS 29
GO ENRICHMENT: 37
1 HCOV-HOST_SET 38
1.1 GO ENRICHMENT 38
1.2 TABLES ENRICHMENT 41
1.2.1 MOLECULAR FUNCTION 41
1.2.2 CELLULAR COMPONENT 42
1.2.3 BIOLOGICAL PROCESS 43
2 HCOV-CLUSTER_SET 54
2.1 GO ENRICHMENT 54
2.2 TABLES ENRICHMENT 58
2.2.1 MOLECULAR FUNCTION 58
2.2.2 CELLULAR COMPONENT 59
2.2.3 BIOLOGICAL PROCESS 59
PATHWAY ENRICHMENT: 78
1 HCOV-HOST_SET 78
1.1 KEGG PATHWAY ENRICHMENT 79
1.2 WIKI PATHWAY 81
2 HCOV-CLUSTER_SET 84
2.1 KEGG PATHWAY ENRICHMENT 84
2.2 WIKI PATHWAY 88
CLUSTER ANALYSIS 93
1 CLUSTER COMPARISON 93
2 TABLES 95
2.1 GO 95
2.2 KEGG 99
2.3 GO LOCATION 100

GO CLASSIFICATION:

1 HCOV-HOST_SET

1.1 GO CLASSIFICATION

Functional Profile of a gene set at specific GO level. Genes are classified according to their GO distribution at a specific level.

MOLECULAR FUNCTION

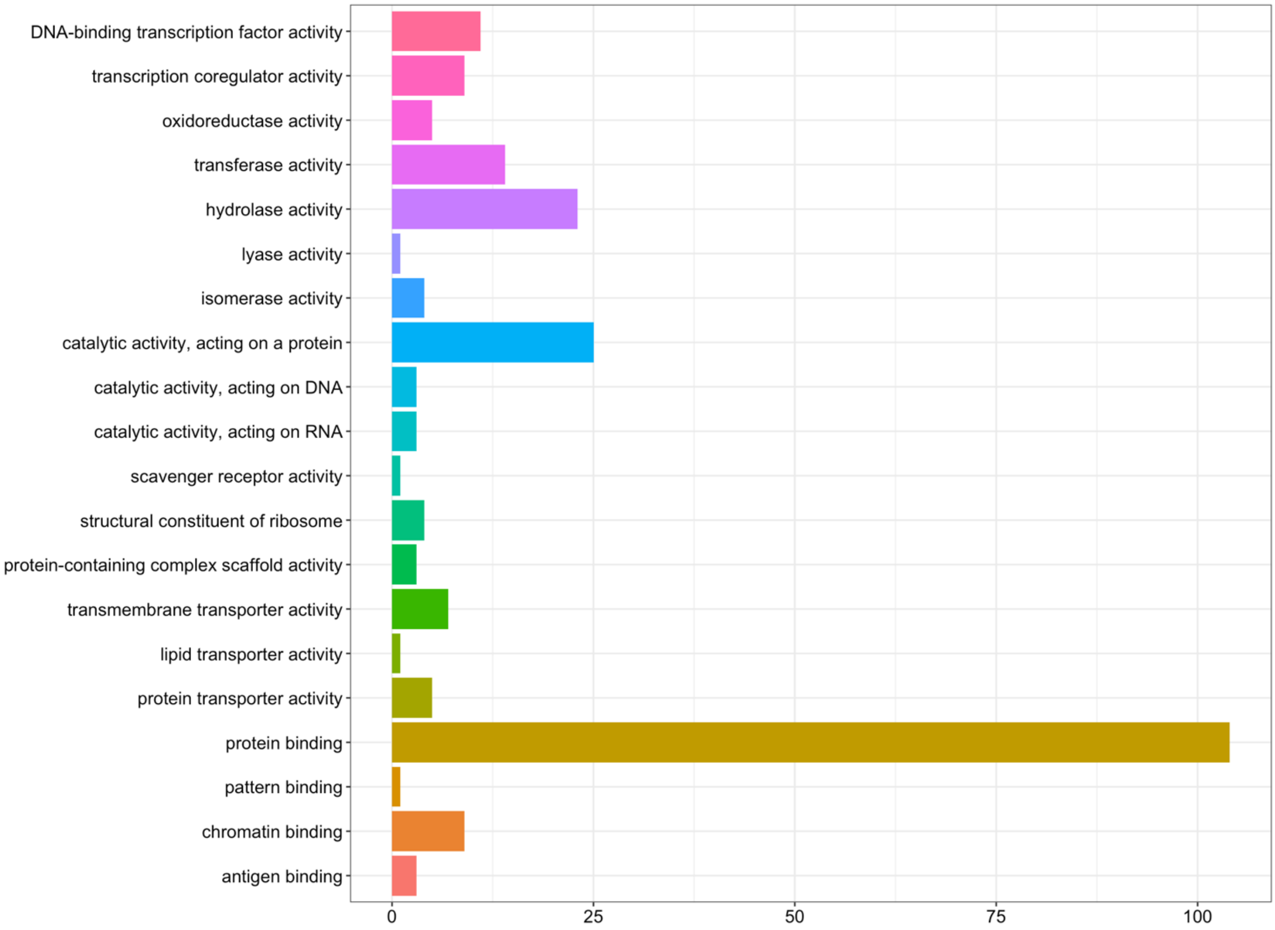

CELLULAR COMPONENT

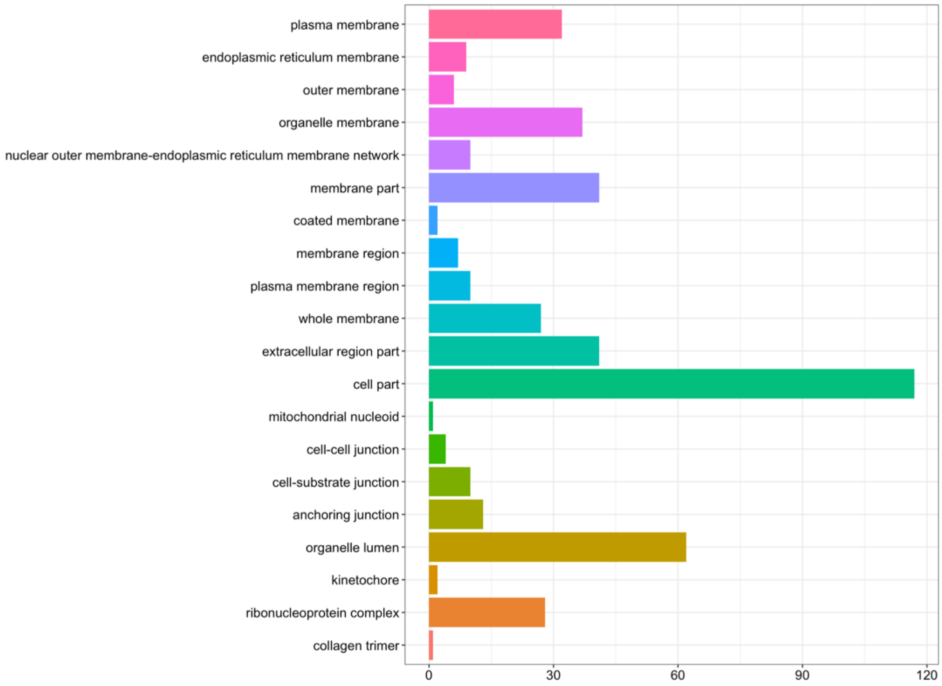

BIOLOGICAL PROCESS

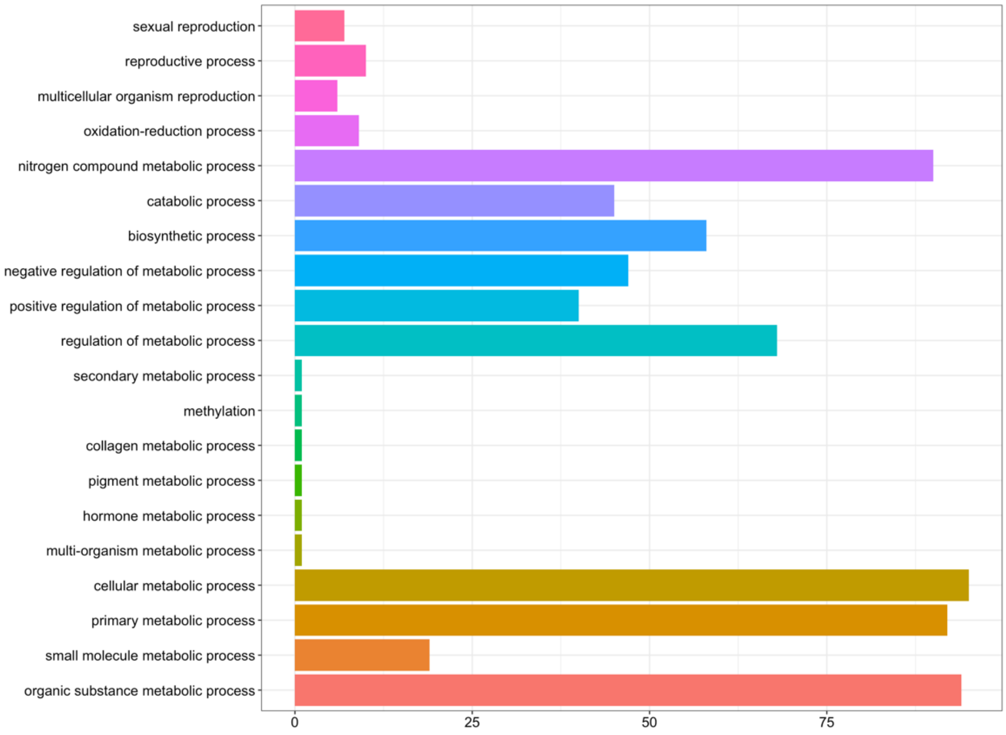

1.2 TABLES CLASSIFICATION

1.2.1 MOLECULAR FUNCTION

ID	Description	GeneRatio	geneID
GO:0003700	DNA-binding transcription factor activity	11/119	TFEB STAT5A PHB JUN STAT3 SMAD3 ATF5 IRF3 NONO PARP1 TERF1
GO:0003712	transcription coregulator activity	9/119	JUN SMAD3 ATF5 IRF3 DNAJB1 NPM1 DDX1 RYBP PFDN5
GO:0016491	oxidoreductase activity	5/119	ND4L COX2 RRM2 NDUFA10 VKORC1
GO:0016740	transferase activity	14/119	YKT6 STAT5A SKP2 IKBKB NMT1 GSK3A GSK3B PARP1 MARK3 MKRN2 MKRN3 CHEK2 TERF1 UBE2I
GO:0016787	hydrolase activity	23/119	PSMC2 PPP1CA FGL2 ACE2 TMPRSS2 ANPEP DPP4 HSPD1 HSPA9 EIF3F KIF11 PSMD1 SRP54 G3BP1 DDX1 DDAH2 TPSAB1 PSMA2 ABHD17A DCTN2 DDX5 EEF1A1 ATP6V1G1
GO:0016829	lyase activity	1/119	ACO2
GO:0016853	isomerase activity	4/119	PPIA FKBP1A PPIG PPIH
GO:0140096	catalytic activity, acting on a protein	25/119	YKT6 STAT5A PPP1CA FGL2 ACE2 PPIA SKP2 TMPRSS2 IKBKB ANPEP DPP4 EIF3F PSMD1 GSK3A GSK3B PARP1 FKBP1A PPIG MARK3 PPIH TPSAB1 PSMA2 ABHD17A CHEK2 UBE2I
GO:0140097	catalytic activity, acting on DNA	3/119	G3BP1 DDX1 TERF1
GO:0140098	catalytic activity, acting on RNA	3/119	G3BP1 DDX1 DDX5
GO:0005044	scavenger receptor activity	1/119	TMPRSS2
GO:0003735	structural constituent of ribosome	4/119	RSL24D1 RPL13A RPL19 RPS20
GO:0032947	protein-containing complex scaffold activity	3/119	G3BP2 CAV1 SGTA
GO:0022857	transmembrane transporter activity	7/119	COX2 MCL1 BCL2 CEACAM1 ANXA2 HNRNPA3 ATP6V1G1
GO:0005319	lipid transporter activity	1/119	CEACAM1
GO:0008565	protein transporter activity	5/119	KPNB1 XPO1 KPNA2 MCL1 KPNA4
GO:0005515	protein binding	104/119	BTF3 SCFD1 PSMC2 TFEB TWF2 YKT6 KPNB1 STX5 STAT5A RSL24D1 PHB JUN STAT3 PPP1CA XPO1 TGFB1 SMAD3 ATF5 COX2 HNRNPA1 ACE2 CLEC4G CD209 CLEC4M IRF3 KPNA2 SFTPD PPIA BCL2L1 BCL2L2 MCL1 BCL2A1 BCL2 SKP2 KPNA4 PRKRA CD9 TMPRSS2 IKBKB DPP4 SYNCRIP PTBP1 CEACAM1 ANXA2 HNRNPA2B1 HNRNPA3 DNAJB1 HSPD1 HSPA9 COPB2 EIF3E EIF3I CHMP4B EIF3F GBF1 RRM2 KIF11 PSMD1 SRP54 NUDCD1 SNX9 NONO GSK3A GSK3B PABPC1 PABPC4 NPM1 G3BP1 G3BP2 RPL19 PARP1 NCL DDX1 RYBP FKBP1A PPIG MARK3 PPIH RCAN3 HGS BAG6 DDAH2 CAMLG CHMP2B SNAP47 MKRN2 TPSAB1 SERPING1 MKRN3 PSMA2 ABHD17A PFDN5 MIF4GD H2AFY2 RPS20 CHEK2 TERF1 DCTN2 DDX5 EEF1A1 CAV1 UBE2I SGTA ATP6V1G1
GO:0001871	pattern binding	1/119	CLEC4G

ID	Description	GeneRatio	geneID
GO:0003682	chromatin binding	9/119	JUN STAT3 SMAD3 ATF5 NONO NPM1 DDX1 H2AFY2 DDX5
GO:0003823	antigen binding	3/119	TGFB1 CD209 CLEC4M
GO:0030246	carbohydrate binding	5/119	CLEC4G CD209 CLEC4M SFTPD NOMO3
GO:0098631	cell adhesion mediator activity	2/119	PPP1CA ANXA2
GO:0008289	lipid binding	8/119	TWF2 ACBD5 SFTPD ANXA2 HSPD1 GBF1 SNX9 CAV1
GO:0008144	drug binding	19/119	PSMC2 TWF2 PPIA IKBKB HSPD1 HSPA9 KIF11 SRP54 GSK3A GSK3B G3BP1 DDX1 FKBP1A PPIG MARK3 PPIH CHEK2 DDX5 UBE2I
GO:0050840	extracellular matrix binding	1/119	ANXA2
GO:0033218	amide binding	11/119	KPNB1 ACBD5 CD209 CLEC4M KPNA2 PPIA ANPEP HSPD1 SRP54 PPIG PPIH
GO:0036094	small molecule binding	23/119	PSMC2 TWF2 ACBD5 CD209 CLEC4M SFTPD IKBKB HSPD1 HSPA9 KIF11 SRP54 GSK3A GSK3B G3BP1 PARP1 DDX1 MARK3 DDAH2 CHEK2 DDX5 EEF1A1 CAV1 UBE2I
GO:0042562	hormone binding	1/119	HSPD1
GO:0043167	ion binding	37/119	PSMC2 TWF2 KPNB1 ACBD5 PPP1CA SMAD3 COX2 ACE2 CD209 CLEC4M IKBKB ANPEP ZCRB1 ANXA2 ACO2 HSPD1 HSPA9 GBF1 RRM2 KIF11 SRP54 SNX9 GSK3A GSK3B G3BP1 PARP1 DDX1 RYBP MARK3 HGS DDAH2 MKRN2 MKRN3 CHEK2 DDX5 EEF1A1 UBE2I
GO:0044877	protein-containing complex binding	15/119	SCFD1 TWF2 PPP1CA SMAD3 CD9 HSPD1 KIF11 SRP54 SNX9 NPM1 FKBP1A PPIH BAG6 DDX5 SGTA
GO:0046790	virion binding	4/119	CD209 CLEC4M PPIA ANXA2
GO:0048037	cofactor binding	4/119	ACBD5 ACO2 PARP1 VKORC1
GO:0051540	metal cluster binding	1/119	ACO2
GO:0060090	molecular adaptor activity	1/119	CAV1
GO:0072341	modified amino acid binding	1/119	ANXA2
GO:0097159	organic cyclic compound binding	64/119	BTF3 PSMC2 TFEB TWF2 KPNB1 STAT5A RSL24D1 ACBD5 PHB JUN STAT3 XPO1 SMAD3 ATF5 HNRNPA1 IRF3 KPNA2 PPIA BCL2 PRKRA IKBKB ZCRB1 SYNCRIP PTBP1 ANXA2 HNRNPA2B1 HNRNPA3 HSPD1 HSPA9 RPL13A EIF3E EIF3I EIF3F KIF11 SRP54 NACA NONO GSK3A GSK3B PABPC1 PABPC4 NPM1 G3BP1 G3BP2 RPL19 PARP1 NCL DDX1 RYBP FKBP1A PPIG MARK3 RCAN3 MKRN2 MIF4GD LAS1L H2AFY2 RPS20 CHEK2 TERF1 DDX5 EEF1A1 CAV1 UBE2I
GO:0097367	carbohydrate derivative binding	18/119	PSMC2 TWF2 ACBD5 SFTPD IKBKB HSPD1 HSPA9 KIF11 SRP54 GSK3A GSK3B G3BP1 DDX1 MARK3 CHEK2 DDX5 EEF1A1 UBE2I
GO:1901363	heterocyclic compound binding	63/119	BTF3 PSMC2 TFEB TWF2 KPNB1 STAT5A RSL24D1 ACBD5 PHB JUN STAT3 XPO1 SMAD3 ATF5 HNRNPA1 IRF3 KPNA2 PPIA BCL2 PRKRA IKBKB ZCRB1 SYNCRIP PTBP1 ANXA2 HNRNPA2B1 HNRNPA3 HSPD1 HSPA9 RPL13A EIF3E EIF3I EIF3F KIF11 SRP54 NACA NONO GSK3A GSK3B PABPC1 PABPC4 NPM1 G3BP1 G3BP2 RPL19 PARP1 NCL DDX1 RYBP FKBP1A PPIG MARK3 RCAN3 MKRN2 MIF4GD LAS1L H2AFY2 RPS20 CHEK2 TERF1 DDX5 EEF1A1 UBE2I
GO:1901567	fatty acid derivative binding	1/119	ACBD5
GO:1901681	sulfur compound binding	1/119	ACBD5
GO:0005085	guanyl-nucleotide exchange factor activity	1/119	GBF1

ID	Description	GeneRatio	geneID
GO:0016247	channel regulator activity	2/119	BCL2 CAV1
GO:0030234	enzyme regulator activity	10/119	JUN TGFB1 PRKRA ANXA2 DNAJB1 PSMD1 NPM1 RCAN3 SERPING1 CAV1
GO:0030545	receptor regulator activity	1/119	TGFB1
GO:0090079	translation regulator activity, nucleic acid binding	2/119	PABPC1 MIF4GD
GO:0038023	signaling receptor activity	2/119	CLEC4M ANPEP
GO:0005072	transforming growth factor beta receptor, cytoplasmic mediator activity	1/119	SMAD3
GO:0001618	virus receptor activity	6/119	ACE2 CLEC4G CD209 CLEC4M ANPEP DPP4
GO:0140142	nucleocytoplasmic carrier activity	3/119	XPO1 KPNA2 KPNA4

1.2.2 CELLULAR COMPONENT

ID	Description	GeneRatio	geneID
GO:0005886	plasma membrane	32/119	SCFD1 YKT6 PHB STAT3 PPP1CA TGFB1 SMAD3 ACE2 CLEC4G CD209 CLEC4M KPNA2 CD9 TMPRSS2 IKBKB ANPEP DPP4 CEACAM1 ANXA2 HSPD1 NMT1 CHMP4B SNX9 GSK3B FKBP1A MARK3 CHMP2B SNAP47 ABHD17A EEF1A1 CAV1 ATP6V1G1
GO:0005789	endoplasmic reticulum membrane	9/119	SCFD1 STX5 SFTPD BCL2 COPB2 NOMO3 FKBP1A VKORC1 CAV1
GO:0019867	outer membrane	6/119	PHB BCL2L1 BCL2L2 MCL1 BCL2A1 BCL2
GO:0031090	organelle membrane	37/119	SCFD1 YKT6 KPNB1 STX5 ACBD5 PHB STAT3 XPO1 SMAD3 ND4L COX2 BCL2L1 BCL2L2 MCL1 BCL2A1 BCL2 KPNA4 CD9 ANPEP DPP4 CEACAM1 ANXA2 HSPD1 COPB2 CHMP4B GBF1 SNX9 FKBP1A HGS CHMP2B SNAP47 ABHD17A NDUFA10 EEF1A1 CAV1 SGTA ATP6V1G1
GO:0042175	nuclear outer membrane-endoplasmic reticulum membrane network	10/119	SCFD1 STX5 XPO1 SFTPD BCL2 COPB2 NOMO3 FKBP1A VKORC1 CAV1
GO:0044425	membrane part	41/119	SCFD1 YKT6 STX5 ACBD5 PHB XPO1 ND4L COX2 ACE2 CLEC4G CD209 CLEC4M KPNA2 SFTPD BCL2L1 MCL1 BCL2 CD9 TMPRSS2 IKBKB ANPEP DPP4 CEACAM1 ANXA2 HSPD1 COPB2 NMT1 CHMP4B SNX9 NOMO3 FKBP1A CAMLG CHMP2B SNAP47 ABHD17A NDUFA10 VKORC1 EEF1A1 CAV1 SGTA ATP6V1G1
GO:0048475	coated membrane	2/119	COPB2 CHMP4B
GO:0098589	membrane region	7/119	XPO1 ACE2 IKBKB DPP4 ANXA2 HSPD1 CAV1
GO:0098590	plasma membrane region	10/119	PHB ACE2 KPNA2 CD9 DPP4 CEACAM1 ANXA2 ABHD17A EEF1A1 CAV1
GO:0098805	whole membrane	27/119	STX5 ACBD5 PHB XPO1 ACE2 BCL2L1 BCL2L2 MCL1 BCL2A1 BCL2 CD9 IKBKB ANPEP DPP4 CEACAM1 ANXA2 HSPD1 COPB2 CHMP4B HGS CHMP2B SNAP47 ABHD17A EEF1A1 CAV1 SGTA ATP6V1G1
GO:0044421	extracellular region part	41/119	TWF2 KPNB1 PHB PPP1CA FGL2 TGFB1 HNRNPA1 ACE2 SFTPD PPIA CD9 TMPRSS2 ANPEP DPP4 PTBP1 CEACAM1 ANXA2 HNRNPA2B1 DNAJB1 HSPD1 HSPA9 EIF3E

ID	Description	GeneRatio	geneID
			EIF3I CHMP4B SNX9 PABPC1 NCL MARK3 HGS BAG6 DDAH2 CHMP2B TPSAB1 SERPING1 PSMA2 H2AFY2 RPS20 DCTN2 DDX5 EEF1A1 ATP6V1G1
GO:0044464	cell part	117/119	BTF3 SCFD1 PSMC2 TFEB TWF2 YKT6 KPNB1 STX5 STAT5A RSL24D1 ACBD5 PHB JUN STAT3 PPP1CA SPECC1 FGL2 XPO1 TGFB1 SMAD3 ATF5 ND4L COX2 HNRNPA1 ACE2 CLEC4G CD209 CLEC4M IRF3 KPNA2 SFTPD PPIA BCL2L1 BCL2L2 MCL1 BCL2A1 BCL2 SKP2 KPNA4 PRKRA CD9 TMPRSS2 IKBKB ANPEP ZCRB1 DPP4 SYNCRIP PTBP1 CEACAM1 ANXA2 HNRNPA2B1 HNRNPA3 ACO2 DNAJB1 HSPD1 HSPA9 COPB2 RPL13A EIF3E EIF3I NMT1 CHMP4B EIF3F GBF1 RRM2 KIF11 PSMD1 SRP54 NUDCD1 NACA SNX9 NONO GSK3A GSK3B PABPC1 PABPC4 NPM1 G3BP1 G3BP2 RPL19 PARP1 NCL DDX1 RYBP NOMO3 FKBP1A PPIG MARK3 PPIH RCAN3 HGS BAG6 DDAH2 CAMLG CHMP2B SNAP47 MKRN2 SERPING1 PSMA2 ABHD17A PFDN5 MIF4GD NDUFA10 VKORC1 LAS1L H2AFY2 RPS20 CHEK2 TERF1 DCTN2 DDX5 C11orf74 EEF1A1 CAV1 UBE2I SGTA ATP6V1G1
GO:0042645	mitochondrial nucleoid	1/119	HSPA9
GO:0005911	cell-cell junction	4/119	PPP1CA DPP4 CEACAM1 ANXA2
GO:0030055	cell-substrate junction	10/119	PPIA CD9 DPP4 HSPA9 RPL13A PABPC1 NPM1 G3BP1 RPL19 CAV1
GO:0070161	anchoring junction	13/119	PPP1CA PPIA CD9 DPP4 CEACAM1 ANXA2 HSPA9 RPL13A PABPC1 NPM1 G3BP1 RPL19 CAV1
GO:0043233	organelle lumen	62/119	PSMC2 KPNB1 STAT5A RSL24D1 ACBD5 PHB JUN STAT3 PPP1CA FGL2 XPO1 TGFB1 SMAD3 ATF5 HNRNPA1 IRF3 KPNA2 PPIA BCL2L1 MCL1 BCL2 SKP2 KPNA4 PRKRA ZCRB1 SYNCRIP PTBP1 ANXA2 HNRNPA2B1 HNRNPA3 ACO2 DNAJB1 HSPD1 HSPA9 RPL13A EIF3E GBF1 PSMD1 SRP54 NUDCD1 NONO GSK3B NPM1 PARP1 NCL DDX1 RYBP PPIG PPIH BAG6 SERPING1 PSMA2 MIF4GD NDUFA10 LAS1L H2AFY2 RPS20 CHEK2 TERF1 DDX5 EEF1A1 UBE2I
GO:0000776	kinetochore	2/119	XPO1 DCTN2
GO:1990904	ribonucleoprotein complex	28/119	PSMC2 KPNB1 RSL24D1 XPO1 HNRNPA1 ZCRB1 SYNCRIP HNRNPA2B1 HNRNPA3 RPL13A EIF3E EIF3I EIF3F SRP54 PABPC1 PABPC4 NPM1 G3BP1 G3BP2 RPL19 NCL DDX1 PPIH MKRN3 PSMA2 LAS1L RPS20 DDX5
GO:0005581	collagen trimer	1/119	SFTPD
GO:0005577	fibrinogen complex	1/119	FGL2
GO:0005643	nuclear pore	1/119	KPNB1
GO:0005667	transcription factor complex	6/119	JUN STAT3 SMAD3 ATF5 NONO PARP1
GO:0005838	proteasome regulatory particle	2/119	PSMC2 PSMD1
GO:0005839	proteasome core complex	1/119	PSMA2
GO:0005852	eukaryotic translation initiation factor 3 complex	3/119	EIF3E EIF3I EIF3F
GO:0005853	eukaryotic translation elongation factor 1 complex	1/119	EEF1A1
GO:0005854	nascent polypeptide-associated complex	1/119	NACA
GO:0005875	microtubule associated complex	2/119	KIF11 DCTN2
GO:0008540	proteasome regulatory particle, base subcomplex	2/119	PSMC2 PSMD1
GO:0016272	prefoldin complex	1/119	PFDN5

ID	Description	GeneRatio	geneID
GO:0017053	transcriptional repressor complex	1/119	JUN
GO:0019773	proteasome core complex, alpha-subunit complex	1/119	PSMA2
GO:0022624	proteasome accessory complex	2/119	PSMC2 PSMD1
GO:0030877	beta-catenin destruction complex	2/119	GSK3A GSK3B
GO:0031082	BLOC complex	1/119	SNAP47
GO:0031519	PcG protein complex	1/119	RYBP
GO:0031941	filamentous actin	1/119	SPECC1
GO:0032993	protein-DNA complex	4/119	NPM1 PARP1 H2AFY2 TERF1
GO:0036452	ESCRT complex	3/119	CHMP4B HGS CHMP2B
GO:0043235	receptor complex	4/119	SMAD3 IKBKB CEACAM1 HSPD1
GO:0044815	DNA packaging complex	1/119	H2AFY2
GO:0070069	cytochrome complex	1/119	COX2
GO:0070937	CRD-mediated mRNA stability complex	1/119	SYNCRIP
GO:0071141	SMAD protein complex	1/119	SMAD3
GO:0072379	ER membrane insertion complex	2/119	BAG6 SGTA
GO:0072669	tRNA-splicing ligase complex	1/119	DDX1
GO:0097136	Bcl-2 family protein complex	3/119	BCL2L1 BCL2L2 MCL1
GO:0097452	GAIT complex	2/119	SYNCRIP RPL13A
GO:0098796	membrane protein complex	16/119	YKT6 STX5 ND4L COX2 BCL2 IKBKB CEACAM1 HSPD1 COPB2 CHMP4B FKBP1A CHMP2B SNAP47 NDUFA10 CAV1 ATP6V1G1
GO:0098798	mitochondrial protein complex	3/119	ND4L COX2 NDUFA10
GO:0120114	Sm-like protein family complex	1/119	PPIH
GO:1902494	catalytic complex	18/119	PSMC2 PPP1CA ND4L HNRNPA1 SKP2 PRKRA IKBKB SYNCRIP HNRNPA2B1 HNRNPA3 PSMD1 PABPC1 PSMA2 NDUFA10 LAS1L DCTN2 DDX5 UBE2I
GO:1990351	transporter complex	1/119	FKBP1A
GO:1990356	sumoylated E2 ligase complex	1/119	UBE2I
GO:1990667	PCSK9-AnxA2 complex	1/119	ANXA2

ID	Description	GeneRatio	geneID
GO:1990909	Wnt signalosome	1/119	GSK3B
GO:0005929	cilium	2/119	C11orf74 CAV1
GO:0043227	membrane-bounded organelle	107/119	BTF3 SCFD1 PSMC2 TFEB TWF2 YKT6 KPNB1 STX5 STAT5A RSL24D1 ACBD5 PHB JUN STAT3 PPP1CA SPECC1 FGL2 XPO1 TGFB1 SMAD3 ATF5 ND4L COX2 HNRNPA1 ACE2 IRF3 KPNA2 SFTPD PPIA BCL2L1 BCL2L2 MCL1 BCL2A1 BCL2 SKP2 KPNA4 PRKRA CD9 TMPRSS2 IKBKB ANPEP ZCRB1 DPP4 SYNCRIP PTBP1 CEACAM1 ANXA2 HNRNPA2B1 HNRNPA3 ACO2 DNAJB1 HSPD1 HSPA9 COPB2 RPL13A EIF3E EIF3I NMT1 CHMP4B GBF1 KIF11 PSMD1 SRP54 NUDCD1 NACA SNX9 NONO GSK3A GSK3B PABPC1 PABPC4 NPM1 G3BP1 PARP1 NCL DDX1 RYBP NOMO3 FKBP1A PPIG MARK3 PPIH HGS BAG6 DDAH2 CAMLG CHMP2B SNAP47 SERPING1 PSMA2 ABHD17A PFDN5 MIF4GD NDUFA10 VKORC1 LAS1L H2AFY2 RPS20 CHEK2 TERF1 DCTN2 DDX5 EEF1A1 CAV1 UBE2I SGTA ATP6V1G1
GO:0043228	non-membrane-bounded organelle	47/119	PSMC2 TWF2 KPNB1 RSL24D1 JUN STAT3 PPP1CA SPECC1 XPO1 SMAD3 ATF5 BCL2L1 SKP2 PTBP1 ANXA2 HNRNPA2B1 HNRNPA3 DNAJB1 HSPA9 RPL13A EIF3E GBF1 KIF11 NONO GSK3B PABPC1 PABPC4 NPM1 G3BP1 RPL19 PARP1 NCL DDX1 FKBP1A DDAH2 PSMA2 MIF4GD LAS1L H2AFY2 RPS20 CHEK2 TERF1 DCTN2 DDX5 EEF1A1 CAV1 UBE2I
GO:0043229	intracellular organelle	104/119	BTF3 SCFD1 PSMC2 TFEB TWF2 YKT6 KPNB1 STX5 STAT5A RSL24D1 ACBD5 PHB JUN STAT3 PPP1CA SPECC1 FGL2 XPO1 TGFB1 SMAD3 ATF5 ND4L COX2 HNRNPA1 IRF3 KPNA2 SFTPD PPIA BCL2L1 BCL2L2 MCL1 BCL2A1 BCL2 SKP2 KPNA4 PRKRA CD9 IKBKB ANPEP ZCRB1 DPP4 SYNCRIP PTBP1 CEACAM1 ANXA2 HNRNPA2B1 HNRNPA3 ACO2 DNAJB1 HSPD1 HSPA9 COPB2 RPL13A EIF3E NMT1 CHMP4B GBF1 KIF11 PSMD1 SRP54 NUDCD1 NACA SNX9 NONO GSK3A GSK3B PABPC1 PABPC4 NPM1 G3BP1 RPL19 PARP1 NCL DDX1 RYBP NOMO3 FKBP1A PPIG PPIH HGS BAG6 DDAH2 CAMLG CHMP2B SNAP47 SERPING1 PSMA2 ABHD17A PFDN5 MIF4GD NDUFA10 VKORC1 LAS1L H2AFY2 RPS20 CHEK2 TERF1 DCTN2 DDX5 EEF1A1 CAV1 UBE2I SGTA ATP6V1G1
GO:0043230	extracellular organelle	38/119	TWF2 KPNB1 PHB PPP1CA FGL2 HNRNPA1 ACE2 PPIA CD9 TMPRSS2 ANPEP DPP4 PTBP1 CEACAM1 ANXA2 HNRNPA2B1 DNAJB1 HSPD1 HSPA9 EIF3E EIF3I CHMP4B SNX9 PABPC1 NCL MARK3 HGS BAG6 DDAH2 CHMP2B SERPING1 PSMA2 H2AFY2 RPS20 DCTN2 DDX5 EEF1A1 ATP6V1G1
GO:0044422	organelle part	94/119	SCFD1 PSMC2 TWF2 YKT6 KPNB1 STX5 STAT5A RSL24D1 ACBD5 PHB JUN STAT3 PPP1CA SPECC1 FGL2 XPO1 TGFB1 SMAD3 ATF5 ND4L COX2 HNRNPA1 IRF3 KPNA2 SFTPD PPIA BCL2L1 BCL2L2 MCL1 BCL2A1 BCL2 SKP2 KPNA4 PRKRA CD9 ANPEP ZCRB1 DPP4 SYNCRIP PTBP1 CEACAM1 ANXA2 HNRNPA2B1 HNRNPA3 ACO2 DNAJB1 HSPD1 HSPA9 COPB2 RPL13A EIF3E CHMP4B GBF1 KIF11 PSMD1 SRP54 NUDCD1 SNX9 NONO GSK3B PABPC1 NPM1 RPL19 PARP1 NCL DDX1 RYBP NOMO3 FKBP1A PPIG PPIH HGS BAG6 DDAH2 CHMP2B SNAP47 SERPING1 PSMA2 ABHD17A MIF4GD NDUFA10 VKORC1 LAS1L H2AFY2 RPS20 CHEK2 TERF1 DCTN2 DDX5 EEF1A1 CAV1 UBE2I SGTA ATP6V1G1
GO:0099572	postsynaptic specialization	7/119	PHB STAT3 KPNA2 HNRNPA3 DNAJB1 CHMP2B ABHD17A
GO:0018995	host	4/119	KPNB1 XPO1 CD209 CLEC4M
GO:0044217	other organism part	4/119	KPNB1 XPO1 CD209 CLEC4M
GO:0044216	other organism cell	4/119	KPNB1 XPO1 CD209 CLEC4M
GO:0005615	extracellular space	41/119	TWF2 KPNB1 PHB PPP1CA FGL2 TGFB1 HNRNPA1 ACE2 SFTPD PPIA CD9 TMPRSS2 ANPEP DPP4 PTBP1 CEACAM1 ANXA2 HNRNPA2B1 DNAJB1 HSPD1 HSPA9 EIF3E EIF3I CHMP4B SNX9 PABPC1 NCL MARK3 HGS BAG6 DDAH2 CHMP2B TPSAB1 SERPING1 PSMA2 H2AFY2 RPS20 DCTN2 DDX5 EEF1A1 ATP6V1G1
GO:0031012	extracellular matrix	4/119	TGFB1 ANXA2 TPSAB1 SERPING1
GO:0072562	blood microparticle	2/119	TGFB1 SERPING1
GO:0031300	intrinsic component of organelle membrane	1/119	ABHD17A
GO:0032420	stereocilium	1/119	TWF2

ID	Description	GeneRatio	geneID
GO:0044446	intracellular organelle part	94/119	SCFD1 PSMC2 TWF2 YKT6 KPNB1 STX5 STAT5A RSL24D1 ACBD5 PHB JUN STAT3 PPP1CA SPECC1 FGL2 XPO1 TGFB1 SMAD3 ATF5 ND4L COX2 HNRNPA1 IRF3 KPNA2 SFTPD PPIA BCL2L1 BCL2L2 MCL1 BCL2A1 BCL2 SKP2 KPNA4 PRKRA CD9 ANPEP ZCRB1 DPP4 SYNCRIP PTBP1 CEACAM1 ANXA2 HNRNPA2B1 HNRNPA3 ACO2 DNAJB1 HSPD1 HSPA9 COPB2 RPL13A EIF3E CHMP4B GBF1 KIF11 PSMD1 SRP54 NUDCD1 SNX9 NONO GSK3B PABPC1 NPM1 RPL19 PARP1 NCL DDX1 RYBP NOMO3 FKBP1A PPIG PPIH HGS BAG6 DDAH2 CHMP2B SNAP47 SERPING1 PSMA2 ABHD17A MIF4GD NDUFA10 VKORC1 LAS1L H2AFY2 RPS20 CHEK2 TERF1 DCTN2 DDX5 EEF1A1 CAV1 UBE2I SGTA ATP6V1G1
GO:0044449	contractile fiber part	1/119	FKBP1A
GO:0098892	extrinsic component of postsynaptic specialization membrane	1/119	KPNA2
GO:0098948	intrinsic component of postsynaptic specialization membrane	1/119	ABHD17A
GO:0099634	postsynaptic specialization membrane	2/119	KPNA2 ABHD17A
GO:0019898	extrinsic component of membrane	7/119	PHB KPNA2 ANXA2 NMT1 SNX9 FKBP1A SGTA
GO:0005905	clathrin-coated pit	1/119	HSPD1
GO:0031224	intrinsic component of membrane	26/119	YKT6 STX5 ACBD5 PHB ND4L COX2 ACE2 CLEC4G CD209 CLEC4M BCL2L1 MCL1 BCL2 CD9 TMPRSS2 IKBKB ANPEP DPP4 CEACAM1 HSPD1 NOMO3 FKBP1A CAMLG ABHD17A VKORC1 CAV1
GO:0044455	mitochondrial membrane part	5/119	PHB ND4L COX2 HSPD1 NDUFA10
GO:0044459	plasma membrane part	19/119	YKT6 PHB ACE2 CD209 CLEC4M KPNA2 CD9 TMPRSS2 IKBKB ANPEP DPP4 CEACAM1 ANXA2 CHMP4B SNX9 FKBP1A ABHD17A EEF1A1 CAV1
GO:0070469	respiratory chain	3/119	ND4L COX2 NDUFA10
GO:0098552	side of membrane	8/119	CD209 CD9 IKBKB ANPEP CHMP4B SNX9 FKBP1A EEF1A1
GO:0008021	synaptic vesicle	3/119	BCL2L1 SNAP47 SGTA
GO:0030672	synaptic vesicle membrane	3/119	BCL2L1 SNAP47 SGTA
GO:0048786	presynaptic active zone	1/119	PHB
GO:0097060	synaptic membrane	3/119	PHB KPNA2 ABHD17A
GO:0098793	presynapse	5/119	PHB PPP1CA BCL2L1 SNAP47 SGTA
GO:0098794	postsynapse	11/119	PSMC2 PHB STAT3 PPP1CA KPNA2 HNRNPA3 DNAJB1 GSK3A GSK3B CHMP2B ABHD17A
GO:0098845	postsynaptic endosome	1/119	ABHD17A
GO:0098850	extrinsic component of synaptic vesicle membrane	1/119	SGTA
GO:0098895	postsynaptic endosome membrane	1/119	ABHD17A
GO:0098949	intrinsic component of postsynaptic endosome membrane	1/119	ABHD17A

ID	Description	GeneRatio	geneID
GO:0099240	intrinsic component of synaptic membrane	1/119	ABHD17A
GO:0099243	extrinsic component of synaptic membrane	2/119	PHB KPNA2
GO:0005622	intracellular	114/119	BTF3 SCFD1 PSMC2 TFEB TWF2 YKT6 KPNB1 STX5 STAT5A RSL24D1 ACBD5 PHB JUN STAT3 PPP1CA SPECC1 FGL2 XPO1 TGFB1 SMAD3 ATF5 ND4L COX2 HNRNPA1 ACE2 CD209 CLEC4M IRF3 KPNA2 SFTPD PPIA BCL2L1 BCL2L2 MCL1 BCL2A1 BCL2 SKP2 KPNA4 PRKRA CD9 IKBKB ANPEP ZCRB1 DPP4 SYNCRIP PTBP1 CEACAM1 ANXA2 HNRNPA2B1 HNRNPA3 ACO2 DNAJB1 HSPD1 HSPA9 COPB2 RPL13A EIF3E EIF3I NMT1 CHMP4B EIF3F GBF1 RRM2 KIF11 PSMD1 SRP54 NUDCD1 NACA SNX9 NONO GSK3A GSK3B PABPC1 PABPC4 NPM1 G3BP1 G3BP2 RPL19 PARP1 NCL DDX1 RYBP NOMO3 FKBP1A PPIG MARK3 PPIH RCAN3 HGS BAG6 DDAH2 CAMLG CHMP2B SNAP47 MKRN2 SERPING1 PSMA2 ABHD17A PFDN5 MIF4GD NDUFA10 VKORC1 LAS1L H2AFY2 RPS20 CHEK2 TERF1 DCTN2 DDX5 EEF1A1 CAV1 UBE2I SGTA ATP6V1G1
GO:0005642	annulate lamellae	1/119	XPO1
GO:0008287	protein serine/threonine phosphatase complex	1/119	PPP1CA
GO:0009986	cell surface	10/119	PHB TGFB1 ACE2 CD209 CD9 ANPEP DPP4 CEACAM1 ANXA2 HSPD1
GO:0012505	endomembrane system	42/119	SCFD1 PSMC2 YKT6 KPNB1 STX5 PHB FGL2 XPO1 TGFB1 SMAD3 SFTPD PPIA BCL2L1 BCL2 KPNA4 CD9 ANPEP SYNCRIP CEACAM1 ANXA2 HSPD1 COPB2 CHMP4B GBF1 PSMD1 SNX9 PARP1 NOMO3 FKBP1A HGS CAMLG CHMP2B SNAP47 SERPING1 PSMA2 ABHD17A VKORC1 CHEK2 EEF1A1 CAV1 UBE2I SGTA
GO:0030427	site of polarized growth	2/119	TWF2 DCTN2
GO:0030496	midbody	2/119	ANXA2 CHMP4B
GO:0031252	cell leading edge	6/119	TWF2 DPP4 ANXA2 GBF1 SNX9 EEF1A1
GO:0031975	envelope	18/119	KPNB1 PHB STAT3 XPO1 SMAD3 ND4L COX2 BCL2L1 BCL2L2 MCL1 BCL2A1 BCL2 KPNA4 HSPD1 CHMP4B PARP1 NDUFA10 UBE2I
GO:0035749	myelin sheath adaxonal region	1/119	ANXA2
GO:0042995	cell projection	22/119	PSMC2 TWF2 YKT6 PPP1CA TGFB1 ACE2 DPP4 CEACAM1 ANXA2 HNRNPA3 DNAJB1 SNX9 GSK3A GSK3B MARK3 SNAP47 ABHD17A DCTN2 C11orf74 EEF1A1 CAV1 UBE2I
GO:0043209	myelin sheath	6/119	PHB BCL2 ANXA2 ACO2 HSPD1 NDUFA10
GO:0043218	compact myelin	1/119	ANXA2
GO:0043220	Schmidt-Lanterman incisure	1/119	ANXA2
GO:0044297	cell body	7/119	YKT6 PPP1CA TGFB1 DNAJB1 GSK3A G3BP1 SNAP47
GO:0044424	intracellular part	113/119	BTF3 SCFD1 PSMC2 TFEB TWF2 YKT6 KPNB1 STX5 STAT5A RSL24D1 ACBD5 PHB JUN STAT3 PPP1CA SPECC1 FGL2 XPO1 TGFB1 SMAD3 ATF5 ND4L COX2 HNRNPA1 ACE2 CD209 CLEC4M IRF3 KPNA2 SFTPD PPIA BCL2L1 BCL2L2 MCL1 BCL2A1 BCL2 SKP2 KPNA4 PRKRA CD9 IKBKB ANPEP ZCRB1 DPP4 SYNCRIP PTBP1 CEACAM1 ANXA2 HNRNPA2B1 HNRNPA3 ACO2 DNAJB1 HSPD1 HSPA9 COPB2 RPL13A EIF3E EIF3I NMT1 CHMP4B EIF3F GBF1 RRM2 KIF11 PSMD1 SRP54 NUDCD1 NACA SNX9 NONO GSK3A GSK3B PABPC1 PABPC4 NPM1 G3BP1 G3BP2 RPL19 PARP1 NCL DDX1 RYBP NOMO3 FKBP1A PPIG MARK3 PPIH RCAN3 HGS BAG6 DDAH2 CAMLG CHMP2B SNAP47 SERPING1 PSMA2 ABHD17A PFDN5 MIF4GD NDUFA10 VKORC1 LAS1L H2AFY2 RPS20 CHEK2 TERF1 DCTN2 DDX5 EEF1A1 CAV1 UBE2I SGTA ATP6V1G1
GO:0044463	cell projection part	16/119	PSMC2 TWF2 YKT6 PPP1CA ACE2 DPP4 CEACAM1 DNAJB1 GSK3A GSK3B MARK3 SNAP47 ABHD17A DCTN2 EEF1A1 UBE2I

ID	Description	GeneRatio	geneID
GO:0045177	apical part of cell	3/119	CD9 DPP4 CEACAM1
GO:0045178	basal part of cell	1/119	CEACAM1
GO:0071944	cell periphery	33/119	SCFD1 YKT6 PHB STAT3 PPP1CA TGFB1 SMAD3 ACE2 CLEC4G CD209 CLEC4M KPNA2 CD9 TMPRSS2 IKBKB ANPEP DPP4 CEACAM1 ANXA2 HSPD1 NMT1 CHMP4B SNX9 GSK3B NCL FKBP1A MARK3 CHMP2B SNAP47 ABHD17A EEF1A1 CAV1 ATP6V1G1
GO:0097223	sperm part	2/119	DNAJB1 CAV1
GO:0097458	neuron part	20/119	PSMC2 TWF2 YKT6 PHB STAT3 PPP1CA TGFB1 BCL2L1 HNRNPA3 DNAJB1 GSK3A GSK3B G3BP1 MARK3 CHMP2B SNAP47 ABHD17A DCTN2 UBE2I SGTA
GO:0098862	cluster of actin-based cell projections	2/119	TWF2 ACE2
GO:1990204	oxidoreductase complex	2/119	ND4L NDUFA10
GO:0044456	synapse part	14/119	PSMC2 PHB STAT3 PPP1CA KPNA2 BCL2L1 HNRNPA3 DNAJB1 GSK3A GSK3B CHMP2B SNAP47 ABHD17A SGTA
GO:0098685	Schaffer collateral - CA1 synapse	1/119	STAT3
GO:0098978	glutamatergic synapse	9/119	PHB STAT3 PPP1CA KPNA2 DNAJB1 GSK3B CHMP2B SNAP47 ABHD17A
GO:0098982	GABA-ergic synapse	1/119	PHB
GO:0098984	neuron to neuron synapse	6/119	PHB STAT3 HNRNPA3 DNAJB1 CHMP2B ABHD17A
GO:0099081	supramolecular polymer	5/119	TWF2 SPECC1 KIF11 FKBP1A DCTN2

1.2.3 BIOLOGICAL PROCESS

ID	Description	GeneRatio	geneID
GO:0019953	sexual reproduction	7/119	STAT3 TGFB1 BCL2L1 BCL2L2 BCL2 CD9 BAG6
GO:0022414	reproductive process	10/119	TFEB PHB STAT3 TGFB1 BCL2L1 BCL2L2 BCL2 CD9 BAG6 TERF1
GO:0032504	multicellular organism reproduction	6/119	TGFB1 BCL2L1 BCL2L2 BCL2 CD9 BAG6
GO:0055114	oxidation-reduction process	9/119	PPP1CA ND4L COX2 ACO2 RRM2 GSK3A GSK3B NDUFA10 VKORC1
GO:0006807	nitrogen compound metabolic process	90/119	BTF3 PSMC2 TFEB KPNB1 STX5 STAT5A RSL24D1 PHB JUN STAT3 PPP1CA FGL2 XPO1 TGFB1 SMAD3 ATF5 ND4L COX2 HNRNPA1 ACE2 IRF3 KPNA2 SFTPD PPIA BCL2 SKP2 PRKRA TMPRSS2 IKBKB ANPEP ZCRB1 DPP4 SYNCRIP PTBP1 CEACAM1 ANXA2 HNRNPA2B1 HNRNPA3 DNAJB1 HSPD1 RPL13A EIF3E EIF3I NMT1 CHMP4B EIF3F RRM2 PSMD1 SNX9 NONO GSK3A GSK3B PABPC1 PABPC4 NPM1 G3BP1 G3BP2 RPL19 PARP1 NCL DDX1 RYBP FKBP1A PPIG MARK3 PPIH RCAN3 HGS BAG6 DDAH2 MKRN2 TPSAB1 SERPING1 MKRN3 PSMA2 ABHD17A PFDN5 MIF4GD NDUFA10 VKORC1 LAS1L H2AFY2 RPS20 CHEK2 TERF1 DDX5 EEF1A1 CAV1 UBE2I SGTA
GO:0009056	catabolic process	45/119	SCFD1 PSMC2 TFEB KPNB1 STX5 ACBD5 PHB STAT3 PPP1CA XPO1 TGFB1 SMAD3 MCL1 BCL2 SKP2 ANPEP SYNCRIP CEACAM1 ANXA2 RPL13A EIF3E CHMP4B PSMD1 SNX9 GSK3A GSK3B PABPC1 PABPC4 NPM1 RPL19 RYBP HGS

ID	Description	GeneRatio	geneID
			BAG6 DDAH2 CHMP2B PSMA2 ABHD17A RPS20 CHEK2 DDX5 EEF1A1 CAV1 UBE2I SGTA ATP6V1G1
GO:0009058	biosynthetic process	58/119	BTF3 PSMC2 TFEB KPNB1 STAT5A RSL24D1 PHB JUN STAT3 PPP1CA XPO1 TGFB1 SMAD3 ATF5 COX2 HNRNPA1 ACE2 IRF3 SFTPD PPIA BCL2 IKBKB SYNCRIP CEACAM1 HNRNPA2B1 DNAJB1 HSPD1 RPL13A EIF3E EIF3I NMT1 EIF3F GBF1 RRM2 NONO GSK3A GSK3B PABPC1 PABPC4 NPM1 G3BP2 RPL19 PARP1 NCL DDX1 RYBP DDAH2 ABHD17A PFDN5 MIF4GD H2AFY2 RPS20 CHEK2 TERF1 DDX5 EEF1A1 CAV1 UBE2I
GO:0009892	negative regulation of metabolic process	47/119	SCFD1 PHB JUN STAT3 XPO1 TGFB1 SMAD3 ATF5 HNRNPA1 IRF3 SFTPD MCL1 BCL2 PRKRA IKBKB SYNCRIP PTBP1 CEACAM1 ANXA2 HNRNPA2B1 DNAJB1 HSPD1 RPL13A EIF3E CHMP4B NONO GSK3A GSK3B PABPC1 PABPC4 NPM1 RPL19 PARP1 NCL RYBP FKBP1A BAG6 SERPING1 PFDN5 H2AFY2 RPS20 CHEK2 TERF1 DDX5 CAV1 UBE2I SGTA
GO:0009893	positive regulation of metabolic process	40/119	PSMC2 TFEB STX5 PHB JUN STAT3 TGFB1 SMAD3 ATF5 COX2 HNRNPA1 ACE2 IRF3 BCL2 SKP2 IKBKB SYNCRIP PTBP1 ANXA2 HNRNPA2B1 HSPD1 EIF3E SNX9 GSK3A GSK3B PABPC1 NPM1 PARP1 NCL RYBP FKBP1A HGS BAG6 DDAH2 MIF4GD CHEK2 DDX5 CAV1 UBE2I SGTA
GO:0019222	regulation of metabolic process	68/119	SCFD1 PSMC2 TFEB KPNB1 STX5 STAT5A PHB JUN STAT3 PPP1CA XPO1 TGFB1 SMAD3 ATF5 COX2 HNRNPA1 ACE2 IRF3 KPNA2 SFTPD MCL1 BCL2 SKP2 PRKRA IKBKB SYNCRIP PTBP1 CEACAM1 ANXA2 HNRNPA2B1 DNAJB1 HSPD1 RPL13A EIF3E CHMP4B RRM2 PSMD1 SNX9 NONO GSK3A GSK3B PABPC1 PABPC4 NPM1 G3BP2 RPL19 PARP1 NCL DDX1 RYBP FKBP1A RCAN3 HGS BAG6 DDAH2 SERPING1 PFDN5 MIF4GD H2AFY2 RPS20 CHEK2 TERF1 DDX5 EEF1A1 CAV1 UBE2I SGTA ATP6V1G1
GO:0019748	secondary metabolic process	1/119	BCL2
GO:0032259	methylation	1/119	PARP1
GO:0032963	collagen metabolic process	1/119	TGFB1
GO:0042440	pigment metabolic process	1/119	BCL2
GO:0042445	hormone metabolic process	1/119	ACE2
GO:0044033	multi-organism metabolic process	1/119	ANXA2
GO:0044237	cellular metabolic process	95/119	BTF3 SCFD1 PSMC2 TFEB KPNB1 STAT5A RSL24D1 ACBD5 PHB JUN STAT3 PPP1CA XPO1 TGFB1 SMAD3 ATF5 ND4L COX2 HNRNPA1 ACE2 IRF3 KPNA2 SFTPD PPIA MCL1 BCL2 SKP2 PRKRA CD9 IKBKB ANPEP ZCRB1 SYNCRIP PTBP1 CEACAM1 ANXA2 HNRNPA2B1 HNRNPA3 ACO2 DNAJB1 HSPD1 HSPA9 RPL13A EIF3E EIF3I NMT1 CHMP4B EIF3F GBF1 RRM2 PSMD1 SNX9 NONO GSK3A GSK3B PABPC1 PABPC4 NPM1 G3BP1 G3BP2 RPL19 PARP1 NCL DDX1 RYBP FKBP1A PPIG MARK3 PPIH RCAN3 HGS BAG6 DDAH2 CAMLG CHMP2B MKRN2 SERPING1 MKRN3 PSMA2 ABHD17A PFDN5 MIF4GD NDUFA10 VKORC1 LAS1L H2AFY2 RPS20 CHEK2 TERF1 DDX5 EEF1A1 CAV1 UBE2I SGTA ATP6V1G1
GO:0044238	primary metabolic process	92/119	BTF3 PSMC2 TFEB KPNB1 STX5 STAT5A RSL24D1 ACBD5 PHB JUN STAT3 PPP1CA FGL2 XPO1 TGFB1 SMAD3 ATF5 ND4L COX2 HNRNPA1 ACE2 IRF3 KPNA2 SFTPD PPIA BCL2 SKP2 PRKRA TMPRSS2 IKBKB ANPEP ZCRB1 DPP4 SYNCRIP PTBP1 CEACAM1 ANXA2 HNRNPA2B1 HNRNPA3 ACO2 DNAJB1 HSPD1 RPL13A EIF3E EIF3I NMT1 CHMP4B EIF3F RRM2 PSMD1 SNX9 NONO GSK3A GSK3B PABPC1 PABPC4 NPM1 G3BP1 G3BP2 RPL19 PARP1 NCL DDX1 RYBP FKBP1A PPIG MARK3 PPIH RCAN3 HGS BAG6 DDAH2 MKRN2 TPSAB1 SERPING1 MKRN3 PSMA2 ABHD17A PFDN5 MIF4GD NDUFA10 VKORC1 LAS1L H2AFY2 RPS20 CHEK2 TERF1 DDX5 EEF1A1 CAV1 UBE2I SGTA
GO:0044281	small molecule metabolic process	19/119	KPNB1 STAT5A ACBD5 STAT3 PPP1CA TGFB1 ND4L COX2 CEACAM1 ACO2 NMT1 RRM2 GSK3A GSK3B PARP1 DDAH2 NDUFA10 VKORC1 CAV1
GO:0071704	organic substance metabolic process	94/119	BTF3 PSMC2 TFEB KPNB1 STX5 STAT5A RSL24D1 ACBD5 PHB JUN STAT3 PPP1CA FGL2 XPO1 TGFB1 SMAD3 ATF5 ND4L COX2 HNRNPA1 ACE2 IRF3 KPNA2 SFTPD PPIA BCL2 SKP2 PRKRA CD9 TMPRSS2 IKBKB ANPEP ZCRB1 DPP4 SYNCRIP PTBP1 CEACAM1 ANXA2 HNRNPA2B1 HNRNPA3 ACO2 DNAJB1 HSPD1 RPL13A EIF3E EIF3I NMT1 CHMP4B EIF3F RRM2 PSMD1 SNX9 NONO GSK3A GSK3B PABPC1 PABPC4 NPM1 G3BP1 G3BP2 RPL19 PARP1 NCL DDX1

ID	Description	GeneRatio	geneID
			RYBP FKBP1A PPIG MARK3 PPIH RCAN3 HGS BAG6 DDAH2 CAMLG MKRN2 TPSAB1 SERPING1 MKRN3 PSMA2 ABHD17A PFDN5 MIF4GD NDUFA10 VKORC1 LAS1L H2AFY2 RPS20 CHEK2 TERF1 DDX5 EEF1A1 CAV1 UBE2I SGTA
GO:1990845	adaptive thermogenesis	1/119	CAV1
GO:0001909	leukocyte mediated cytotoxicity	1/119	CEACAM1
GO:0031341	regulation of cell killing	1/119	CEACAM1
GO:0031342	negative regulation of cell killing	1/119	CEACAM1
GO:0001776	leukocyte homeostasis	2/119	TGFB1 BCL2
GO:0002200	somatic diversification of immune receptors	2/119	TGFB1 HSPD1
GO:0002252	immune effector process	21/119	PSMC2 KPNB1 PHB STAT3 FGL2 TGFB1 CLEC4G IRF3 PPIA BCL2 SKP2 ANPEP CEACAM1 ANXA2 HSPD1 GBF1 PSMD1 DDX1 SERPING1 PSMA2 EEF1A1
GO:0002253	activation of immune response	12/119	PHB CD209 IRF3 SFTPD BCL2 IKBKB CEACAM1 HSPD1 NONO BAG6 SERPING1 CAV1
GO:0002262	myeloid cell homeostasis	2/119	STAT3 HSPA9
GO:0002440	production of molecular mediator of immune response	3/119	TGFB1 HSPD1 DDX1
GO:0002507	tolerance induction	1/119	TGFB1
GO:0002520	immune system development	11/119	JUN STAT3 TGFB1 SMAD3 BCL2 CEACAM1 ANXA2 HSPD1 HSPA9 PABPC4 PARP1
GO:0002682	regulation of immune system process	21/119	PHB JUN STAT3 TGFB1 SMAD3 CLEC4G CD209 IRF3 SFTPD BCL2 IKBKB DPP4 CEACAM1 HSPD1 HSPA9 NONO DDX1 FKBP1A BAG6 SERPING1 CAV1
GO:0002683	negative regulation of immune system process	6/119	TGFB1 CLEC4G SFTPD CEACAM1 HSPA9 SERPING1
GO:0002684	positive regulation of immune system process	17/119	PHB JUN STAT3 TGFB1 CD209 IRF3 SFTPD BCL2 IKBKB DPP4 CEACAM1 HSPD1 NONO DDX1 BAG6 SERPING1 CAV1
GO:0006955	immune response	35/119	PSMC2 TFEB KPNB1 PHB JUN STAT3 FGL2 TGFB1 SMAD3 CLEC4G CD209 CLEC4M IRF3 SFTPD PPIA BCL2 SKP2 PRKRA IKBKB ANPEP SYNCRIP CEACAM1 ANXA2 HSPD1 RPL13A GBF1 PSMD1 NONO DDX1 FKBP1A BAG6 SERPING1 PSMA2 EEF1A1 CAV1
GO:0019882	antigen processing and presentation	5/119	CD209 CLEC4M IKBKB KIF11 DCTN2
GO:0031294	lymphocyte costimulation	2/119	DPP4 CAV1
GO:0045058	T cell selection	2/119	STAT3 BCL2
GO:0045321	leukocyte activation	23/119	PSMC2 KPNB1 JUN STAT3 FGL2 TGFB1 SMAD3 CLEC4G CD209 SFTPD PPIA BCL2 ANPEP DPP4 CEACAM1 ANXA2 HSPD1 PSMD1 FKBP1A BAG6 PSMA2 EEF1A1 CAV1
GO:0050900	leukocyte migration	6/119	TGFB1 SFTPD PPIA CEACAM1 GBF1 CAV1
GO:0016049	cell growth	8/119	TWF2 PHB TGFB1 SMAD3 BCL2 CEACAM1 GSK3A GSK3B
GO:0040008	regulation of growth	11/119	TWF2 STAT5A PHB STAT3 TGFB1 SMAD3 BCL2L1 BCL2 CEACAM1 GSK3A GSK3B

ID	Description	GeneRatio	geneID
GO:0045926	negative regulation of growth	5/119	PHB TGFB1 SMAD3 BCL2 GSK3A
GO:0045927	positive regulation of growth	2/119	TWF2 BCL2
GO:0048589	developmental growth	10/119	TWF2 STAT5A STAT3 TGFB1 SMAD3 ATF5 BCL2 ANXA2 GSK3A GSK3B
GO:0002209	behavioral defense response	2/119	BCL2 DPP4
GO:0007611	learning or memory	1/119	JUN
GO:0007626	locomotory behavior	1/119	DPP4
GO:0007631	feeding behavior	2/119	STAT3 ACE2
GO:0035640	exploration behavior	1/119	DPP4
GO:0050795	regulation of behavior	1/119	STAT3
GO:0061744	motor behavior	1/119	DPP4
GO:0008284	positive regulation of cell proliferation	11/119	STAT5A JUN STAT3 TGFB1 CD209 BCL2L1 BCL2 SKP2 DPP4 ANXA2 NPM1
GO:0008285	negative regulation of cell proliferation	15/119	PHB JUN STAT3 TGFB1 SMAD3 ATF5 CLEC4G SFTPD BCL2 PRKRA CD9 CEACAM1 NPM1 HGS CAV1
GO:0033002	muscle cell proliferation	3/119	JUN STAT3 SKP2
GO:0033687	osteoblast proliferation	2/119	SMAD3 BCL2
GO:0035726	common myeloid progenitor cell proliferation	1/119	CEACAM1
GO:0042127	regulation of cell proliferation	23/119	STAT5A PHB JUN STAT3 TGFB1 SMAD3 ATF5 ACE2 CLEC4G CD209 SFTPD BCL2L1 BCL2 SKP2 PRKRA CD9 DPP4 CEACAM1 ANXA2 NPM1 HGS BAG6 CAV1
GO:0048144	fibroblast proliferation	3/119	JUN TGFB1 ANXA2
GO:0050673	epithelial cell proliferation	8/119	STAT5A JUN STAT3 TGFB1 SMAD3 BCL2L2 CEACAM1 CAV1
GO:0061351	neural precursor cell proliferation	2/119	TGFB1 ATF5
GO:0070661	leukocyte proliferation	6/119	TGFB1 CLEC4G CD209 SFTPD BCL2 HSPD1
GO:0072089	stem cell proliferation	1/119	TGFB1
GO:0007059	chromosome segregation	6/119	KPNB1 CHMP4B BAG6 CHMP2B TERF1 UBE2I
GO:0007017	microtubule-based process	11/119	KPNB1 XPO1 ATF5 CHMP4B KIF11 GSK3B NPM1 MARK3 CHMP2B CHEK2 DCTN2
GO:0000075	cell cycle checkpoint	3/119	TGFB1 BCL2L1 CHEK2
GO:0000920	septum digestion after cytokinesis	1/119	CHMP2B
GO:0001775	cell activation	25/119	PSMC2 KPNB1 JUN STAT3 FGL2 TGFB1 SMAD3 CLEC4G CD209 SFTPD PPIA BCL2 CD9 ANPEP DPP4 CEACAM1 ANXA2 HSPD1 GBF1 PSMD1 FKBP1A BAG6 PSMA2 EEF1A1 CAV1
GO:0006457	protein folding	9/119	PPIA DNAJB1 HSPD1 HSPA9 FKBP1A PPIG PPIH PFDN5 SGTA

ID	Description	GeneRatio	geneID
GO:0007165	signal transduction	53/119	KPNB1 STAT5A PHB JUN STAT3 PPP1CA TGFB1 SMAD3 HNRNPA1 CD209 CLEC4M IRF3 KPNA2 SFTPD PPIA BCL2L1 BCL2L2 MCL1 BCL2A1 BCL2 SKP2 PRKRA CD9 IKBKB PTBP1 CEACAM1 ANXA2 HNRNPA2B1 HSPD1 HSPA9 NMT1 GBF1 NONO GSK3A GSK3B NPM1 G3BP1 G3BP2 PARP1 DDX1 FKBP1A MARK3 RCAN3 HGS BAG6 DDAH2 CAMLG PFDN5 CHEK2 DDX5 CAV1 UBE2I ATP6V1G1
GO:0006903	vesicle targeting	4/119	SCFD1 YKT6 STX5 GBF1
GO:0006928	movement of cell or subcellular component	15/119	KPNB1 STAT5A JUN STAT3 TGFB1 SMAD3 SFTPD PPIA BCL2 CD9 DPP4 CEACAM1 GBF1 KIF11 CAV1
GO:0007049	cell cycle	24/119	KPNB1 JUN STAT3 PPP1CA XPO1 TGFB1 SMAD3 ATF5 BCL2L1 BCL2 SKP2 CHMP4B GBF1 RRM2 KIF11 SNX9 NPM1 RYBP BAG6 CHMP2B CHEK2 TERF1 DCTN2 UBE2I
GO:0007154	cell communication	55/119	KPNB1 STAT5A PHB JUN STAT3 PPP1CA TGFB1 SMAD3 HNRNPA1 CD209 CLEC4M IRF3 KPNA2 SFTPD PPIA BCL2L1 BCL2L2 MCL1 BCL2A1 BCL2 SKP2 PRKRA CD9 IKBKB DPP4 PTBP1 CEACAM1 ANXA2 HNRNPA2B1 HSPD1 HSPA9 NMT1 GBF1 NONO GSK3A GSK3B NPM1 G3BP1 G3BP2 PARP1 DDX1 FKBP1A MARK3 RCAN3 HGS BAG6 DDAH2 CAMLG SNAP47 PFDN5 CHEK2 DDX5 CAV1 UBE2I ATP6V1G1
GO:0007163	establishment or maintenance of cell polarity	3/119	GBF1 GSK3B MARK3
GO:0007272	ensheathment of neurons	2/119	TGFB1 CD9
GO:0008219	cell death	33/119	KPNB1 PHB JUN STAT3 PPP1CA TGFB1 SMAD3 ATF5 COX2 IRF3 BCL2L1 BCL2L2 MCL1 BCL2A1 BCL2 SKP2 PRKRA IKBKB HSPD1 HSPA9 NMT1 CHMP4B NONO GSK3A GSK3B NPM1 PARP1 RYBP BAG6 DDAH2 CHEK2 DDX5 CAV1
GO:0008037	cell recognition	2/119	CD209 CLEC4M
GO:0016043	cellular component organization	66/119	SCFD1 PSMC2 TFEB TWF2 YKT6 KPNB1 STX5 RSL24D1 PHB JUN STAT3 PPP1CA SPECC1 XPO1 TGFB1 SMAD3 ATF5 HNRNPA1 ACE2 SFTPD PPIA BCL2L1 BCL2 CD9 IKBKB DPP4 CEACAM1 ANXA2 HNRNPA2B1 DNAJB1 HSPD1 HSPA9 RPL13A EIF3E EIF3I NMT1 CHMP4B EIF3F GBF1 KIF11 SRP54 SNX9 GSK3A GSK3B NPM1 G3BP1 G3BP2 PARP1 DDX1 RYBP FKBP1A MARK3 PPIH HGS BAG6 CHMP2B SNAP47 TPSAB1 ABHD17A NDUFA10 H2AFY2 CHEK2 TERF1 DCTN2 CAV1 SGTA
GO:0016458	gene silencing	7/119	STAT3 TGFB1 SMAD3 PRKRA HNRNPA2B1 PABPC1 DDX5
GO:0019725	cellular homeostasis	10/119	TGFB1 SMAD3 MCL1 BCL2 ANXA2 FKBP1A CHMP2B TERF1 CAV1 ATP6V1G1
GO:0022402	cell cycle process	19/119	KPNB1 XPO1 TGFB1 SMAD3 ATF5 BCL2L1 BCL2 SKP2 CHMP4B RRM2 KIF11 SNX9 NPM1 RYBP BAG6 CHMP2B CHEK2 TERF1 DCTN2
GO:0022406	membrane docking	4/119	SCFD1 YKT6 STX5 DCTN2
GO:0022412	cellular process involved in reproduction in multicellular organism	4/119	TGFB1 BCL2L1 BCL2 CD9
GO:0030029	actin filament-based process	7/119	TWF2 SPECC1 TGFB1 SMAD3 BCL2 IKBKB CAV1
GO:0032940	secretion by cell	21/119	SCFD1 PSMC2 YKT6 KPNB1 FGL2 TGFB1 IRF3 PPIA CD9 ANPEP DPP4 CEACAM1 ANXA2 HSPD1 PSMD1 GSK3B HGS SNAP47 SERPING1 PSMA2 EEF1A1
GO:0033059	cellular pigmentation	2/119	BCL2 DCTN2
GO:0044764	multi-organism cellular process	2/119	IRF3 PPIA
GO:0048522	positive regulation of cellular process	54/119	PSMC2 TFEB TWF2 STAT5A PHB JUN STAT3 PPP1CA TGFB1 SMAD3 ATF5 COX2 HNRNPA1 ACE2 CD209 IRF3 SFTPD PPIA BCL2L1 MCL1 BCL2 SKP2 PRKRA IKBKB DPP4 PTBP1 CEACAM1 ANXA2 HNRNPA2B1 HSPD1 EIF3E NMT1 SNX9 GSK3A GSK3B PABPC1 NPM1 PARP1 NCL DDX1 RYBP FKBP1A HGS BAG6 DDAH2 SNAP47 MIF4GD H2AFY2 CHEK2 TERF1 DDX5 CAV1 UBE2I SGTA

ID	Description	GeneRatio	geneID
GO:0048523	negative regulation of cellular process	57/119	SCFD1 TWF2 PHB JUN STAT3 XPO1 TGFB1 SMAD3 ATF5 HNRNPA1 CLEC4G IRF3 SFTPD BCL2L1 BCL2L2 MCL1 BCL2A1 BCL2 PRKRA CD9 IKBKB DPP4 SYNCRIP PTBP1 CEACAM1 ANXA2 HNRNPA2B1 DNAJB1 HSPD1 HSPA9 RPL13A EIF3E CHMP4B RRM2 NONO GSK3A GSK3B PABPC1 NPM1 G3BP1 PARP1 NCL RYBP FKBP1A MARK3 HGS BAG6 DDAH2 SERPING1 PFDN5 H2AFY2 CHEK2 TERF1 DDX5 CAV1 UBE2I SGTA
GO:0048869	cellular developmental process	30/119	SCFD1 PSMC2 TWF2 PHB JUN STAT3 TGFB1 SMAD3 ATF5 BCL2L1 MCL1 BCL2 CD9 IKBKB ANPEP SYNCRIP PTBP1 CEACAM1 ANXA2 HSPA9 GSK3A GSK3B PABPC4 NPM1 PARP1 BAG6 H2AFY2 CHEK2 DDX5 CAV1
GO:0050794	regulation of cellular process	86/119	SCFD1 PSMC2 TFEB TWF2 KPNB1 STX5 STAT5A PHB JUN STAT3 PPP1CA XPO1 TGFB1 SMAD3 ATF5 COX2 HNRNPA1 ACE2 CLEC4G CD209 CLEC4M IRF3 KPNA2 SFTPD PPIA BCL2L1 BCL2L2 MCL1 BCL2A1 BCL2 SKP2 PRKRA CD9 IKBKB DPP4 SYNCRIP PTBP1 CEACAM1 ANXA2 HNRNPA2B1 DNAJB1 HSPD1 HSPA9 RPL13A EIF3E NMT1 CHMP4B GBF1 RRM2 KIF11 SNX9 NONO GSK3A GSK3B PABPC1 PABPC4 NPM1 G3BP1 G3BP2 PARP1 NCL DDX1 RYBP FKBP1A MARK3 RCAN3 HGS BAG6 DDAH2 CAMLG CHMP2B SNAP47 SERPING1 ABHD17A PFDN5 MIF4GD H2AFY2 CHEK2 TERF1 DCTN2 DDX5 EEF1A1 CAV1 UBE2I SGTA ATP6V1G1
GO:0051301	cell division	10/119	PPP1CA TGFB1 BCL2L1 CHMP4B KIF11 SNX9 CHMP2B CHEK2 TERF1 UBE2I
GO:0051651	maintenance of location in cell	2/119	TWF2 CAV1
GO:0051716	cellular response to stimulus	60/119	TWF2 KPNB1 STAT5A PHB JUN STAT3 PPP1CA TGFB1 SMAD3 HNRNPA1 CD209 CLEC4M IRF3 KPNA2 SFTPD PPIA BCL2L1 BCL2L2 MCL1 BCL2A1 BCL2 SKP2 PRKRA CD9 IKBKB SYNCRIP PTBP1 CEACAM1 ANXA2 HNRNPA2B1 DNAJB1 HSPD1 HSPA9 RPL13A NMT1 GBF1 NONO GSK3A GSK3B NPM1 G3BP1 G3BP2 PARP1 NCL DDX1 FKBP1A MARK3 RCAN3 HGS BAG6 DDAH2 CAMLG PFDN5 CHEK2 DDX5 EEF1A1 CAV1 UBE2I SGTA ATP6V1G1
GO:0060352	cell adhesion molecule production	1/119	CAV1
GO:0061919	process utilizing autophagic mechanism	11/119	SCFD1 TFEB ACBD5 MCL1 BCL2 CHMP4B GSK3A HGS CHMP2B EEF1A1 ATP6V1G1
GO:0071804	cellular potassium ion transport	1/119	CAV1
GO:0097194	execution phase of apoptosis	2/119	KPNB1 BCL2L1
GO:0140029	exocytic process	3/119	SCFD1 YKT6 SNAP47
GO:0006808	regulation of nitrogen utilization	1/119	BCL2
GO:0003006	developmental process involved in reproduction	6/119	TFEB PHB TGFB1 BCL2L1 BCL2L2 BCL2
GO:0009566	fertilization	2/119	BCL2L1 CD9
GO:0044703	multi-organism reproductive process	7/119	STAT3 TGFB1 BCL2L1 BCL2L2 BCL2 CD9 BAG6
GO:0048609	multicellular organismal reproductive process	5/119	TGFB1 BCL2L1 BCL2L2 BCL2 BAG6
GO:0051321	meiotic cell cycle	2/119	BAG6 TERF1
GO:0060011	Sertoli cell proliferation	1/119	BCL2L2
GO:0090220	chromosome localization to nuclear envelope involved in homologous chromosome segregation	1/119	TERF1
GO:1903046	meiotic cell cycle process	2/119	BAG6 TERF1

ID	Description	GeneRatio	geneID
GO:0007155	cell adhesion	15/119	PPP1CA TGFB1 SMAD3 CLEC4G CD209 CLEC4M SFTPD BCL2 CD9 DPP4 CEACAM1 ANXA2 HSPD1 GSK3B CAV1
GO:0044406	adhesion of symbiont to host	4/119	ACE2 CD209 CLEC4M SFTPD
GO:0007267	cell-cell signaling	11/119	STAT3 PPP1CA SMAD3 KPNA2 DPP4 GSK3A GSK3B G3BP1 SNAP47 PFDN5 CAV1
GO:0023051	regulation of signaling	39/119	PHB JUN STAT3 PPP1CA TGFB1 SMAD3 ACE2 IRF3 BCL2L1 BCL2L2 MCL1 BCL2A1 BCL2 SKP2 PRKRA IKBKB DPP4 PTBP1 CEACAM1 ANXA2 NMT1 GBF1 NONO GSK3A GSK3B G3BP1 PARP1 DDX1 FKBP1A MARK3 RCAN3 HGS CAMLG SNAP47 PFDN5 CHEK2 DDX5 CAV1 UBE2I
GO:0023056	positive regulation of signaling	25/119	PHB JUN STAT3 PPP1CA TGFB1 SMAD3 IRF3 BCL2L1 MCL1 BCL2 SKP2 PRKRA IKBKB PTBP1 ANXA2 NMT1 GSK3A GSK3B PARP1 DDX1 FKBP1A SNAP47 DDX5 CAV1 UBE2I
GO:0023057	negative regulation of signaling	17/119	PHB TGFB1 SMAD3 BCL2L1 BCL2L2 MCL1 BCL2A1 BCL2 CEACAM1 NONO GSK3A GSK3B G3BP1 MARK3 HGS PFDN5 CAV1
GO:0035637	multicellular organismal signaling	2/119	ACE2 CAV1
GO:0001503	ossification	7/119	PSMC2 PHB TGFB1 SMAD3 BCL2 SYNCRIP DDX5
GO:0001763	morphogenesis of a branching structure	3/119	PPP1CA TGFB1 BCL2
GO:0001816	cytokine production	8/119	TGFB1 SMAD3 ACE2 IRF3 SFTPD CEACAM1 HSPD1 DDX1
GO:0003008	system process	12/119	JUN TGFB1 SMAD3 ACE2 BCL2 CEACAM1 GSK3A GSK3B PARP1 CHMP2B SERPING1 CAV1
GO:0007275	multicellular organism development	44/119	TFEB TWF2 STAT5A PHB JUN STAT3 PPP1CA TGFB1 SMAD3 ATF5 COX2 SFTPD BCL2L1 BCL2L2 MCL1 BCL2A1 BCL2 PRKRA CD9 IKBKB ANPEP CEACAM1 ANXA2 ACO2 DNAJB1 HSPD1 HSPA9 NMT1 GSK3A GSK3B PABPC4 RPL19 PARP1 NCL DDX1 RYBP FKBP1A HGS BAG6 PFDN5 VKORC1 H2AFY2 DDX5 CAV1
GO:0007389	pattern specification process	1/119	SMAD3
GO:0007585	respiratory gaseous exchange	1/119	SFTPD
GO:0009791	post-embryonic development	2/119	ATF5 BCL2
GO:0019827	stem cell population maintenance	1/119	STAT3
GO:0022404	molting cycle process	1/119	BCL2
GO:0032922	circadian regulation of gene expression	1/119	PPP1CA
GO:0033555	multicellular organismal response to stress	2/119	BCL2 DPP4
GO:0034381	plasma lipoprotein particle clearance	1/119	ANXA2
GO:0035264	multicellular organism growth	4/119	STAT5A STAT3 ATF5 BCL2
GO:0035265	organ growth	3/119	BCL2 ANXA2 GSK3A
GO:0042303	molting cycle	1/119	BCL2
GO:0044706	multi-multicellular organism process	2/119	TGFB1 BCL2

ID	Description	GeneRatio	geneID
GO:0048771	tissue remodeling	3/119	TGFB1 CEACAM1 CAV1
GO:0048871	multicellular organismal homeostasis	5/119	STAT3 SFTPD BCL2 CHMP4B CAV1
GO:0050817	coagulation	7/119	CD9 CEACAM1 ANXA2 PABPC4 SERPING1 VKORC1 CAV1
GO:0051239	regulation of multicellular organismal process	27/119	TWF2 STAT5A JUN STAT3 TGFB1 SMAD3 ACE2 IRF3 SFTPD BCL2 CD9 IKBKB CEACAM1 ANXA2 HSPD1 HSPA9 GSK3A GSK3B PARP1 DDX1 HGS BAG6 SERPING1 VKORC1 H2AFY2 DDX5 CAV1
GO:0051240	positive regulation of multicellular organismal process	17/119	TWF2 STAT5A JUN STAT3 TGFB1 SMAD3 ACE2 IRF3 BCL2 CEACAM1 ANXA2 HSPD1 GSK3A PARP1 DDX1 H2AFY2 CAV1
GO:0051241	negative regulation of multicellular organismal process	14/119	STAT3 TGFB1 SMAD3 IRF3 SFTPD BCL2 CD9 CEACAM1 ANXA2 HSPA9 GSK3A GSK3B HGS SERPING1
GO:0090130	tissue migration	5/119	STAT5A JUN TGFB1 DPP4 CEACAM1
GO:0009653	anatomical structure morphogenesis	21/119	SCFD1 TWF2 JUN STAT3 PPP1CA TGFB1 SMAD3 BCL2L1 BCL2 PRKRA CD9 ANPEP CEACAM1 ANXA2 GSK3A GSK3B NCL FKBP1A RCAN3 HGS CAV1
GO:0007568	aging	8/119	JUN STAT3 TGFB1 BCL2A1 BCL2 NPM1 SERPING1 CHEK2
GO:0048646	anatomical structure formation involved in morphogenesis	12/119	JUN STAT3 TGFB1 SMAD3 CD9 ANPEP CEACAM1 ANXA2 NCL FKBP1A HGS CAV1
GO:0021700	developmental maturation	1/119	BCL2
GO:0044111	development involved in symbiotic interaction	1/119	ANXA2
GO:0048856	anatomical structure development	47/119	SCFD1 TFEB TWF2 STAT5A PHB JUN STAT3 PPP1CA TGFB1 SMAD3 ATF5 COX2 SFTPD BCL2L1 BCL2L2 MCL1 BCL2A1 BCL2 PRKRA CD9 IKBKB ANPEP PTBP1 CEACAM1 ANXA2 ACO2 DNAJB1 HSPD1 HSPA9 NMT1 GSK3A GSK3B PABPC4 RPL19 PARP1 NCL DDX1 RYBP FKBP1A RCAN3 HGS BAG6 PFDN5 VKORC1 H2AFY2 DDX5 CAV1
GO:0050793	regulation of developmental process	20/119	TWF2 STAT5A JUN STAT3 TGFB1 SMAD3 BCL2 IKBKB PTBP1 CEACAM1 ANXA2 HSPA9 GSK3A GSK3B PARP1 HGS BAG6 H2AFY2 DDX5 CAV1
GO:0051093	negative regulation of developmental process	12/119	STAT3 TGFB1 SMAD3 BCL2 PTBP1 CEACAM1 ANXA2 HSPA9 GSK3A GSK3B HGS CAV1
GO:0051094	positive regulation of developmental process	10/119	TWF2 JUN STAT3 TGFB1 SMAD3 BCL2 CEACAM1 ANXA2 PARP1 H2AFY2
GO:0098727	maintenance of cell number	1/119	STAT3
GO:0040012	regulation of locomotion	10/119	STAT5A JUN STAT3 TGFB1 SMAD3 BCL2 CEACAM1 CHMP4B CHMP2B CAV1
GO:0040013	negative regulation of locomotion	3/119	STAT3 TGFB1 BCL2
GO:0040017	positive regulation of locomotion	9/119	STAT5A JUN STAT3 TGFB1 SMAD3 BCL2 CHMP4B CHMP2B CAV1
GO:0042330	taxis	4/119	TGFB1 SMAD3 SFTPD GBF1
GO:0048870	cell motility	12/119	STAT5A JUN STAT3 TGFB1 SMAD3 SFTPD PPIA BCL2 DPP4 CEACAM1 GBF1 CAV1
GO:0052192	movement in environment of other organism involved in symbiotic interaction	3/119	PPIA CHMP4B CHMP2B

ID	Description	GeneRatio	geneID
GO:0048066	developmental pigmentation	1/119	BCL2
GO:0007623	circadian rhythm	5/119	JUN PPP1CA ATF5 NONO GSK3B
GO:0043902	positive regulation of multi-organism process	8/119	JUN PPIA TMPRSS2 ANXA2 CHMP4B PABPC1 PPIH CHMP2B
GO:0044089	positive regulation of cellular component biogenesis	12/119	PSMC2 TWF2 JUN TGFB1 SMAD3 ACE2 SNX9 GSK3B PARP1 HGS TERF1 CAV1
GO:0045785	positive regulation of cell adhesion	7/119	TGFB1 SMAD3 CD209 DPP4 HSPD1 GSK3B CAV1
GO:0048087	positive regulation of developmental pigmentation	1/119	BCL2
GO:0048584	positive regulation of response to stimulus	31/119	PHB JUN STAT3 PPP1CA TGFB1 SMAD3 CD209 IRF3 SFTPD BCL2L1 MCL1 BCL2 SKP2 PRKRA IKBKB PTBP1 CEACAM1 HSPD1 NMT1 NONO GSK3A GSK3B PARP1 DDX1 FKBP1A BAG6 SERPING1 DDX5 CAV1 UBE2I SGTA
GO:0051050	positive regulation of transport	14/119	TGFB1 SMAD3 ACE2 IRF3 SFTPD PPIA BCL2 ANXA2 HSPD1 NMT1 GSK3A GSK3B HGS CAV1
GO:0051091	positive regulation of DNA-binding transcription factor activity	5/119	TGFB1 SMAD3 IKBKB NPM1 UBE2I
GO:1903829	positive regulation of cellular protein localization	8/119	TGFB1 SMAD3 BCL2 NMT1 GSK3A GSK3B PARP1 ABHD17A
GO:1904181	positive regulation of membrane depolarization	1/119	PARP1
GO:1904951	positive regulation of establishment of protein localization	9/119	TGFB1 SMAD3 IRF3 PPIA BCL2 HSPD1 NMT1 GSK3A GSK3B
GO:1905954	positive regulation of lipid localization	1/119	ANXA2
GO:0043433	negative regulation of DNA-binding transcription factor activity	1/119	G3BP2
GO:0043901	negative regulation of multi-organism process	2/119	JUN ANXA2
GO:0048585	negative regulation of response to stimulus	23/119	PHB TGFB1 SMAD3 CLEC4G BCL2L1 BCL2L2 MCL1 BCL2A1 BCL2 CD9 CEACAM1 ANXA2 NONO GSK3A GSK3B G3BP1 MARK3 HGS SERPING1 PFDN5 CHEK2 CAV1 SGTA
GO:0051051	negative regulation of transport	5/119	TGFB1 BCL2 CEACAM1 GSK3A CAV1
GO:1900047	negative regulation of hemostasis	4/119	CD9 CEACAM1 ANXA2 SERPING1
GO:1903828	negative regulation of cellular protein localization	5/119	TGFB1 BCL2L1 GSK3B ABHD17A TERF1
GO:1904180	negative regulation of membrane depolarization	1/119	BCL2
GO:1904911	negative regulation of establishment of RNA localization to telomere	1/119	TERF1

ID	Description	GeneRatio	geneID
GO:1904914	negative regulation of establishment of protein-containing complex localization to telomere	1/119	TERF1
GO:1904950	negative regulation of establishment of protein localization	1/119	TERF1
GO:0030155	regulation of cell adhesion	12/119	TGFB1 SMAD3 CLEC4G CD209 SFTPD BCL2 CD9 DPP4 CEACAM1 HSPD1 GSK3B CAV1
GO:0032879	regulation of localization	28/119	SCFD1 STAT5A JUN STAT3 XPO1 TGFB1 SMAD3 ACE2 IRF3 SFTPD PPIA BCL2L1 BCL2 CD9 DPP4 CEACAM1 ANXA2 HSPD1 NMT1 GBF1 GSK3A GSK3B PARP1 FKBP1A HGS ABHD17A TERF1 CAV1
GO:0042752	regulation of circadian rhythm	2/119	PPP1CA NONO
GO:0043900	regulation of multi-organism process	12/119	JUN SFTPD PPIA BCL2 TMPRSS2 ANXA2 CHMP4B PABPC1 PPIH CHMP2B DDX5 CAV1
GO:0044087	regulation of cellular component biogenesis	19/119	SCFD1 PSMC2 TWF2 JUN TGFB1 SMAD3 ACE2 IKBKB DNAJB1 RPL13A CHMP4B SNX9 GSK3B NPM1 PARP1 HGS CHMP2B TERF1 CAV1
GO:0048070	regulation of developmental pigmentation	1/119	BCL2
GO:0048518	positive regulation of biological process	63/119	PSMC2 TFEB TWF2 STX5 STAT5A PHB JUN STAT3 PPP1CA TGFB1 SMAD3 ATF5 COX2 HNRNPA1 ACE2 CD209 IRF3 SFTPD PPIA BCL2L1 MCL1 BCL2 SKP2 PRKRA TMPRSS2 IKBKB DPP4 SYNCRIP PTBP1 CEACAM1 ANXA2 HNRNPA2B1 HSPD1 EIF3E NMT1 CHMP4B SNX9 NONO GSK3A GSK3B PABPC1 NPM1 PARP1 NCL DDX1 RYBP FKBP1A PPIH HGS BAG6 DDAH2 CHMP2B SNAP47 SERPING1 ABHD17A MIF4GD H2AFY2 CHEK2 TERF1 DDX5 CAV1 UBE2I SGTA
GO:0048519	negative regulation of biological process	62/119	SCFD1 TWF2 PHB JUN STAT3 XPO1 TGFB1 SMAD3 ATF5 HNRNPA1 CLEC4G IRF3 SFTPD BCL2L1 BCL2L2 MCL1 BCL2A1 BCL2 PRKRA CD9 IKBKB DPP4 SYNCRIP PTBP1 CEACAM1 ANXA2 HNRNPA2B1 DNAJB1 HSPD1 HSPA9 RPL13A EIF3E CHMP4B RRM2 NONO GSK3A GSK3B PABPC1 PABPC4 NPM1 G3BP1 G3BP2 RPL19 PARP1 NCL RYBP FKBP1A MARK3 HGS BAG6 DDAH2 SERPING1 ABHD17A PFDN5 H2AFY2 RPS20 CHEK2 TERF1 DDX5 CAV1 UBE2I SGTA
GO:0048583	regulation of response to stimulus	47/119	PHB JUN STAT3 PPP1CA TGFB1 SMAD3 ACE2 CLEC4G CD209 IRF3 SFTPD BCL2L1 BCL2L2 MCL1 BCL2A1 BCL2 SKP2 PRKRA CD9 IKBKB PTBP1 CEACAM1 ANXA2 DNAJB1 HSPD1 NMT1 GBF1 NONO GSK3A GSK3B NPM1 G3BP1 PARP1 DDX1 FKBP1A MARK3 RCAN3 HGS BAG6 SERPING1 PFDN5 VKORC1 CHEK2 DDX5 CAV1 UBE2I SGTA
GO:0097006	regulation of plasma lipoprotein particle levels	1/119	ANXA2
GO:0098900	regulation of action potential	1/119	CAV1
GO:1900046	regulation of hemostasis	6/119	CD9 CEACAM1 ANXA2 SERPING1 VKORC1 CAV1
GO:0006950	response to stress	49/119	SCFD1 PHB JUN STAT3 PPP1CA TGFB1 SMAD3 COX2 HNRNPA1 ACE2 CD209 CLEC4M IRF3 SFTPD BCL2L1 BCL2L2 MCL1 BCL2A1 BCL2 SKP2 KPNA4 PRKRA CD9 IKBKB DPP4 SYNCRIP CEACAM1 ANXA2 ACO2 DNAJB1 HSPD1 HSPA9 RPL13A NONO GSK3A GSK3B PABPC4 NPM1 PARP1 DDX1 BAG6 CAMLG TPSAB1 SERPING1 VKORC1 CHEK2 DDX5 CAV1 SGTA
GO:0009605	response to external stimulus	25/119	PHB JUN PPP1CA TGFB1 SMAD3 HNRNPA1 ACE2 IRF3 SFTPD BCL2L1 BCL2 SKP2 PRKRA CD9 IKBKB CEACAM1 ANXA2 HSPD1 NMT1 GBF1 DDX1 SERPING1 PSMA2 VKORC1 CAV1
GO:0009607	response to biotic stimulus	17/119	JUN TGFB1 SMAD3 IRF3 SFTPD BCL2L1 BCL2 SKP2 PRKRA IKBKB CEACAM1 HSPD1 GBF1 GSK3B DDX1 PSMA2 CAV1
GO:0009628	response to abiotic stimulus	20/119	SCFD1 JUN PPP1CA TGFB1 SMAD3 COX2 BCL2L1 BCL2 CD9 DPP4 DNAJB1 HSPD1 HSPA9 NMT1 GSK3B NPM1 PARP1 CHEK2 CAV1 ATP6V1G1

ID	Description	GeneRatio	geneID
GO:0009719	response to endogenous stimulus	25/119	STAT5A PHB JUN STAT3 TGFB1 SMAD3 HNRNPA1 BCL2L1 BCL2 SKP2 CD9 PTBP1 CEACAM1 ANXA2 HSPD1 GSK3A GSK3B PARP1 NCL CHEK2 DDX5 EEF1A1 CAV1 UBE2I ATP6V1G1
GO:0014823	response to activity	1/119	HSPD1
GO:0042221	response to chemical	51/119	SCFD1 TWF2 STAT5A PHB JUN STAT3 PPP1CA XPO1 TGFB1 SMAD3 HNRNPA1 IRF3 SFTPD PPIA BCL2L1 MCL1 BCL2 SKP2 KPNA4 PRKRA CD9 IKBKB SYNCRIP PTBP1 CEACAM1 ANXA2 HNRNPA2B1 DNAJB1 HSPD1 HSPA9 RPL13A GBF1 SRP54 NONO GSK3A GSK3B NPM1 PARP1 NCL DDX1 HGS BAG6 VKORC1 CHEK2 TERF1 DDX5 EEF1A1 CAV1 UBE2I SGTA ATP6V1G1
GO:0043500	muscle adaptation	3/119	SMAD3 GSK3A PARP1
GO:0043627	response to estrogen	2/119	HSPD1 CAV1
GO:0051606	detection of stimulus	2/119	NMT1 PARP1
GO:0072376	protein activation cascade	2/119	PHB SERPING1
GO:0031503	protein-containing complex localization	5/119	XPO1 HNRNPA2B1 NPM1 SNAP47 TERF1
GO:0033036	macromolecule localization	52/119	BTF3 SCFD1 TWF2 YKT6 KPNB1 STX5 STAT3 XPO1 TGFB1 SMAD3 HNRNPA1 IRF3 KPNA2 PPIA BCL2L1 MCL1 BCL2 KPNA4 IKBKB DPP4 CEACAM1 ANXA2 HNRNPA2B1 HNRNPA3 HSPD1 COPB2 RPL13A NMT1 CHMP4B GBF1 SRP54 SNX9 GSK3A GSK3B NPM1 G3BP2 RPL19 PARP1 DDX1 FKBP1A HGS BAG6 CHMP2B SNAP47 ABHD17A H2AFY2 RPS20 TERF1 DCTN2 CAV1 SGTA ATP6V1G1
GO:0051234	establishment of localization	64/119	BTF3 SCFD1 PSMC2 YKT6 KPNB1 STX5 STAT5A PHB STAT3 FGL2 XPO1 TGFB1 SMAD3 COX2 HNRNPA1 ACE2 CD209 CLEC4M IRF3 KPNA2 SFTPD PPIA BCL2L1 MCL1 BCL2 KPNA4 CD9 TMPRSS2 ANPEP DPP4 CEACAM1 ANXA2 HNRNPA2B1 HNRNPA3 HSPD1 COPB2 RPL13A NMT1 CHMP4B GBF1 KIF11 PSMD1 SRP54 SNX9 GSK3A GSK3B NPM1 G3BP2 RPL19 FKBP1A HGS BAG6 CHMP2B SNAP47 SERPING1 PSMA2 H2AFY2 RPS20 TERF1 DCTN2 EEF1A1 CAV1 SGTA ATP6V1G1
GO:0051235	maintenance of location	5/119	TWF2 TGFB1 G3BP2 FKBP1A CAV1
GO:0051641	cellular localization	43/119	SCFD1 TWF2 YKT6 KPNB1 STX5 STAT3 XPO1 TGFB1 SMAD3 HNRNPA1 KPNA2 BCL2L1 BCL2 KPNA4 IKBKB ANXA2 HNRNPA2B1 HSPD1 COPB2 RPL13A NMT1 CHMP4B GBF1 SRP54 SNX9 GSK3A GSK3B NPM1 RPL19 PARP1 DDX1 FKBP1A HGS BAG6 CHMP2B SNAP47 ABHD17A H2AFY2 RPS20 TERF1 DCTN2 CAV1 SGTA
GO:0051674	localization of cell	12/119	STAT5A JUN STAT3 TGFB1 SMAD3 SFTPD PPIA BCL2 DPP4 CEACAM1 GBF1 CAV1
GO:1902579	multi-organism localization	5/119	KPNB1 XPO1 CD209 CLEC4M CAV1
GO:0051707	response to other organism	15/119	JUN TGFB1 SMAD3 IRF3 SFTPD BCL2L1 BCL2 SKP2 PRKRA IKBKB HSPD1 GBF1 DDX1 PSMA2 CAV1
GO:0044419	interspecies interaction between organisms	38/119	KPNB1 JUN STAT3 XPO1 TGFB1 SMAD3 HNRNPA1 ACE2 CLEC4G CD209 CLEC4M IRF3 KPNA2 SFTPD PPIA BCL2L1 BCL2 KPNA4 TMPRSS2 IKBKB ANPEP DPP4 SYNCRIP PTBP1 ANXA2 HSPD1 CHMP4B EIF3F GBF1 PABPC1 NPM1 PPIH CAMLG CHMP2B DDX5 CAV1 UBE2I SGTA
GO:0050789	regulation of biological process	92/119	SCFD1 PSMC2 TFEB TWF2 KPNB1 STX5 STAT5A PHB JUN STAT3 PPP1CA XPO1 TGFB1 SMAD3 ATF5 COX2 HNRNPA1 ACE2 CLEC4G CD209 CLEC4M IRF3 KPNA2 SFTPD PPIA BCL2L1 BCL2L2 MCL1 BCL2A1 BCL2 SKP2 PRKRA CD9 TMPRSS2 IKBKB DPP4 SYNCRIP PTBP1 CEACAM1 ANXA2 HNRNPA2B1 DNAJB1 HSPD1 HSPA9 RPL13A EIF3E NMT1 CHMP4B GBF1 RRM2 KIF11 PSMD1 SNX9 NONO GSK3A GSK3B PABPC1 PABPC4 NPM1 G3BP1 G3BP2 RPL19 PARP1 NCL DDX1 RYBP FKBP1A MARK3 PPIH RCAN3 HGS BAG6 DDAH2 CAMLG CHMP2B SNAP47 SERPING1 ABHD17A PFDN5 MIF4GD VKORC1 H2AFY2 RPS20 CHEK2 TERF1 DCTN2 DDX5 EEF1A1 CAV1 UBE2I SGTA ATP6V1G1
GO:0065008	regulation of biological quality	46/119	TWF2 STAT5A PHB JUN STAT3 PPP1CA XPO1 TGFB1 SMAD3 COX2 HNRNPA1 ACE2 SFTPD BCL2L1 MCL1 BCL2 PRKRA CD9 DPP4 SYNCRIP CEACAM1 ANXA2 HNRNPA2B1 HSPD1 HSPA9 NMT1 CHMP4B GSK3A GSK3B PABPC1 PABPC4

ID	Description	GeneRatio	geneID
			NPM1 G3BP2 PARP1 FKBP1A BAG6 DDAH2 CHMP2B SNAP47 SERPING1 ABHD17A VKORC1 CHEK2 TERF1 CAV1 ATP6V1G1
GO:0065009	regulation of molecular function	31/119	JUN STAT3 PPP1CA TGFB1 SMAD3 SFTPD BCL2 PRKRA IKBKB CEACAM1 ANXA2 HNRNPA2B1 DNAJB1 HSPD1 EIF3E PSMD1 SNX9 GSK3A GSK3B NPM1 G3BP2 PARP1 FKBP1A MARK3 RCAN3 HGS DDAH2 SERPING1 TERF1 CAV1 UBE2I
GO:0044085	cellular component biogenesis	45/119	SCFD1 PSMC2 TWF2 KPNB1 STX5 RSL24D1 JUN XPO1 TGFB1 SMAD3 ACE2 BCL2 CD9 IKBKB ANXA2 DNAJB1 HSPD1 HSPA9 RPL13A EIF3E EIF3I CHMP4B EIF3F KIF11 SRP54 SNX9 GSK3B NPM1 G3BP1 G3BP2 PARP1 DDX1 FKBP1A PPIH HGS BAG6 CHMP2B SNAP47 NDUFA10 LAS1L H2AFY2 CHEK2 TERF1 DCTN2 CAV1

2 HCOV-CLUSTER_SET

2.1 GO CLASSIFICATION

Functional Profile of a gene set at specific GO level. Genes are classified according to their GO distribution at a specific level.

MOLECULAR FUNCTION

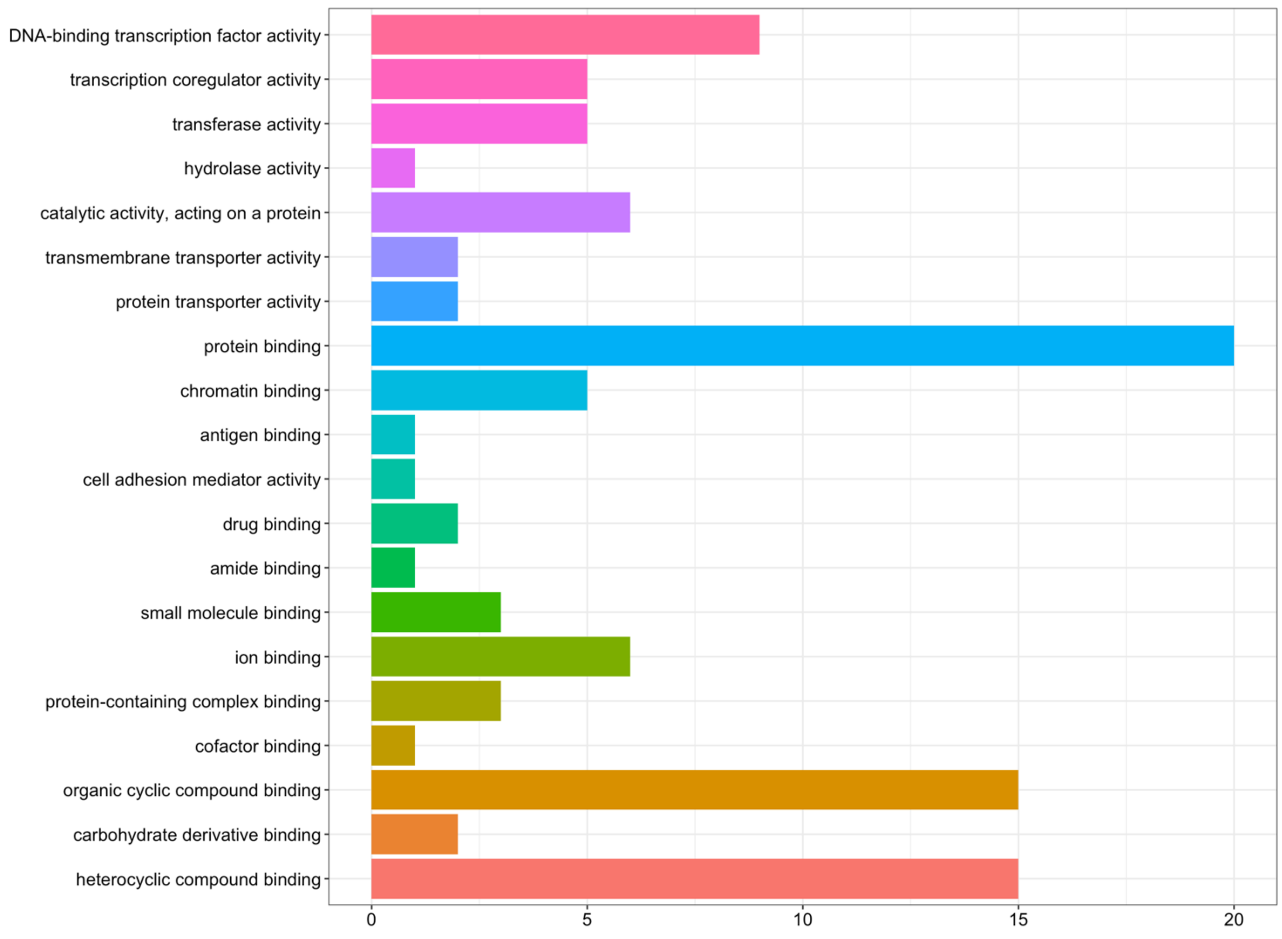

CELLULAR COMPONENT

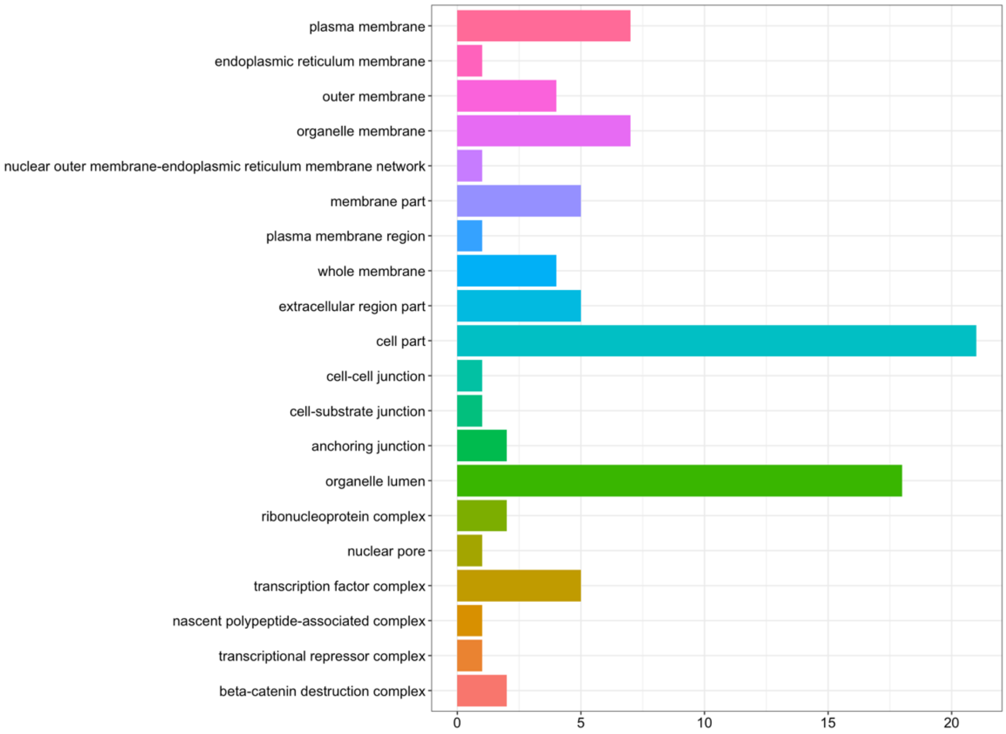

BIOLOGICAL PROCESS

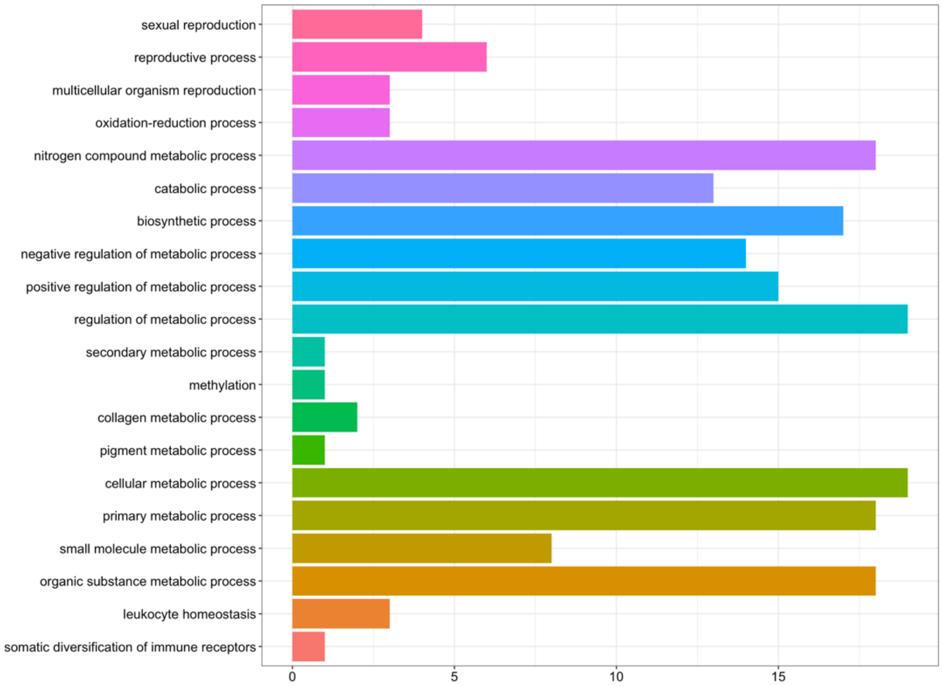

2.2 TABLES CLASSIFICATION

2.2.1 MOLECULAR FUNCTION

ID	Description	GeneRatio	geneID
GO:0003700	DNA-binding transcription factor activity	9/21	TFEB STAT5A PHB JUN STAT3 SMAD3 ATF5 IRF3 PARP1
GO:0003712	transcription coregulator activity	5/21	JUN SMAD3 ATF5 IRF3 NPM1
GO:0016740	transferase activity	5/21	STAT5A SKP2 GSK3A GSK3B PARP1
GO:0016787	hydrolase activity	1/21	PPP1CA
GO:0140096	catalytic activity, acting on a protein	6/21	STAT5A PPP1CA SKP2 GSK3A GSK3B PARP1
GO:0022857	transmembrane transporter activity	2/21	MCL1 BCL2
GO:0008565	protein transporter activity	2/21	KPNB1 MCL1
GO:0005515	protein binding	20/21	TFEB KPNB1 STAT5A PHB JUN STAT3 PPP1CA TGFB1 SMAD3 ATF5 IRF3 BCL2L1 MCL1 BCL2 SKP2 GSK3A GSK3B NPM1 PARP1 IL6
GO:0003682	chromatin binding	5/21	JUN STAT3 SMAD3 ATF5 NPM1
GO:0003823	antigen binding	1/21	TGFB1
GO:0098631	cell adhesion mediator activity	1/21	PPP1CA
GO:0008144	drug binding	2/21	GSK3A GSK3B
GO:0033218	amide binding	1/21	KPNB1
GO:0036094	small molecule binding	3/21	GSK3A GSK3B PARP1
GO:0043167	ion binding	6/21	KPNB1 PPP1CA SMAD3 GSK3A GSK3B PARP1
GO:0044877	protein-containing complex binding	3/21	PPP1CA SMAD3 NPM1
GO:0048037	cofactor binding	1/21	PARP1
GO:0097159	organic cyclic compound binding	15/21	TFEB KPNB1 STAT5A PHB JUN STAT3 SMAD3 ATF5 IRF3 BCL2 NACA GSK3A GSK3B NPM1 PARP1
GO:0097367	carbohydrate derivative binding	2/21	GSK3A GSK3B
GO:1901363	heterocyclic compound binding	15/21	TFEB KPNB1 STAT5A PHB JUN STAT3 SMAD3 ATF5 IRF3 BCL2 NACA GSK3A GSK3B NPM1 PARP1
GO:0016247	channel regulator activity	1/21	BCL2
GO:0030234	enzyme regulator activity	3/21	JUN TGFB1 NPM1
GO:0030545	receptor regulator activity	2/21	TGFB1 IL6
GO:0005072	transforming growth factor beta receptor, cytoplasmic mediator activity	1/21	SMAD3

2.2.2 CELLULAR COMPONENT

ID	Description	GeneRatio	geneID
GO:0005886	plasma membrane	7/21	PHB STAT3 PPP1CA TGFB1 SMAD3 GSK3B IL6
GO:0005789	endoplasmic reticulum membrane	1/21	BCL2
GO:0019867	outer membrane	4/21	PHB BCL2L1 MCL1 BCL2
GO:0031090	organelle membrane	7/21	KPNB1 PHB STAT3 SMAD3 BCL2L1 MCL1 BCL2
GO:0042175	nuclear outer membrane-endoplasmic reticulum membrane network	1/21	BCL2
GO:0044425	membrane part	5/21	PHB BCL2L1 MCL1 BCL2 IL6
GO:0098590	plasma membrane region	1/21	PHB
GO:0098805	whole membrane	4/21	PHB BCL2L1 MCL1 BCL2
GO:0044421	extracellular region part	5/21	KPNB1 PHB PPP1CA TGFB1 IL6
GO:0044464	cell part	21/21	TFEB KPNB1 STAT5A PHB JUN STAT3 PPP1CA TGFB1 SMAD3 ATF5 IRF3 BCL2L1 MCL1 BCL2 SKP2 NACA GSK3A GSK3B NPM1 PARP1 IL6
GO:0005911	cell-cell junction	1/21	PPP1CA
GO:0030055	cell-substrate junction	1/21	NPM1
GO:0070161	anchoring junction	2/21	PPP1CA NPM1
GO:0043233	organelle lumen	18/21	KPNB1 STAT5A PHB JUN STAT3 PPP1CA TGFB1 SMAD3 ATF5 IRF3 BCL2L1 MCL1 BCL2 SKP2 GSK3B NPM1 PARP1 IL6
GO:1990904	ribonucleoprotein complex	2/21	KPNB1 NPM1
GO:0005643	nuclear pore	1/21	KPNB1
GO:0005667	transcription factor complex	5/21	JUN STAT3 SMAD3 ATF5 PARP1
GO:0005854	nascent polypeptide-associated complex	1/21	NACA
GO:0017053	transcriptional repressor complex	1/21	JUN
GO:0030877	beta-catenin destruction complex	2/21	GSK3A GSK3B
GO:0032993	protein-DNA complex	2/21	NPM1 PARP1
GO:0043235	receptor complex	2/21	SMAD3 IL6
GO:0071141	SMAD protein complex	1/21	SMAD3
GO:0097136	Bcl-2 family protein complex	2/21	BCL2L1 MCL1
GO:0098796	membrane protein complex	2/21	BCL2 IL6
GO:1902494	catalytic complex	2/21	PPP1CA SKP2
GO:1990909	Wnt signalosome	1/21	GSK3B

ID	Description	GeneRatio	geneID
GO:0043227	membrane-bounded organelle	21/21	TFEB KPNB1 STAT5A PHB JUN STAT3 PPP1CA TGFB1 SMAD3 ATF5 IRF3 BCL2L1 MCL1 BCL2 SKP2 NACA GSK3A GSK3B NPM1 PARP1 IL6
GO:0043228	non-membrane-bounded organelle	11/21	KPNB1 JUN STAT3 PPP1CA SMAD3 ATF5 BCL2L1 SKP2 GSK3B NPM1 PARP1
GO:0043229	intracellular organelle	21/21	TFEB KPNB1 STAT5A PHB JUN STAT3 PPP1CA TGFB1 SMAD3 ATF5 IRF3 BCL2L1 MCL1 BCL2 SKP2 NACA GSK3A GSK3B NPM1 PARP1 IL6
GO:0043230	extracellular organelle	3/21	KPNB1 PHB PPP1CA
GO:0044422	organelle part	18/21	KPNB1 STAT5A PHB JUN STAT3 PPP1CA TGFB1 SMAD3 ATF5 IRF3 BCL2L1 MCL1 BCL2 SKP2 GSK3B NPM1 PARP1 IL6
GO:0099572	postsynaptic specialization	2/21	PHB STAT3
GO:0018995	host	1/21	KPNB1
GO:0044217	other organism part	1/21	KPNB1
GO:0044216	other organism cell	1/21	KPNB1
GO:0005615	extracellular space	5/21	KPNB1 PHB PPP1CA TGFB1 IL6
GO:0031012	extracellular matrix	1/21	TGFB1
GO:0072562	blood microparticle	1/21	TGFB1
GO:0044446	intracellular organelle part	18/21	KPNB1 STAT5A PHB JUN STAT3 PPP1CA TGFB1 SMAD3 ATF5 IRF3 BCL2L1 MCL1 BCL2 SKP2 GSK3B NPM1 PARP1 IL6
GO:0019898	extrinsic component of membrane	1/21	PHB
GO:0031224	intrinsic component of membrane	5/21	PHB BCL2L1 MCL1 BCL2 IL6
GO:0044455	mitochondrial membrane part	1/21	PHB
GO:0044459	plasma membrane part	2/21	PHB IL6
GO:0008021	synaptic vesicle	1/21	BCL2L1
GO:0030672	synaptic vesicle membrane	1/21	BCL2L1
GO:0048786	presynaptic active zone	1/21	PHB
GO:0097060	synaptic membrane	1/21	PHB
GO:0098793	presynapse	3/21	PHB PPP1CA BCL2L1
GO:0098794	postsynapse	5/21	PHB STAT3 PPP1CA GSK3A GSK3B
GO:0099243	extrinsic component of synaptic membrane	1/21	PHB
GO:0005622	intracellular	21/21	TFEB KPNB1 STAT5A PHB JUN STAT3 PPP1CA TGFB1 SMAD3 ATF5 IRF3 BCL2L1 MCL1 BCL2 SKP2 NACA GSK3A GSK3B NPM1 PARP1 IL6
GO:0008287	protein serine/threonine phosphatase complex	1/21	PPP1CA
GO:0009986	cell surface	2/21	PHB TGFB1
GO:0012505	endomembrane system	8/21	KPNB1 PHB TGFB1 SMAD3 BCL2L1 BCL2 PARP1 IL6

ID	Description	GeneRatio	geneID
GO:0031975	envelope	8/21	KPNB1 PHB STAT3 SMAD3 BCL2L1 MCL1 BCL2 PARP1
GO:0042995	cell projection	4/21	PPP1CA TGFB1 GSK3A GSK3B
GO:0043209	myelin sheath	2/21	PHB BCL2
GO:0044297	cell body	3/21	PPP1CA TGFB1 GSK3A
GO:0044424	intracellular part	21/21	TFEB KPNB1 STAT5A PHB JUN STAT3 PPP1CA TGFB1 SMAD3 ATF5 IRF3 BCL2L1 MCL1 BCL2 SKP2 NACA GSK3A GSK3B NPM1 PARP1 IL6
GO:0044463	cell projection part	3/21	PPP1CA GSK3A GSK3B
GO:0071944	cell periphery	7/21	PHB STAT3 PPP1CA TGFB1 SMAD3 GSK3B IL6
GO:0097458	neuron part	7/21	PHB STAT3 PPP1CA TGFB1 BCL2L1 GSK3A GSK3B
GO:0044456	synapse part	6/21	PHB STAT3 PPP1CA BCL2L1 GSK3A GSK3B
GO:0098685	Schaffer collateral - CA1 synapse	1/21	STAT3
GO:0098978	glutamatergic synapse	4/21	PHB STAT3 PPP1CA GSK3B
GO:0098982	GABA-ergic synapse	1/21	PHB
GO:0098984	neuron to neuron synapse	2/21	PHB STAT3

2.2.3 BIOLOGICAL PROCESS

ID	Description	GeneRatio	geneID
GO:0019953	sexual reproduction	4/21	STAT3 TGFB1 BCL2L1 BCL2
GO:0022414	reproductive process	6/21	TFEB PHB STAT3 TGFB1 BCL2L1 BCL2
GO:0032504	multicellular organism reproduction	3/21	TGFB1 BCL2L1 BCL2
GO:0055114	oxidation-reduction process	3/21	PPP1CA GSK3A GSK3B
GO:0006807	nitrogen compound metabolic process	18/21	TFEB KPNB1 STAT5A PHB JUN STAT3 PPP1CA TGFB1 SMAD3 ATF5 IRF3 BCL2 SKP2 GSK3A GSK3B NPM1 PARP1 IL6
GO:0009056	catabolic process	13/21	TFEB KPNB1 PHB STAT3 PPP1CA TGFB1 SMAD3 MCL1 BCL2 SKP2 GSK3A GSK3B NPM1
GO:0009058	biosynthetic process	17/21	TFEB KPNB1 STAT5A PHB JUN STAT3 PPP1CA TGFB1 SMAD3 ATF5 IRF3 BCL2 GSK3A GSK3B NPM1 PARP1 IL6
GO:0009892	negative regulation of metabolic process	14/21	PHB JUN STAT3 TGFB1 SMAD3 ATF5 IRF3 MCL1 BCL2 GSK3A GSK3B NPM1 PARP1 IL6
GO:0009893	positive regulation of metabolic process	15/21	TFEB PHB JUN STAT3 TGFB1 SMAD3 ATF5 IRF3 BCL2 SKP2 GSK3A GSK3B NPM1 PARP1 IL6
GO:0019222	regulation of metabolic process	19/21	TFEB KPNB1 STAT5A PHB JUN STAT3 PPP1CA TGFB1 SMAD3 ATF5 IRF3 MCL1 BCL2 SKP2 GSK3A GSK3B NPM1 PARP1 IL6

ID	Description	GeneRatio	geneID
GO:0019748	secondary metabolic process	1/21	BCL2
GO:0032259	methylation	1/21	PARP1
GO:0032963	collagen metabolic process	2/21	TGFB1 IL6
GO:0042440	pigment metabolic process	1/21	BCL2
GO:0044237	cellular metabolic process	19/21	TFEB KPNB1 STAT5A PHB JUN STAT3 PPP1CA TGFB1 SMAD3 ATF5 IRF3 MCL1 BCL2 SKP2 GSK3A GSK3B NPM1 PARP1 IL6
GO:0044238	primary metabolic process	18/21	TFEB KPNB1 STAT5A PHB JUN STAT3 PPP1CA TGFB1 SMAD3 ATF5 IRF3 BCL2 SKP2 GSK3A GSK3B NPM1 PARP1 IL6
GO:0044281	small molecule metabolic process	8/21	KPNB1 STAT5A STAT3 PPP1CA TGFB1 GSK3A GSK3B PARP1
GO:0071704	organic substance metabolic process	18/21	TFEB KPNB1 STAT5A PHB JUN STAT3 PPP1CA TGFB1 SMAD3 ATF5 IRF3 BCL2 SKP2 GSK3A GSK3B NPM1 PARP1 IL6
GO:0001776	leukocyte homeostasis	3/21	TGFB1 BCL2 IL6
GO:0002200	somatic diversification of immune receptors	1/21	TGFB1
GO:0002252	immune effector process	8/21	KPNB1 PHB STAT3 TGFB1 IRF3 BCL2 SKP2 IL6
GO:0002253	activation of immune response	3/21	PHB IRF3 BCL2
GO:0002262	myeloid cell homeostasis	2/21	STAT3 IL6
GO:0002440	production of molecular mediator of immune response	2/21	TGFB1 IL6
GO:0002507	tolerance induction	1/21	TGFB1
GO:0002520	immune system development	7/21	JUN STAT3 TGFB1 SMAD3 BCL2 PARP1 IL6
GO:0002682	regulation of immune system process	8/21	PHB JUN STAT3 TGFB1 SMAD3 IRF3 BCL2 IL6
GO:0002683	negative regulation of immune system process	1/21	TGFB1
GO:0002684	positive regulation of immune system process	7/21	PHB JUN STAT3 TGFB1 IRF3 BCL2 IL6
GO:0006955	immune response	11/21	TFEB KPNB1 PHB JUN STAT3 TGFB1 SMAD3 IRF3 BCL2 SKP2 IL6
GO:0045058	T cell selection	3/21	STAT3 BCL2 IL6
GO:0045321	leukocyte activation	7/21	KPNB1 JUN STAT3 TGFB1 SMAD3 BCL2 IL6
GO:0050900	leukocyte migration	2/21	TGFB1 IL6
GO:0016049	cell growth	6/21	PHB TGFB1 SMAD3 BCL2 GSK3A GSK3B
GO:0040008	regulation of growth	9/21	STAT5A PHB STAT3 TGFB1 SMAD3 BCL2L1 BCL2 GSK3A GSK3B
GO:0045926	negative regulation of growth	5/21	PHB TGFB1 SMAD3 BCL2 GSK3A
GO:0045927	positive regulation of growth	1/21	BCL2
GO:0048589	developmental growth	8/21	STAT5A STAT3 TGFB1 SMAD3 ATF5 BCL2 GSK3A GSK3B

ID	Description	GeneRatio	geneID
GO:0002209	behavioral defense response	1/21	BCL2
GO:0007611	learning or memory	1/21	JUN
GO:0007631	feeding behavior	1/21	STAT3
GO:0050795	regulation of behavior	1/21	STAT3
GO:0008284	positive regulation of cell proliferation	9/21	STAT5A JUN STAT3 TGFB1 BCL2L1 BCL2 SKP2 NPM1 IL6
GO:0008285	negative regulation of cell proliferation	9/21	PHB JUN STAT3 TGFB1 SMAD3 ATF5 BCL2 NPM1 IL6
GO:0014009	glial cell proliferation	1/21	IL6
GO:0033002	muscle cell proliferation	4/21	JUN STAT3 SKP2 IL6
GO:0033687	osteoblast proliferation	2/21	SMAD3 BCL2
GO:0042127	regulation of cell proliferation	12/21	STAT5A PHB JUN STAT3 TGFB1 SMAD3 ATF5 BCL2L1 BCL2 SKP2 NPM1 IL6
GO:0048144	fibroblast proliferation	2/21	JUN TGFB1
GO:0050673	epithelial cell proliferation	5/21	STAT5A JUN STAT3 TGFB1 SMAD3
GO:0061351	neural precursor cell proliferation	2/21	TGFB1 ATF5
GO:0070661	leukocyte proliferation	3/21	TGFB1 BCL2 IL6
GO:0072089	stem cell proliferation	1/21	TGFB1
GO:0007059	chromosome segregation	1/21	KPNB1
GO:0007017	microtubule-based process	4/21	KPNB1 ATF5 GSK3B NPM1
GO:0000075	cell cycle checkpoint	2/21	TGFB1 BCL2L1
GO:0001775	cell activation	7/21	KPNB1 JUN STAT3 TGFB1 SMAD3 BCL2 IL6
GO:0007165	signal transduction	18/21	KPNB1 STAT5A PHB JUN STAT3 PPP1CA TGFB1 SMAD3 IRF3 BCL2L1 MCL1 BCL2 SKP2 GSK3A GSK3B NPM1 PARP1 IL6
GO:0006928	movement of cell or subcellular component	8/21	KPNB1 STAT5A JUN STAT3 TGFB1 SMAD3 BCL2 IL6
GO:0007049	cell cycle	11/21	KPNB1 JUN STAT3 PPP1CA TGFB1 SMAD3 ATF5 BCL2L1 BCL2 SKP2 NPM1
GO:0007154	cell communication	18/21	KPNB1 STAT5A PHB JUN STAT3 PPP1CA TGFB1 SMAD3 IRF3 BCL2L1 MCL1 BCL2 SKP2 GSK3A GSK3B NPM1 PARP1 IL6
GO:0007163	establishment or maintenance of cell polarity	1/21	GSK3B
GO:0007272	ensheathment of neurons	1/21	TGFB1
GO:0008219	cell death	18/21	KPNB1 PHB JUN STAT3 PPP1CA TGFB1 SMAD3 ATF5 IRF3 BCL2L1 MCL1 BCL2 SKP2 GSK3A GSK3B NPM1 PARP1 IL6
GO:0016043	cellular component organization	16/21	TFEB KPNB1 PHB JUN STAT3 PPP1CA TGFB1 SMAD3 ATF5 BCL2L1 BCL2 GSK3A GSK3B NPM1 PARP1 IL6
GO:0016458	gene silencing	3/21	STAT3 TGFB1 SMAD3

ID	Description	GeneRatio	geneID
GO:0019725	cellular homeostasis	4/21	TGFB1 SMAD3 MCL1 BCL2
GO:0022402	cell cycle process	8/21	KPNB1 TGFB1 SMAD3 ATF5 BCL2L1 BCL2 SKP2 NPM1
GO:0022412	cellular process involved in reproduction in multicellular organism	3/21	TGFB1 BCL2L1 BCL2
GO:0030029	actin filament-based process	3/21	TGFB1 SMAD3 BCL2
GO:0032940	secretion by cell	5/21	KPNB1 TGFB1 IRF3 GSK3B IL6
GO:0033059	cellular pigmentation	1/21	BCL2
GO:0044764	multi-organism cellular process	1/21	IRF3
GO:0048522	positive regulation of cellular process	19/21	TFEB STAT5A PHB JUN STAT3 PPP1CA TGFB1 SMAD3 ATF5 IRF3 BCL2L1 MCL1 BCL2 SKP2 GSK3A GSK3B NPM1 PARP1 IL6
GO:0048523	negative regulation of cellular process	15/21	PHB JUN STAT3 TGFB1 SMAD3 ATF5 IRF3 BCL2L1 MCL1 BCL2 GSK3A GSK3B NPM1 PARP1 IL6
GO:0048869	cellular developmental process	14/21	PHB JUN STAT3 TGFB1 SMAD3 ATF5 BCL2L1 MCL1 BCL2 GSK3A GSK3B NPM1 PARP1 IL6
GO:0050794	regulation of cellular process	20/21	TFEB KPNB1 STAT5A PHB JUN STAT3 PPP1CA TGFB1 SMAD3 ATF5 IRF3 BCL2L1 MCL1 BCL2 SKP2 GSK3A GSK3B NPM1 PARP1 IL6
GO:0051301	cell division	3/21	PPP1CA TGFB1 BCL2L1
GO:0051716	cellular response to stimulus	18/21	KPNB1 STAT5A PHB JUN STAT3 PPP1CA TGFB1 SMAD3 IRF3 BCL2L1 MCL1 BCL2 SKP2 GSK3A GSK3B NPM1 PARP1 IL6
GO:0061919	process utilizing autophagic mechanism	4/21	TFEB MCL1 BCL2 GSK3A
GO:0097194	execution phase of apoptosis	2/21	KPNB1 BCL2L1
GO:0006808	regulation of nitrogen utilization	1/21	BCL2
GO:0003006	developmental process involved in reproduction	5/21	TFEB PHB TGFB1 BCL2L1 BCL2
GO:0009566	fertilization	1/21	BCL2L1
GO:0044703	multi-organism reproductive process	4/21	STAT3 TGFB1 BCL2L1 BCL2
GO:0048609	multicellular organismal reproductive process	3/21	TGFB1 BCL2L1 BCL2
GO:0007155	cell adhesion	6/21	PPP1CA TGFB1 SMAD3 BCL2 GSK3B IL6
GO:0007267	cell-cell signaling	6/21	STAT3 PPP1CA SMAD3 GSK3A GSK3B IL6
GO:0023051	regulation of signaling	15/21	PHB JUN STAT3 PPP1CA TGFB1 SMAD3 IRF3 BCL2L1 MCL1 BCL2 SKP2 GSK3A GSK3B PARP1 IL6
GO:0023056	positive regulation of signaling	15/21	PHB JUN STAT3 PPP1CA TGFB1 SMAD3 IRF3 BCL2L1 MCL1 BCL2 SKP2 GSK3A GSK3B PARP1 IL6
GO:0023057	negative regulation of signaling	9/21	PHB TGFB1 SMAD3 BCL2L1 MCL1 BCL2 GSK3A GSK3B IL6
GO:0001503	ossification	5/21	PHB TGFB1 SMAD3 BCL2 IL6
GO:0001763	morphogenesis of a branching structure	3/21	PPP1CA TGFB1 BCL2

ID	Description	GeneRatio	geneID
GO:0001816	cytokine production	4/21	TGFB1 SMAD3 IRF3 IL6
GO:0003008	system process	7/21	JUN TGFB1 SMAD3 BCL2 GSK3A GSK3B PARP1
GO:0007275	multicellular organism development	16/21	TFEB STAT5A PHB JUN STAT3 PPP1CA TGFB1 SMAD3 ATF5 BCL2L1 MCL1 BCL2 GSK3A GSK3B PARP1 IL6
GO:0007389	pattern specification process	1/21	SMAD3
GO:0009791	post-embryonic development	2/21	ATF5 BCL2
GO:0019827	stem cell population maintenance	1/21	STAT3
GO:0022404	molting cycle process	1/21	BCL2
GO:0032922	circadian regulation of gene expression	1/21	PPP1CA
GO:0033555	multicellular organismal response to stress	1/21	BCL2
GO:0035264	multicellular organism growth	4/21	STAT5A STAT3 ATF5 BCL2
GO:0035265	organ growth	2/21	BCL2 GSK3A
GO:0042303	molting cycle	1/21	BCL2
GO:0044706	multi-multicellular organism process	2/21	TGFB1 BCL2
GO:0048771	tissue remodeling	2/21	TGFB1 IL6
GO:0048871	multicellular organismal homeostasis	3/21	STAT3 BCL2 IL6
GO:0050817	coagulation	1/21	IL6
GO:0051239	regulation of multicellular organismal process	11/21	STAT5A JUN STAT3 TGFB1 SMAD3 IRF3 BCL2 GSK3A GSK3B PARP1 IL6
GO:0051240	positive regulation of multicellular organismal process	10/21	STAT5A JUN STAT3 TGFB1 SMAD3 IRF3 BCL2 GSK3A PARP1 IL6
GO:0051241	negative regulation of multicellular organismal process	8/21	STAT3 TGFB1 SMAD3 IRF3 BCL2 GSK3A GSK3B IL6
GO:0090130	tissue migration	3/21	STAT5A JUN TGFB1
GO:0009653	anatomical structure morphogenesis	10/21	JUN STAT3 PPP1CA TGFB1 SMAD3 BCL2L1 BCL2 GSK3A GSK3B IL6
GO:0007568	aging	5/21	JUN STAT3 TGFB1 BCL2 NPM1
GO:0048646	anatomical structure formation involved in morphogenesis	5/21	JUN STAT3 TGFB1 SMAD3 IL6
GO:0021700	developmental maturation	1/21	BCL2
GO:0048856	anatomical structure development	16/21	TFEB STAT5A PHB JUN STAT3 PPP1CA TGFB1 SMAD3 ATF5 BCL2L1 MCL1 BCL2 GSK3A GSK3B PARP1 IL6
GO:0050793	regulation of developmental process	10/21	STAT5A JUN STAT3 TGFB1 SMAD3 BCL2 GSK3A GSK3B PARP1 IL6
GO:0051093	negative regulation of developmental process	7/21	STAT3 TGFB1 SMAD3 BCL2 GSK3A GSK3B IL6
GO:0051094	positive regulation of developmental process	7/21	JUN STAT3 TGFB1 SMAD3 BCL2 PARP1 IL6

ID	Description	GeneRatio	geneID
GO:0098727	maintenance of cell number	1/21	STAT3
GO:0040012	regulation of locomotion	7/21	STAT5A JUN STAT3 TGFB1 SMAD3 BCL2 IL6
GO:0040013	negative regulation of locomotion	3/21	STAT3 TGFB1 BCL2
GO:0040017	positive regulation of locomotion	7/21	STAT5A JUN STAT3 TGFB1 SMAD3 BCL2 IL6
GO:0042330	taxis	3/21	TGFB1 SMAD3 IL6
GO:0048870	cell motility	7/21	STAT5A JUN STAT3 TGFB1 SMAD3 BCL2 IL6
GO:0048066	developmental pigmentation	1/21	BCL2
GO:0007623	circadian rhythm	4/21	JUN PPP1CA ATF5 GSK3B
GO:0043902	positive regulation of multi-organism process	1/21	JUN
GO:0044089	positive regulation of cellular component biogenesis	5/21	JUN TGFB1 SMAD3 GSK3B PARP1
GO:0045785	positive regulation of cell adhesion	4/21	TGFB1 SMAD3 GSK3B IL6
GO:0048087	positive regulation of developmental pigmentation	1/21	BCL2
GO:0048584	positive regulation of response to stimulus	15/21	PHB JUN STAT3 PPP1CA TGFB1 SMAD3 IRF3 BCL2L1 MCL1 BCL2 SKP2 GSK3A GSK3B PARP1 IL6
GO:0051050	positive regulation of transport	7/21	TGFB1 SMAD3 IRF3 BCL2 GSK3A GSK3B IL6
GO:0051091	positive regulation of DNA-binding transcription factor activity	4/21	TGFB1 SMAD3 NPM1 IL6
GO:1903829	positive regulation of cellular protein localization	6/21	TGFB1 SMAD3 BCL2 GSK3A GSK3B PARP1
GO:1904181	positive regulation of membrane depolarization	1/21	PARP1
GO:1904951	positive regulation of establishment of protein localization	7/21	TGFB1 SMAD3 IRF3 BCL2 GSK3A GSK3B IL6
GO:0043901	negative regulation of multi-organism process	1/21	JUN
GO:0048585	negative regulation of response to stimulus	9/21	PHB TGFB1 SMAD3 BCL2L1 MCL1 BCL2 GSK3A GSK3B IL6
GO:0051051	negative regulation of transport	3/21	TGFB1 BCL2 GSK3A
GO:1903828	negative regulation of cellular protein localization	3/21	TGFB1 BCL2L1 GSK3B
GO:1904180	negative regulation of membrane depolarization	1/21	BCL2
GO:1905953	negative regulation of lipid localization	1/21	IL6
GO:0030155	regulation of cell adhesion	5/21	TGFB1 SMAD3 BCL2 GSK3B IL6
GO:0032879	regulation of localization	12/21	STAT5A JUN STAT3 TGFB1 SMAD3 IRF3 BCL2L1 BCL2 GSK3A GSK3B PARP1 IL6
GO:0042752	regulation of circadian rhythm	1/21	PPP1CA

ID	Description	GeneRatio	geneID
GO:0043900	regulation of multi-organism process	2/21	JUN BCL2
GO:0044087	regulation of cellular component biogenesis	6/21	JUN TGFB1 SMAD3 GSK3B NPM1 PARP1
GO:0048070	regulation of developmental pigmentation	1/21	BCL2
GO:0048518	positive regulation of biological process	19/21	TFEB STAT5A PHB JUN STAT3 PPP1CA TGFB1 SMAD3 ATF5 IRF3 BCL2L1 MCL1 BCL2 SKP2 GSK3A GSK3B NPM1 PARP1 IL6
GO:0048519	negative regulation of biological process	15/21	PHB JUN STAT3 TGFB1 SMAD3 ATF5 IRF3 BCL2L1 MCL1 BCL2 GSK3A GSK3B NPM1 PARP1 IL6
GO:0048583	regulation of response to stimulus	16/21	PHB JUN STAT3 PPP1CA TGFB1 SMAD3 IRF3 BCL2L1 MCL1 BCL2 SKP2 GSK3A GSK3B NPM1 PARP1 IL6
GO:0006950	response to stress	16/21	PHB JUN STAT3 PPP1CA TGFB1 SMAD3 IRF3 BCL2L1 MCL1 BCL2 SKP2 GSK3A GSK3B NPM1 PARP1 IL6
GO:0009605	response to external stimulus	10/21	PHB JUN PPP1CA TGFB1 SMAD3 IRF3 BCL2L1 BCL2 SKP2 IL6
GO:0009607	response to biotic stimulus	9/21	JUN TGFB1 SMAD3 IRF3 BCL2L1 BCL2 SKP2 GSK3B IL6
GO:0009628	response to abiotic stimulus	9/21	JUN PPP1CA TGFB1 SMAD3 BCL2L1 BCL2 GSK3B NPM1 PARP1
GO:0009719	response to endogenous stimulus	13/21	STAT5A PHB JUN STAT3 TGFB1 SMAD3 BCL2L1 BCL2 SKP2 GSK3A GSK3B PARP1 IL6
GO:0042221	response to chemical	17/21	STAT5A PHB JUN STAT3 PPP1CA TGFB1 SMAD3 IRF3 BCL2L1 MCL1 BCL2 SKP2 GSK3A GSK3B NPM1 PARP1 IL6
GO:0043500	muscle adaptation	3/21	SMAD3 GSK3A PARP1
GO:0051606	detection of stimulus	1/21	PARP1
GO:0072376	protein activation cascade	1/21	PHB
GO:0031503	protein-containing complex localization	1/21	NPM1
GO:0033036	macromolecule localization	13/21	KPNB1 STAT3 TGFB1 SMAD3 IRF3 BCL2L1 MCL1 BCL2 GSK3A GSK3B NPM1 PARP1 IL6
GO:0051234	establishment of localization	14/21	KPNB1 STAT5A PHB STAT3 TGFB1 SMAD3 IRF3 BCL2L1 MCL1 BCL2 GSK3A GSK3B NPM1 IL6
GO:0051235	maintenance of location	2/21	TGFB1 IL6
GO:0051641	cellular localization	10/21	KPNB1 STAT3 TGFB1 SMAD3 BCL2L1 BCL2 GSK3A GSK3B NPM1 PARP1
GO:0051674	localization of cell	7/21	STAT5A JUN STAT3 TGFB1 SMAD3 BCL2 IL6
GO:1902579	multi-organism localization	1/21	KPNB1
GO:0051707	response to other organism	8/21	JUN TGFB1 SMAD3 IRF3 BCL2L1 BCL2 SKP2 IL6
GO:0044419	interspecies interaction between organisms	9/21	KPNB1 JUN STAT3 TGFB1 SMAD3 IRF3 BCL2L1 BCL2 NPM1
GO:0050789	regulation of biological process	20/21	TFEB KPNB1 STAT5A PHB JUN STAT3 PPP1CA TGFB1 SMAD3 ATF5 IRF3 BCL2L1 MCL1 BCL2 SKP2 GSK3A GSK3B NPM1 PARP1 IL6
GO:0065008	regulation of biological quality	15/21	STAT5A PHB JUN STAT3 PPP1CA TGFB1 SMAD3 BCL2L1 MCL1 BCL2 GSK3A GSK3B NPM1 PARP1 IL6

ID	Description	GeneRatio	geneID
GO:0065009	regulation of molecular function	11/21	JUN STAT3 PPP1CA TGFB1 SMAD3 BCL2 GSK3A GSK3B NPM1 PARP1 IL6
GO:0044085	cellular component biogenesis	8/21	KPNB1 JUN TGFB1 SMAD3 BCL2 GSK3B NPM1 PARP1

GO ENRICHMENT:

1 HCOV-HOST_SET

1.1 GO ENRICHMENT

GO Enrichment Analysis after FDR control of a gene set.

MOLECULAR FUNCTION

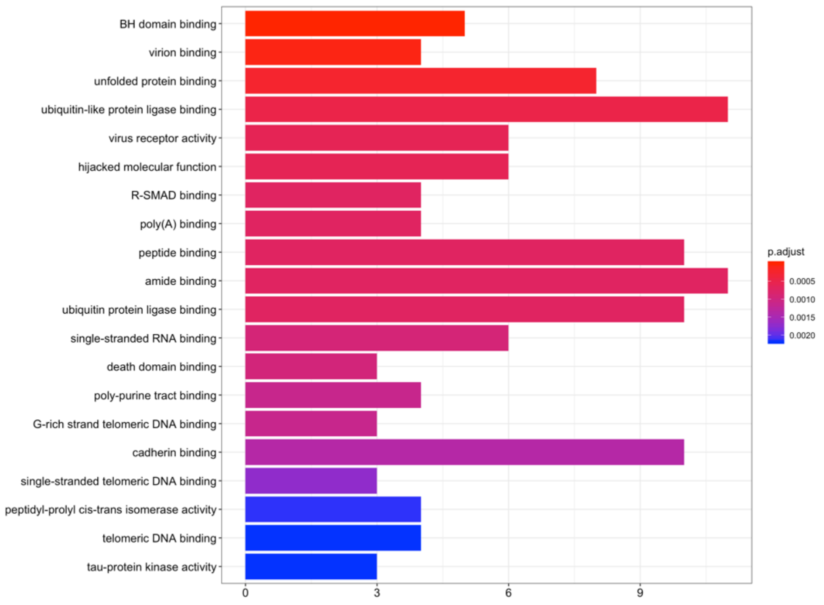

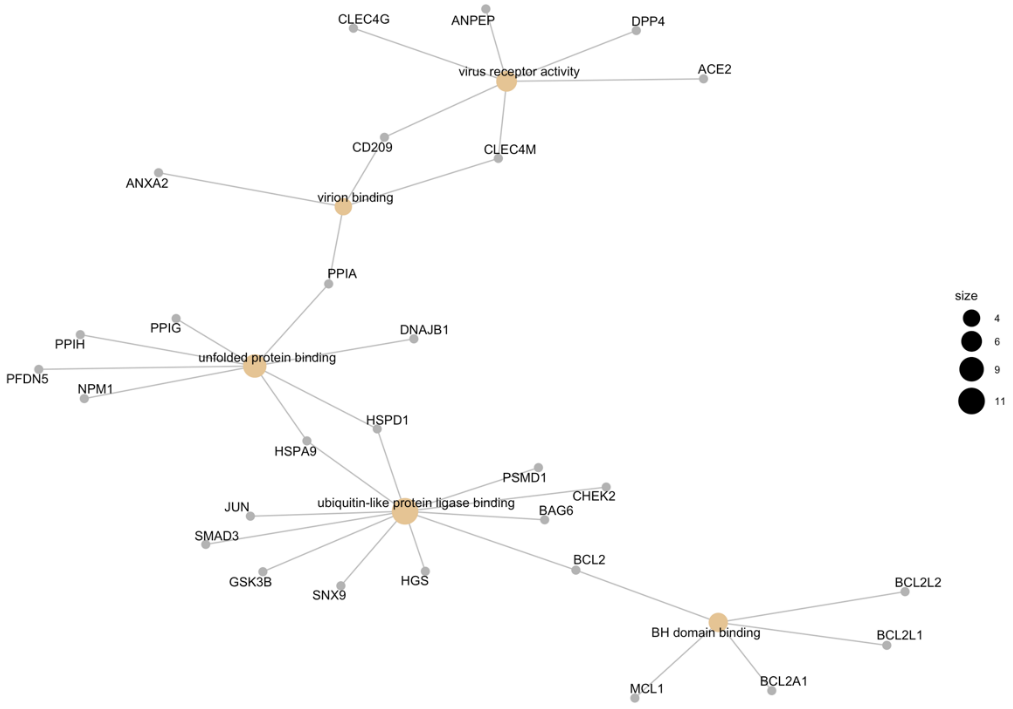

CELLULAR COMPONENT

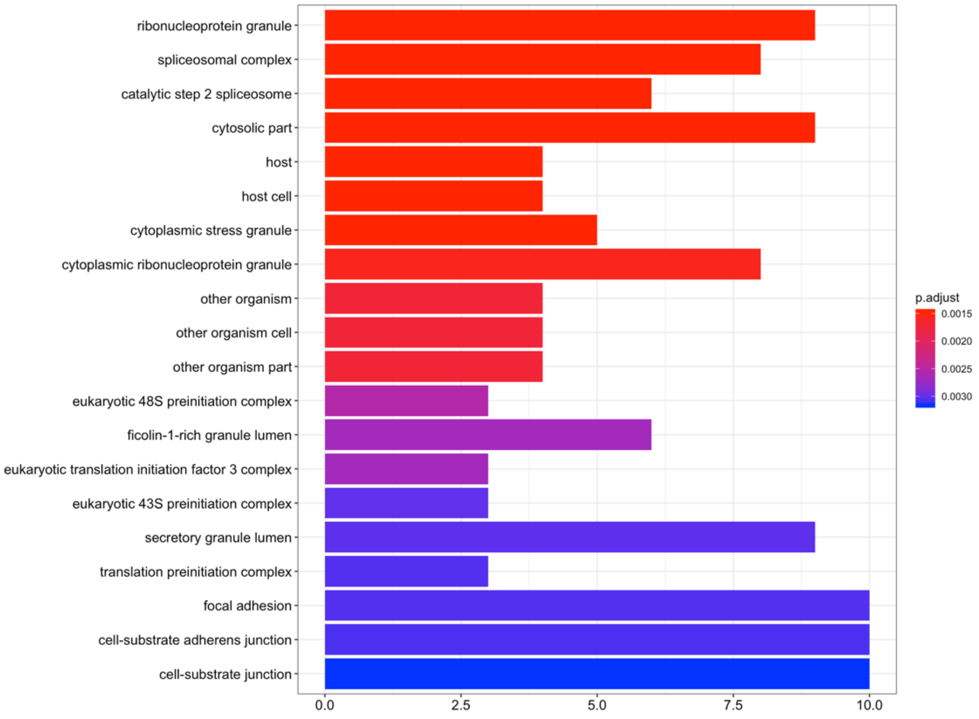

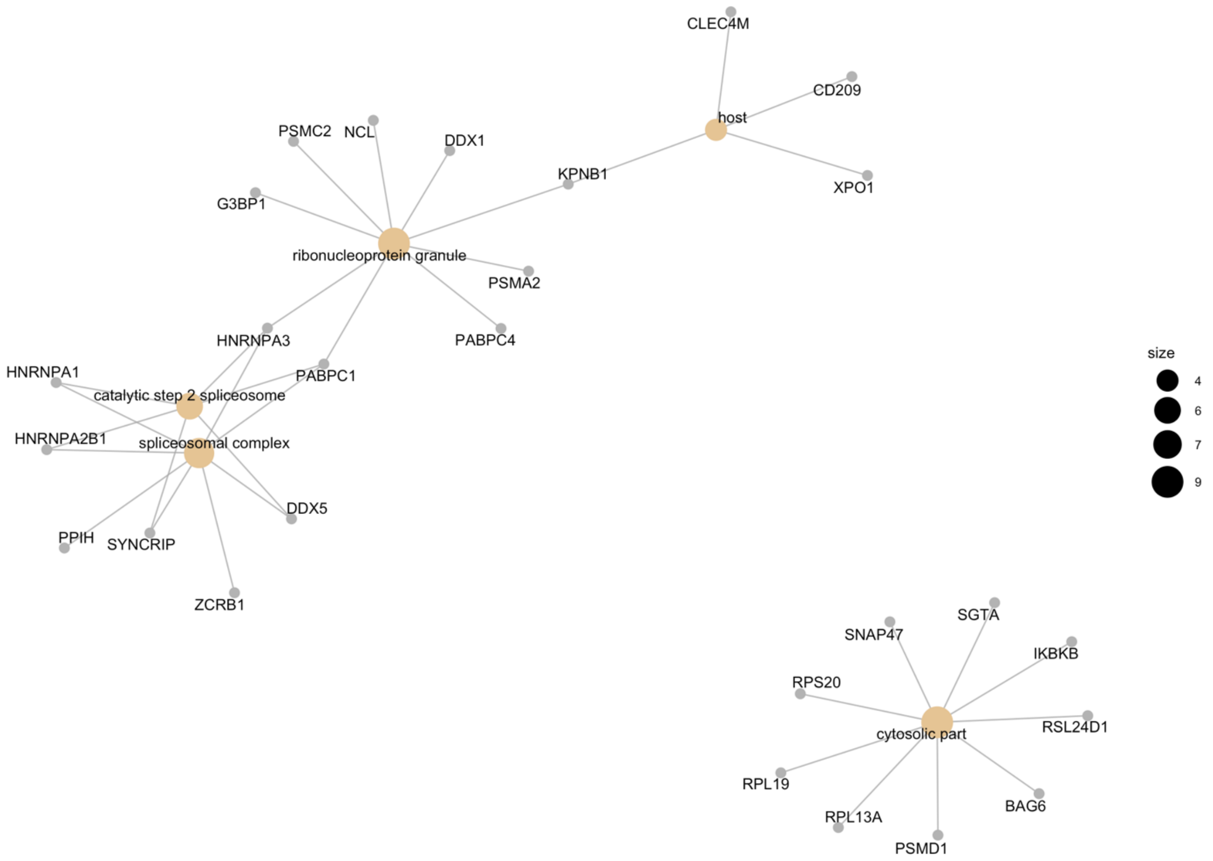

BIOLOGICAL PROCESS

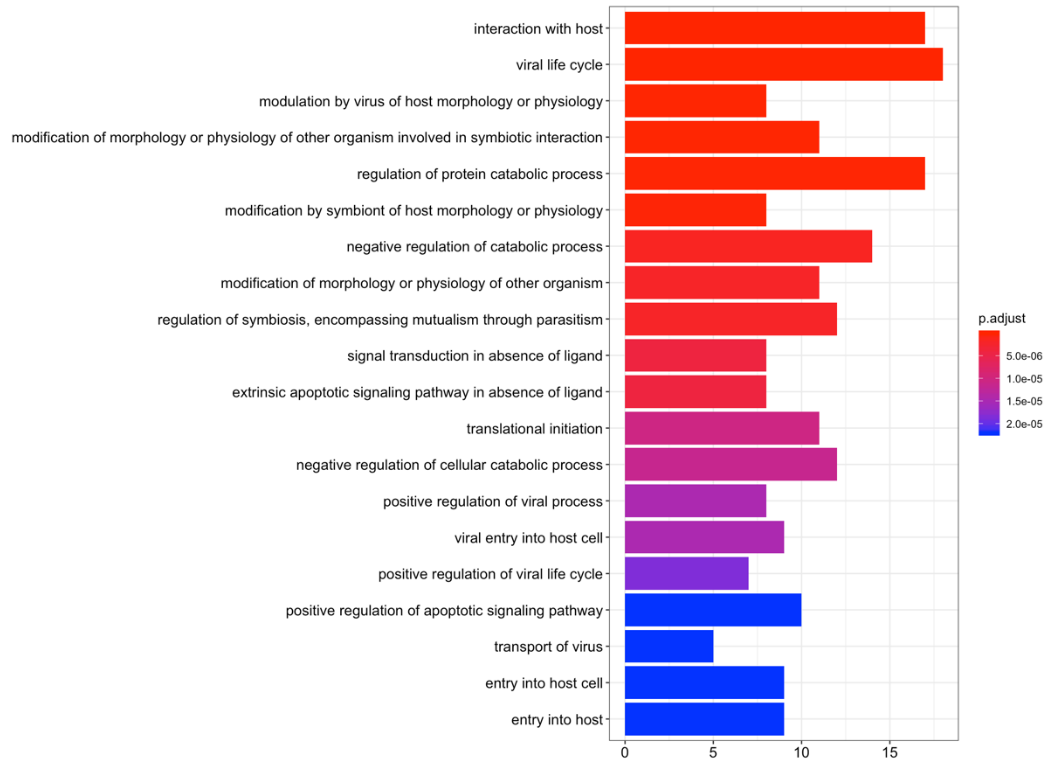

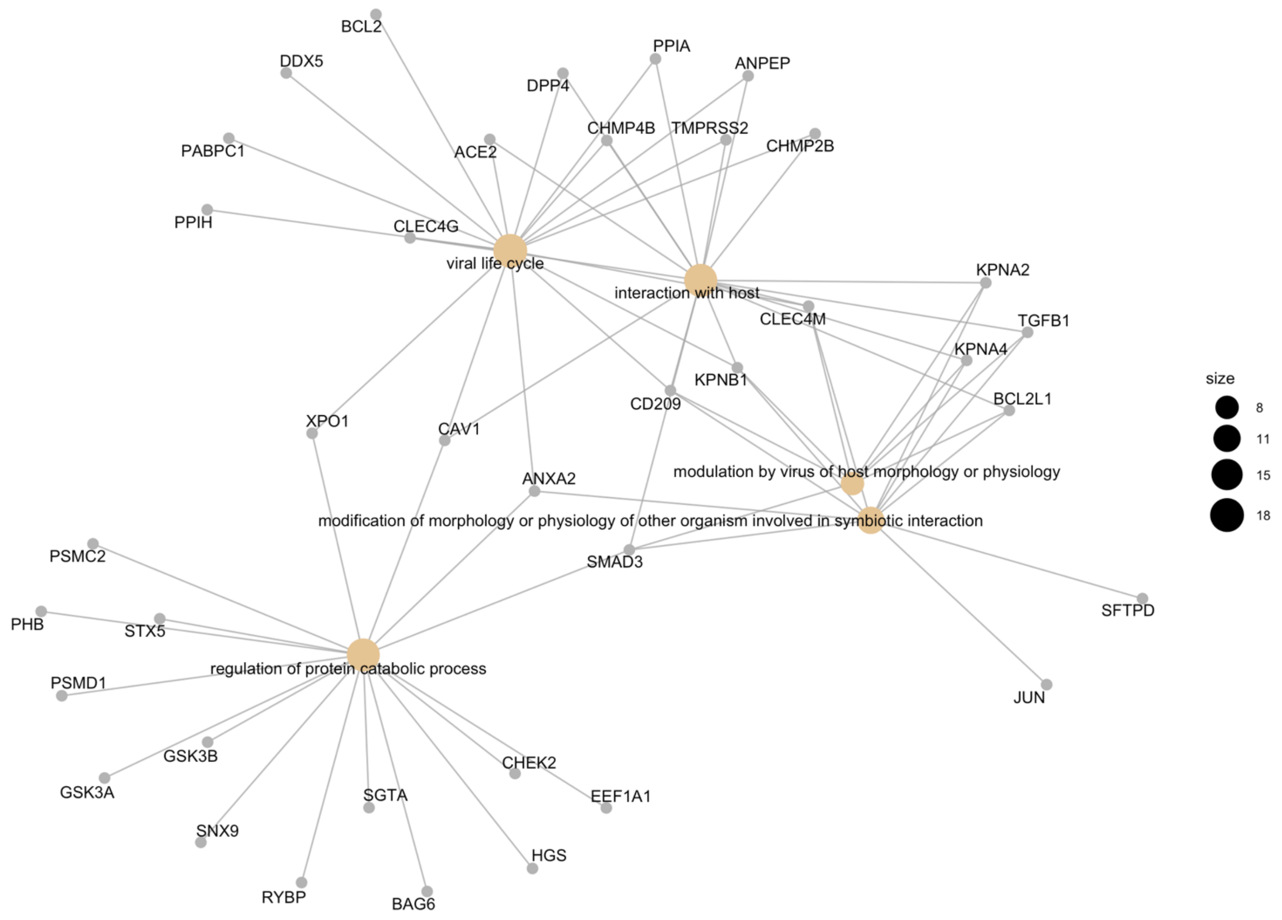

1.2 TABLES ENRICHMENT

1.2.1 MOLECULAR FUNCTION

ID	Description	GeneRatio	BgRatio	p.adjust	geneID
GO:0051400	BH domain binding	5/117	10/17632	0	BCL2L1 BCL2L2 MCL1 BCL2A1 BCL2
GO:0046790	virion binding	4/117	10/17632	0	CD209 CLEC4M PPIA ANXA2
GO:0051082	unfolded protein binding	8/117	127/17632	0	PPIA DNAJB1 HSPD1 HSPA9 NPM1 PPIG PPIH PFDN5
GO:0044389	ubiquitin-like protein ligase binding	11/117	301/17632	0	JUN SMAD3 BCL2 HSPD1 HSPA9 PSMD1 SNX9 GSK3B HGS BAG6 CHEK2
GO:0001618	virus receptor activity	6/117	74/17632	0.001	ACE2 CLEC4G CD209 CLEC4M ANPEP DPP4
GO:0104005	hijacked molecular function	6/117	74/17632	0.001	ACE2 CLEC4G CD209 CLEC4M ANPEP DPP4
GO:0070412	R-SMAD binding	4/117	23/17632	0.001	JUN SMAD3 PARP1 DDX5
GO:0008143	poly(A) binding	4/117	24/17632	0.001	SYNCRIP PABPC1 PABPC4 DDX1
GO:0042277	peptide binding	10/117	285/17632	0.001	KPNB1 CD209 CLEC4M KPNA2 PPIA ANPEP HSPD1 SRP54 PPIG PPIH
GO:0033218	amide binding	11/117	348/17632	0.001	KPNB1 ACBD5 CD209 CLEC4M KPNA2 PPIA ANPEP HSPD1 SRP54 PPIG PPIH
GO:0031625	ubiquitin protein ligase binding	10/117	286/17632	0.001	JUN SMAD3 BCL2 HSPD1 HSPA9 PSMD1 SNX9 GSK3B BAG6 CHEK2
GO:0003727	single-stranded RNA binding	6/117	90/17632	0.001	HNRNPA1 SYNCRIP PTBP1 PABPC1 PABPC4 DDX1
GO:0070513	death domain binding	3/117	10/17632	0.001	BCL2L1 MCL1 BCL2
GO:0070717	poly-purine tract binding	4/117	30/17632	0.001	SYNCRIP PABPC1 PABPC4 DDX1
GO:0098505	G-rich strand telomeric DNA binding	3/117	11/17632	0.001	HNRNPA1 HNRNPA2B1 TERF1
GO:0045296	cadherin binding	10/117	323/17632	0.001	TWF2 YKT6 STX5 PPP1CA ANXA2 DNAJB1 EIF3E CHMP4B SNX9 CHMP2B
GO:0043047	single-stranded telomeric DNA binding	3/117	13/17632	0.002	HNRNPA1 HNRNPA2B1 TERF1
GO:0003755	peptidyl-prolyl cis-trans isomerase activity	4/117	37/17632	0.002	PPIA FKBP1A PPIG PPIH
GO:0042162	telomeric DNA binding	4/117	38/17632	0.002	HNRNPA1 HNRNPA2B1 NCL TERF1
GO:0050321	tau-protein kinase activity	3/117	15/17632	0.002	GSK3A GSK3B MARK3
GO:0098847	sequence-specific single stranded DNA binding	3/117	15/17632	0.002	HNRNPA1 HNRNPA2B1 TERF1

ID	Description	GeneRatio	BgRatio	p.adjust	geneID
GO:0016859	cis-trans isomerase activity	4/117	41/17632	0.003	PPIA FKBP1A PPIG PPIH
GO:0002020	protease binding	6/117	125/17632	0.003	BCL2 DPP4 ANXA2 HSPD1 GSK3B BAG6
GO:0046332	SMAD binding	5/117	80/17632	0.003	JUN SMAD3 PARP1 FKBP1A DDX5
GO:0043021	ribonucleoprotein complex binding	6/117	128/17632	0.003	PPP1CA SRP54 NPM1 PPIH BAG6 DDX5
GO:0016018	cyclosporin A binding	3/117	19/17632	0.004	PPIA PPIG PPIH
GO:0140142	nucleocytoplasmic carrier activity	3/117	19/17632	0.004	XPO1 KPNA2 KPNA4
GO:0008565	protein transporter activity	5/117	88/17632	0.004	KPNB1 XPO1 KPNA2 MCL1 KPNA4
GO:0050839	cell adhesion molecule binding	11/117	486/17632	0.005	TWF2 YKT6 STX5 PPP1CA CD9 ANXA2 DNAJB1 EIF3E CHMP4B SNX9 CHMP2B
GO:0047485	protein N-terminus binding	5/117	107/17632	0.009	SCFD1 STX5 TGFB1 EIF3E PARP1
GO:0036002	pre-mRNA binding	4/117	62/17632	0.009	HNRNPA1 PTBP1 HNRNPA2B1 DDX5

1.2.2 CELLULAR COMPONENT

ID	Description	GeneRatio	BgRatio	p.adjust	geneID
GO:0035770	ribonucleoprotein granule	9/119	219/19659	0.001	PSMC2 KPNB1 HNRNPA3 PABPC1 PABPC4 G3BP1 NCL DDX1 PSMA2
GO:0005681	spliceosomal complex	8/119	179/19659	0.001	HNRNPA1 ZCRB1 SYNCRIP HNRNPA2B1 HNRNPA3 PABPC1 PPIH DDX5
GO:0071013	catalytic step 2 spliceosome	6/119	87/19659	0.001	HNRNPA1 SYNCRIP HNRNPA2B1 HNRNPA3 PABPC1 DDX5
GO:0044445	cytosolic part	9/119	250/19659	0.001	RSL24D1 IKBKB RPL13A PSMD1 RPL19 BAG6 SNAP47 RPS20 SGTA
GO:0018995	host	4/119	29/19659	0.001	KPNB1 XPO1 CD209 CLEC4M
GO:0043657	host cell	4/119	29/19659	0.001	KPNB1 XPO1 CD209 CLEC4M
GO:0010494	cytoplasmic stress granule	5/119	60/19659	0.001	KPNB1 PABPC1 PABPC4 G3BP1 DDX1
GO:0036464	cytoplasmic ribonucleoprotein granule	8/119	207/19659	0.002	PSMC2 KPNB1 PABPC1 PABPC4 G3BP1 NCL DDX1 PSMA2
GO:0044215	other organism	4/119	35/19659	0.002	KPNB1 XPO1 CD209 CLEC4M
GO:0044216	other organism cell	4/119	35/19659	0.002	KPNB1 XPO1 CD209 CLEC4M
GO:0044217	other organism part	4/119	35/19659	0.002	KPNB1 XPO1 CD209 CLEC4M
GO:0033290	eukaryotic 48S preinitiation complex	3/119	15/19659	0.003	EIF3E EIF3I EIF3F

ID	Description	GeneRatio	BgRatio	p.adjust	geneID
GO:1904813	ficolin-1-rich granule lumen	6/119	124/19659	0.003	PSMC2 KPNB1 FGL2 PPIA PSMA2 EEF1A1
GO:0005852	eukaryotic translation initiation factor 3 complex	3/119	16/19659	0.003	EIF3E EIF3I EIF3F
GO:0016282	eukaryotic 43S preinitiation complex	3/119	17/19659	0.003	EIF3E EIF3I EIF3F
GO:0034774	secretory granule lumen	9/119	321/19659	0.003	PSMC2 KPNB1 TGFB1 PPIA ANXA2 PSMD1 SERPING1 PSMA2 EEF1A1
GO:0070993	translation preinitiation complex	3/119	18/19659	0.003	EIF3E EIF3I EIF3F
GO:0005925	focal adhesion	10/119	402/19659	0.003	PPIA CD9 DPP4 HSPA9 RPL13A PABPC1 NPM1 G3BP1 RPL19 CAV1
GO:0005924	cell-substrate adherens junction	10/119	405/19659	0.003	PPIA CD9 DPP4 HSPA9 RPL13A PABPC1 NPM1 G3BP1 RPL19 CAV1
GO:0030055	cell-substrate junction	10/119	409/19659	0.003	PPIA CD9 DPP4 HSPA9 RPL13A PABPC1 NPM1 G3BP1 RPL19 CAV1
GO:0060205	cytoplasmic vesicle lumen	9/119	338/19659	0.003	PSMC2 KPNB1 TGFB1 PPIA ANXA2 PSMD1 SERPING1 PSMA2 EEF1A1
GO:0031983	vesicle lumen	9/119	339/19659	0.003	PSMC2 KPNB1 TGFB1 PPIA ANXA2 PSMD1 SERPING1 PSMA2 EEF1A1
GO:0098978	glutamatergic synapse	9/119	350/19659	0.004	PHB STAT3 PPP1CA KPNA2 DNAJB1 GSK3B CHMP2B SNAP47 ABHD17A
GO:0000781	chromosome, telomeric region	6/119	158/19659	0.006	PPP1CA HNRNPA2B1 PARP1 H2AFY2 CHEK2 TERF1
GO:0043209	myelin sheath	6/119	162/19659	0.006	PHB BCL2 ANXA2 ACO2 HSPD1 NDUFA10
GO:0036452	ESCRT complex	3/119	27/19659	0.007	CHMP4B HGS CHMP2B
GO:0005741	mitochondrial outer membrane	6/119	180/19659	0.01	PHB BCL2L1 BCL2L2 MCL1 BCL2A1 BCL2

1.2.3 BIOLOGICAL PROCESS

ID	Description	GeneRatio	BgRatio	p.adjust	geneID
GO:0051701	interaction with host	17/117	209/18493	0	KPNB1 TGFB1 SMAD3 ACE2 CLEC4G CD209 CLEC4M KPNA2 PPIA BCL2L1 KPNA4 TMPRSS2 ANPEP DPP4 CHMP4B CHMP2B CAV1
GO:0019058	viral life cycle	18/117	289/18493	0	KPNB1 XPO1 ACE2 CLEC4G CD209 CLEC4M PPIA BCL2 TMPRSS2 ANPEP DPP4 ANXA2 CHMP4B PABPC1 PPIH CHMP2B DDX5 CAV1

ID	Description	GeneRatio	BgRatio	p.adjust	geneID
GO:0019048	modulation by virus of host morphology or physiology	8/117	38/18493	0	KPNB1 TGFB1 SMAD3 CD209 CLEC4M KPNA2 BCL2L1 KPNA4
GO:0051817	modification of morphology or physiology of other organism involved in symbiotic interaction	11/117	112/18493	0	KPNB1 JUN TGFB1 SMAD3 CD209 CLEC4M KPNA2 SFTPD BCL2L1 KPNA4 ANXA2
GO:0042176	regulation of protein catabolic process	17/117	375/18493	0	PSMC2 STX5 PHB XPO1 SMAD3 ANXA2 PSMD1 SNX9 GSK3A GSK3B RYBP HGS BAG6 CHEK2 EEF1A1 CAV1 SGTA
GO:0044003	modification by symbiont of host morphology or physiology	8/117	46/18493	0	KPNB1 TGFB1 SMAD3 CD209 CLEC4M KPNA2 BCL2L1 KPNA4
GO:0009895	negative regulation of catabolic process	14/117	287/18493	0	SCFD1 PHB STAT3 SMAD3 MCL1 BCL2 SYNCRIP ANXA2 CHMP4B GSK3A PABPC1 RYBP BAG6 SGTA
GO:0035821	modification of morphology or physiology of other organism	11/117	161/18493	0	KPNB1 JUN TGFB1 SMAD3 CD209 CLEC4M KPNA2 SFTPD BCL2L1 KPNA4 ANXA2
GO:0043903	regulation of symbiosis, encompassing mutualism through parasitism	12/117	203/18493	0	JUN SFTPD PPIA BCL2 TMPRSS2 ANXA2 CHMP4B PABPC1 PPIH CHMP2B DDX5 CAV1
GO:0038034	signal transduction in absence of ligand	8/117	71/18493	0	PPP1CA BCL2L1 BCL2L2 MCL1 BCL2A1 BCL2 GSK3A GSK3B
GO:0097192	extrinsic apoptotic signaling pathway in absence of ligand	8/117	71/18493	0	PPP1CA BCL2L1 BCL2L2 MCL1 BCL2A1 BCL2 GSK3A GSK3B
GO:0006413	translational initiation	11/117	194/18493	0	PPP1CA RPL13A EIF3E EIF3I EIF3F PABPC1 NPM1 RPL19 DDX1 MIF4GD RPS20
GO:0031330	negative regulation of cellular catabolic process	12/117	245/18493	0	SCFD1 STAT3 MCL1 BCL2 SYNCRIP ANXA2 CHMP4B GSK3A PABPC1 RYBP BAG6 SGTA
GO:0048524	positive regulation of viral process	8/117	86/18493	0	JUN PPIA TMPRSS2 ANXA2 CHMP4B PABPC1 PPIH CHMP2B
GO:0046718	viral entry into host cell	9/117	121/18493	0	ACE2 CLEC4G CD209 CLEC4M PPIA TMPRSS2 ANPEP DPP4 CAV1
GO:1903902	positive regulation of viral life cycle	7/117	60/18493	0	PPIA TMPRSS2 ANXA2 CHMP4B PABPC1 PPIH CHMP2B
GO:2001235	positive regulation of apoptotic signaling pathway	10/117	170/18493	0	PPP1CA SMAD3 BCL2L1 MCL1 BCL2 PRKRA NMT1 GSK3A GSK3B CAV1
GO:0046794	transport of virus	5/117	20/18493	0	KPNB1 XPO1 CD209 CLEC4M CAV1
GO:0030260	entry into host cell	9/117	133/18493	0	ACE2 CLEC4G CD209 CLEC4M PPIA TMPRSS2 ANPEP DPP4 CAV1
GO:0044409	entry into host	9/117	133/18493	0	ACE2 CLEC4G CD209 CLEC4M PPIA TMPRSS2 ANPEP DPP4 CAV1

ID	Description	GeneRatio	BgRatio	p.adjust	geneID
GO:0051806	entry into cell of other organism involved in symbiotic interaction	9/117	133/18493	0	ACE2 CLEC4G CD209 CLEC4M PPIA TMPRSS2 ANPEP DPP4 CAV1
GO:0051828	entry into other organism involved in symbiotic interaction	9/117	133/18493	0	ACE2 CLEC4G CD209 CLEC4M PPIA TMPRSS2 ANPEP DPP4 CAV1
GO:2001233	regulation of apoptotic signaling pathway	14/117	391/18493	0	PPP1CA SMAD3 BCL2L1 BCL2L2 MCL1 BCL2A1 BCL2 PRKRA NMT1 NONO GSK3A GSK3B PARP1 CAV1
GO:0097191	extrinsic apoptotic signaling pathway	11/117	224/18493	0	PPP1CA TGFB1 SMAD3 BCL2L1 BCL2L2 MCL1 BCL2A1 BCL2 GSK3A GSK3B CAV1
GO:0050792	regulation of viral process	10/117	177/18493	0	JUN PPIA BCL2 TMPRSS2 ANXA2 CHMP4B PABPC1 PPIH CHMP2B DDX5
GO:1903900	regulation of viral life cycle	9/117	140/18493	0	PPIA BCL2 TMPRSS2 ANXA2 CHMP4B PABPC1 PPIH CHMP2B DDX5
GO:0097193	intrinsic apoptotic signaling pathway	12/117	287/18493	0	BCL2L1 BCL2L2 MCL1 BCL2A1 BCL2 PRKRA NONO PARP1 BAG6 CHEK2 DDX5 CAV1
GO:0044650	adhesion of symbiont to host cell	4/117	12/18493	0	ACE2 CD209 CLEC4M SFTPD
GO:2001242	regulation of intrinsic apoptotic signaling pathway	9/117	161/18493	0	BCL2L1 BCL2L2 MCL1 BCL2A1 BCL2 PRKRA NONO PARP1 CAV1
GO:0007183	SMAD protein complex assembly	4/117	13/18493	0	TGFB1 SMAD3 PARP1 FKBP1A
GO:0046605	regulation of centrosome cycle	6/117	56/18493	0	XPO1 ATF5 CHMP4B KIF11 NPM1 CHMP2B
GO:0072593	reactive oxygen species metabolic process	11/117	276/18493	0	STAT3 TGFB1 SMAD3 COX2 ACE2 SFTPD BCL2 HSPD1 GBF1 DDAH2 CAV1
GO:0044766	multi-organism transport	5/117	33/18493	0	KPNB1 XPO1 CD209 CLEC4M CAV1
GO:1902579	multi-organism localization	5/117	33/18493	0	KPNB1 XPO1 CD209 CLEC4M CAV1
GO:0044406	adhesion of symbiont to host	4/117	15/18493	0	ACE2 CD209 CLEC4M SFTPD
GO:2000377	regulation of reactive oxygen species metabolic process	9/117	185/18493	0	STAT3 TGFB1 SMAD3 COX2 ACE2 BCL2 HSPD1 DDAH2 CAV1
GO:0075733	intracellular transport of virus	4/117	17/18493	0	KPNB1 XPO1 CD209 CLEC4M
GO:0008630	intrinsic apoptotic signaling pathway in response to DNA damage	7/117	102/18493	0	BCL2L1 BCL2L2 MCL1 BCL2A1 BCL2 BAG6 CHEK2
GO:0051098	regulation of binding	12/117	369/18493	0	JUN PPP1CA TGFB1 SMAD3 BCL2 ANXA2 EIF3E GSK3B PARP1 FKBP1A MARK3 CAV1
GO:0043900	regulation of multi-organism process	12/117	371/18493	0	JUN SFTPD PPIA BCL2 TMPRSS2 ANXA2 CHMP4B PABPC1 PPIH CHMP2B DDX5 CAV1

ID	Description	GeneRatio	BgRatio	p.adjust	geneID
GO:0046902	regulation of mitochondrial membrane permeability	6/117	73/18493	0	STAT3 BCL2L1 BCL2 NMT1 GSK3A GSK3B
GO:0061614	pri-miRNA transcription by RNA polymerase II	5/117	44/18493	0.001	JUN STAT3 TGFB1 SMAD3 DDX5
GO:1903409	reactive oxygen species biosynthetic process	7/117	115/18493	0.001	STAT3 SMAD3 COX2 HSPD1 GBF1 DDAH2 CAV1
GO:0043902	positive regulation of multi-organism process	8/117	162/18493	0.001	JUN PPIA TMPRSS2 ANXA2 CHMP4B PABPC1 PPIH CHMP2B
GO:0006446	regulation of translational initiation	6/117	77/18493	0.001	PPP1CA RPL13A EIF3E NPM1 DDX1 MIF4GD
GO:0030193	regulation of blood coagulation	6/117	77/18493	0.001	CD9 CEACAM1 ANXA2 SERPING1 VKORC1 CAV1
GO:1900046	regulation of hemostasis	6/117	77/18493	0.001	CD9 CEACAM1 ANXA2 SERPING1 VKORC1 CAV1
GO:0019079	viral genome replication	7/117	117/18493	0.001	CD209 CLEC4M PPIA BCL2 PABPC1 PPIH DDX5
GO:0006457	protein folding	9/117	218/18493	0.001	PPIA DNAJB1 HSPD1 HSPA9 FKBP1A PPIG PPIH PFDN5 SGTA
GO:0035196	production of miRNAs involved in gene silencing by miRNA	5/117	46/18493	0.001	TGFB1 SMAD3 PRKRA HNRNPA2B1 DDX5
GO:0008637	apoptotic mitochondrial changes	7/117	121/18493	0.001	JUN BCL2L1 BCL2 HSPD1 NMT1 GSK3A GSK3B
GO:0090559	regulation of membrane permeability	6/117	81/18493	0.001	STAT3 BCL2L1 BCL2 NMT1 GSK3A GSK3B
GO:0050818	regulation of coagulation	6/117	82/18493	0.001	CD9 CEACAM1 ANXA2 SERPING1 VKORC1 CAV1
GO:0051656	establishment of organelle localization	13/117	491/18493	0.001	SCFD1 YKT6 KPNB1 STX5 XPO1 CHMP4B GBF1 GSK3B NPM1 CHMP2B SNAP47 TERF1 DCTN2
GO:0031050	dsRNA processing	5/117	49/18493	0.001	TGFB1 SMAD3 PRKRA HNRNPA2B1 DDX5
GO:0070918	production of small RNA involved in gene silencing by RNA	5/117	49/18493	0.001	TGFB1 SMAD3 PRKRA HNRNPA2B1 DDX5
GO:0045862	positive regulation of proteolysis	11/117	352/18493	0.001	PSMC2 PHB STAT3 SMAD3 HSPD1 SNX9 GSK3A GSK3B BAG6 CAV1 SGTA
GO:2000209	regulation of anoikis	4/117	24/18493	0.001	MCL1 BCL2 CHEK2 CAV1
GO:0006913	nucleocytoplasmic transport	11/117	357/18493	0.001	KPNB1 STAT3 XPO1 TGFB1 SMAD3 HNRNPA1 KPNA2 KPNA4 HNRNPA2B1 GSK3B NPM1
GO:0019054	modulation by virus of host process	4/117	25/18493	0.001	KPNB1 KPNA2 BCL2L1 KPNA4
GO:0051169	nuclear transport	11/117	360/18493	0.001	KPNB1 STAT3 XPO1 TGFB1 SMAD3 HNRNPA1 KPNA2 KPNA4 HNRNPA2B1 GSK3B NPM1

ID	Description	GeneRatio	BgRatio	p.adjust	geneID
GO:1901214	regulation of neuron death	10/117	296/18493	0.001	JUN STAT3 BCL2L1 MCL1 BCL2 HSPD1 CHMP4B NONO GSK3B PARP1
GO:0051099	positive regulation of binding	8/117	181/18493	0.001	TGFB1 ANXA2 EIF3E GSK3B PARP1 FKBP1A MARK3 CAV1
GO:0042026	protein refolding	4/117	26/18493	0.001	HSPD1 HSPA9 FKBP1A PPIG
GO:0061041	regulation of wound healing	7/117	134/18493	0.001	SMAD3 CD9 CEACAM1 ANXA2 SERPING1 VKORC1 CAV1
GO:1902895	positive regulation of pri-miRNA transcription by RNA polymerase II	4/117	27/18493	0.001	JUN STAT3 TGFB1 SMAD3
GO:0006402	mRNA catabolic process	10/117	307/18493	0.001	XPO1 SYNCRIP RPL13A EIF3E PABPC1 PABPC4 NPM1 RPL19 RPS20 DDX5
GO:0019062	virion attachment to host cell	3/117	10/18493	0.001	ACE2 CD209 CLEC4M
GO:0042110	T cell activation	12/117	451/18493	0.001	STAT3 TGFB1 SMAD3 CLEC4G CD209 SFTPD BCL2 DPP4 CEACAM1 HSPD1 FKBP1A CAV1
GO:1903426	regulation of reactive oxygen species biosynthetic process	6/117	94/18493	0.001	STAT3 SMAD3 COX2 HSPD1 DDAH2 CAV1
GO:2001244	positive regulation of intrinsic apoptotic signaling pathway	5/117	57/18493	0.001	BCL2L1 MCL1 BCL2 PRKRA CAV1
GO:0034248	regulation of cellular amide metabolic process	12/117	453/18493	0.001	STAT3 PPP1CA SYNCRIP RPL13A EIF3E GSK3A PABPC1 NPM1 NCL DDX1 MIF4GD EEF1A1
GO:0016050	vesicle organization	10/117	314/18493	0.001	SCFD1 STX5 BCL2 ANXA2 CHMP4B GBF1 HGS CHMP2B SNAP47 CAV1
GO:0044068	modulation by symbiont of host cellular process	4/117	29/18493	0.001	KPNB1 KPNA2 BCL2L1 KPNA4
GO:2001243	negative regulation of intrinsic apoptotic signaling pathway	6/117	96/18493	0.001	BCL2L1 BCL2L2 MCL1 BCL2A1 BCL2 NONO
GO:0090150	establishment of protein localization to membrane	10/117	317/18493	0.001	BCL2 RPL13A NMT1 CHMP4B SRP54 RPL19 BAG6 SNAP47 RPS20 SGTA
GO:0008380	RNA splicing	12/117	463/18493	0.001	HNRNPA1 ZCRB1 SYNCRIP PTBP1 HNRNPA2B1 HNRNPA3 NONO PABPC1 DDX1 PPIG PPIH DDX5
GO:0090307	mitotic spindle assembly	5/117	60/18493	0.001	KPNB1 CHMP4B KIF11 CHMP2B CHEK2
GO:0070972	protein localization to endoplasmic reticulum	7/117	146/18493	0.002	RPL13A CHMP4B GBF1 SRP54 RPL19 RPS20 SGTA
GO:0002183	cytoplasmic translational initiation	4/117	31/18493	0.002	RPL13A EIF3E EIF3I EIF3F
GO:1901215	negative regulation of neuron death	8/117	201/18493	0.002	JUN STAT3 BCL2L1 BCL2 HSPD1 CHMP4B NONO GSK3B
GO:0061025	membrane fusion	7/117	149/18493	0.002	YKT6 STX5 PPIA CD9 ANXA2 CHMP2B SNAP47

ID	Description	GeneRatio	BgRatio	p.adjust	geneID
GO:1903311	regulation of mRNA metabolic process	9/117	264/18493	0.002	XPO1 HNRNPA1 SYNCRIP PTBP1 HNRNPA2B1 PABPC1 PABPC4 NPM1 DDX5
GO:1901030	positive regulation of mitochondrial outer membrane permeabilization involved in apoptotic signaling pathway	4/117	32/18493	0.002	BCL2 NMT1 GSK3A GSK3B
GO:1901800	positive regulation of proteasomal protein catabolic process	6/117	103/18493	0.002	PSMC2 GSK3A GSK3B BAG6 CAV1 SGTA
GO:0070997	neuron death	10/117	335/18493	0.002	JUN STAT3 BCL2L1 MCL1 BCL2 HSPD1 CHMP4B NONO GSK3B PARP1
GO:0034504	protein localization to nucleus	9/117	269/18493	0.002	KPNB1 STAT3 XPO1 TGFB1 SMAD3 KPNA2 KPNA4 GSK3B PARP1
GO:0006401	RNA catabolic process	10/117	340/18493	0.002	XPO1 SYNCRIP RPL13A EIF3E PABPC1 PABPC4 NPM1 RPL19 RPS20 DDX5
GO:0043276	anoikis	4/117	34/18493	0.002	MCL1 BCL2 CHEK2 CAV1
GO:0045732	positive regulation of protein catabolic process	8/117	212/18493	0.002	PSMC2 STX5 SNX9 GSK3A GSK3B BAG6 CAV1 SGTA
GO:0022618	ribonucleoprotein complex assembly	9/117	275/18493	0.002	RSL24D1 RPL13A EIF3E EIF3I EIF3F NPM1 G3BP1 G3BP2 DDX1
GO:0007052	mitotic spindle organization	6/117	108/18493	0.002	KPNB1 CHMP4B KIF11 CHMP2B CHEK2 DCTN2
GO:0032885	regulation of polysaccharide biosynthetic process	4/117	35/18493	0.002	PPP1CA TGFB1 GSK3A GSK3B
GO:1903034	regulation of response to wounding	7/117	160/18493	0.002	SMAD3 CD9 CEACAM1 ANXA2 SERPING1 VKORC1 CAV1
GO:0001732	formation of cytoplasmic translation initiation complex	3/117	14/18493	0.003	EIF3E EIF3I EIF3F
GO:1904294	positive regulation of ERAD pathway	3/117	14/18493	0.003	BAG6 CAV1 SGTA
GO:1902893	regulation of pri-miRNA transcription by RNA polymerase II	4/117	37/18493	0.003	JUN STAT3 TGFB1 SMAD3
GO:0062012	regulation of small molecule metabolic process	10/117	355/18493	0.003	KPNB1 STAT3 PPP1CA TGFB1 COX2 CEACAM1 GSK3A GSK3B PARP1 CAV1
GO:0061045	negative regulation of wound healing	5/117	72/18493	0.003	SMAD3 CD9 CEACAM1 ANXA2 SERPING1
GO:0071826	ribonucleoprotein complex subunit organization	9/117	289/18493	0.003	RSL24D1 RPL13A EIF3E EIF3I EIF3F NPM1 G3BP1 G3BP2 DDX1
GO:0045047	protein targeting to ER	6/117	117/18493	0.003	RPL13A CHMP4B SRP54 RPL19 RPS20 SGTA
GO:1903052	positive regulation of proteolysis involved in cellular protein catabolic process	6/117	118/18493	0.003	PSMC2 GSK3A GSK3B BAG6 CAV1 SGTA
GO:0000413	protein peptidyl-prolyl isomerization	4/117	39/18493	0.003	PPIA FKBP1A PPIG PPIH

ID	Description	GeneRatio	BgRatio	p.adjust	geneID
GO:0007098	centrosome cycle	6/117	119/18493	0.003	XPO1 ATF5 CHMP4B KIF11 NPM1 CHMP2B
GO:0006458	'de novo' protein folding	4/117	40/18493	0.003	DNAJB1 HSPD1 HSPA9 FKBP1A
GO:0032881	regulation of polysaccharide metabolic process	4/117	40/18493	0.003	PPP1CA TGFB1 GSK3A GSK3B
GO:0032434	regulation of proteasomal ubiquitin-dependent protein catabolic process	6/117	121/18493	0.003	GSK3A GSK3B RYBP BAG6 CAV1 SGTA
GO:0072599	establishment of protein localization to endoplasmic reticulum	6/117	121/18493	0.003	RPL13A CHMP4B SRP54 RPL19 RPS20 SGTA
GO:0070507	regulation of microtubule cytoskeleton organization	7/117	175/18493	0.003	XPO1 ATF5 CHMP4B KIF11 GSK3B NPM1 CHMP2B
GO:1901028	regulation of mitochondrial outer membrane permeabilization involved in apoptotic signaling pathway	4/117	41/18493	0.004	BCL2 NMT1 GSK3A GSK3B
GO:0000377	RNA splicing, via transesterification reactions with bulged adenosine as nucleophile	10/117	374/18493	0.004	HNRNPA1 ZCRB1 SYNCRIP PTBP1 HNRNPA2B1 HNRNPA3 PABPC1 DDX1 PPIH DDX5
GO:0000398	mRNA splicing, via spliceosome	10/117	374/18493	0.004	HNRNPA1 ZCRB1 SYNCRIP PTBP1 HNRNPA2B1 HNRNPA3 PABPC1 DDX1 PPIH DDX5
GO:0051170	import into nucleus	7/117	178/18493	0.004	KPNB1 STAT3 TGFB1 SMAD3 HNRNPA1 KPNA2 KPNA4
GO:0010498	proteasomal protein catabolic process	11/117	452/18493	0.004	PSMC2 SKP2 PSMD1 GSK3A GSK3B RYBP BAG6 PSMA2 CAV1 UBE2I SGTA
GO:0010824	regulation of centrosome duplication	4/117	42/18493	0.004	XPO1 CHMP4B NPM1 CHMP2B
GO:0000375	RNA splicing, via transesterification reactions	10/117	377/18493	0.004	HNRNPA1 ZCRB1 SYNCRIP PTBP1 HNRNPA2B1 HNRNPA3 PABPC1 DDX1 PPIH DDX5
GO:0061136	regulation of proteasomal protein catabolic process	7/117	179/18493	0.004	PSMC2 GSK3A GSK3B RYBP BAG6 CAV1 SGTA
GO:0033143	regulation of intracellular steroid hormone receptor signaling pathway	5/117	79/18493	0.004	PHB SKP2 PARP1 DDX5 UBE2I
GO:1903362	regulation of cellular protein catabolic process	8/117	242/18493	0.004	PSMC2 ANXA2 GSK3A GSK3B RYBP BAG6 CAV1 SGTA
GO:0045912	negative regulation of carbohydrate metabolic process	4/117	43/18493	0.004	STAT3 TGFB1 GSK3A GSK3B
GO:0033145	positive regulation of intracellular steroid hormone receptor signaling pathway	3/117	17/18493	0.004	SKP2 PARP1 UBE2I
GO:2000811	negative regulation of anoikis	3/117	17/18493	0.004	MCL1 BCL2 CAV1
GO:0042177	negative regulation of protein catabolic process	6/117	128/18493	0.004	PHB SMAD3 ANXA2 RYBP BAG6 SGTA
GO:0043112	receptor metabolic process	7/117	184/18493	0.004	TGFB1 ACE2 CD9 CEACAM1 ANXA2 CAMLG CAV1

ID	Description	GeneRatio	BgRatio	p.adjust	geneID
GO:1902850	microtubule cytoskeleton organization involved in mitosis	6/117	129/18493	0.004	KPNB1 CHMP4B KIF11 CHMP2B CHEK2 DCTN2
GO:0009615	response to virus	9/117	315/18493	0.004	IRF3 BCL2L1 BCL2 SKP2 PRKRA IKBKB GBF1 DDX1 PSMA2
GO:0006914	autophagy	11/117	466/18493	0.004	SCFD1 TFEB ACBD5 MCL1 BCL2 CHMP4B GSK3A HGS CHMP2B EEF1A1 ATP6V1G1
GO:0061919	process utilizing autophagic mechanism	11/117	466/18493	0.004	SCFD1 TFEB ACBD5 MCL1 BCL2 CHMP4B GSK3A HGS CHMP2B EEF1A1 ATP6V1G1
GO:0043161	proteasome-mediated ubiquitin-dependent protein catabolic process	10/117	389/18493	0.004	SKP2 PSMD1 GSK3A GSK3B RYBP BAG6 PSMA2 CAV1 UBE2I SGTA
GO:0007006	mitochondrial membrane organization	6/117	131/18493	0.004	STAT3 BCL2L1 BCL2 NMT1 GSK3A GSK3B
GO:0008631	intrinsic apoptotic signaling pathway in response to oxidative stress	4/117	45/18493	0.004	MCL1 BCL2 NONO PARP1
GO:1903035	negative regulation of response to wounding	5/117	84/18493	0.004	SMAD3 CD9 CEACAM1 ANXA2 SERPING1
GO:0031023	microtubule organizing center organization	6/117	133/18493	0.005	XPO1 ATF5 CHMP4B KIF11 NPM1 CHMP2B
GO:2001239	regulation of extrinsic apoptotic signaling pathway in absence of ligand	4/117	46/18493	0.005	PPP1CA BCL2L1 MCL1 BCL2
GO:0046677	response to antibiotic	9/117	323/18493	0.005	PHB JUN STAT3 HNRNPA1 BCL2L1 BCL2 KPNA4 HSPD1 VKORC1
GO:0035722	interleukin-12-mediated signaling pathway	4/117	47/18493	0.005	PPIA ANXA2 HNRNPA2B1 HSPA9
GO:0051205	protein insertion into membrane	4/117	47/18493	0.005	BCL2 NMT1 BAG6 SGTA
GO:0007159	leukocyte cell-cell adhesion	9/117	327/18493	0.005	TGFB1 CLEC4G CD209 CLEC4M SFTPD DPP4 CEACAM1 HSPD1 CAV1
GO:0001889	liver development	6/117	137/18493	0.005	JUN TGFB1 SMAD3 CEACAM1 ACO2 RPL19
GO:0006417	regulation of translation	10/117	402/18493	0.005	STAT3 PPP1CA SYNCRIP RPL13A EIF3E PABPC1 NPM1 NCL DDX1 MIF4GD
GO:0035304	regulation of protein dephosphorylation	6/117	138/18493	0.005	TGFB1 IKBKB PTBP1 GSK3B FKBP1A RCAN3
GO:1903364	positive regulation of cellular protein catabolic process	6/117	138/18493	0.005	PSMC2 GSK3A GSK3B BAG6 CAV1 SGTA
GO:0022613	ribonucleoprotein complex biogenesis	11/117	485/18493	0.005	RSL24D1 XPO1 RPL13A EIF3E EIF3I EIF3F NPM1 G3BP1 G3BP2 DDX1 LAS1L
GO:0071349	cellular response to interleukin-12	4/117	49/18493	0.005	PPIA ANXA2 HNRNPA2B1 HSPA9

ID	Description	GeneRatio	BgRatio	p.adjust	geneID
GO:0009896	positive regulation of catabolic process	10/117	407/18493	0.005	PSMC2 TFEB STX5 SNX9 GSK3A GSK3B PABPC1 BAG6 CAV1 SGTA
GO:0061008	hepaticobiliary system development	6/117	140/18493	0.005	JUN TGFB1 SMAD3 CEACAM1 ACO2 RPL19
GO:0045069	regulation of viral genome replication	5/117	90/18493	0.005	PPIA BCL2 PABPC1 PPIH DDX5
GO:0051236	establishment of RNA localization	7/117	198/18493	0.005	XPO1 HNRNPA1 HNRNPA2B1 HNRNPA3 NPM1 G3BP2 TERF1
GO:0070671	response to interleukin-12	4/117	50/18493	0.006	PPIA ANXA2 HNRNPA2B1 HSPA9
GO:0043523	regulation of neuron apoptotic process	7/117	202/18493	0.006	JUN BCL2L1 MCL1 BCL2 HSPD1 NONO PARP1
GO:0071359	cellular response to dsRNA	3/117	21/18493	0.006	IRF3 NPM1 CAV1
GO:1904886	beta-catenin destruction complex disassembly	3/117	21/18493	0.006	PPP1CA GSK3B CAV1
GO:0097345	mitochondrial outer membrane permeabilization	4/117	51/18493	0.006	BCL2 NMT1 GSK3A GSK3B
GO:0002181	cytoplasmic translation	5/117	93/18493	0.006	RPL13A EIF3E EIF3I EIF3F RPL19
GO:0032886	regulation of microtubule-based process	7/117	204/18493	0.006	XPO1 ATF5 CHMP4B KIF11 GSK3B NPM1 CHMP2B
GO:0030195	negative regulation of blood coagulation	4/117	52/18493	0.006	CD9 CEACAM1 ANXA2 SERPING1
GO:1900047	negative regulation of hemostasis	4/117	52/18493	0.006	CD9 CEACAM1 ANXA2 SERPING1
GO:0032092	positive regulation of protein binding	5/117	94/18493	0.006	ANXA2 GSK3B FKBP1A MARK3 CAV1
GO:0035303	regulation of dephosphorylation	7/117	206/18493	0.006	TGFB1 SMAD3 IKBKB PTBP1 GSK3B FKBP1A RCAN3
GO:2000058	regulation of ubiquitin-dependent protein catabolic process	6/117	147/18493	0.006	GSK3A GSK3B RYBP BAG6 CAV1 SGTA
GO:2000637	positive regulation of gene silencing by miRNA	3/117	22/18493	0.006	STAT3 TGFB1 DDX5
GO:0010332	response to gamma radiation	4/117	53/18493	0.006	BCL2L1 BCL2 PARP1 CHEK2
GO:0043331	response to dsRNA	4/117	53/18493	0.006	IRF3 NPM1 DDX1 CAV1
GO:0016197	endosomal transport	7/117	208/18493	0.007	YKT6 STX5 CHMP4B GBF1 SNX9 HGS CHMP2B
GO:0031331	positive regulation of cellular catabolic process	9/117	347/18493	0.007	PSMC2 TFEB SNX9 GSK3A GSK3B PABPC1 BAG6 CAV1 SGTA
GO:1903050	regulation of proteolysis involved in cellular protein catabolic process	7/117	209/18493	0.007	PSMC2 GSK3A GSK3B RYBP BAG6 CAV1 SGTA
GO:0062014	negative regulation of small molecule metabolic process	5/117	97/18493	0.007	STAT3 TGFB1 CEACAM1 GSK3A PARP1

ID	Description	GeneRatio	BgRatio	p.adjust	geneID
GO:2000379	positive regulation of reactive oxygen species metabolic process	5/117	98/18493	0.007	TGFB1 SMAD3 COX2 ACE2 DDAH2
GO:0030856	regulation of epithelial cell differentiation	6/117	151/18493	0.007	IKBKB CEACAM1 GSK3A GSK3B H2AFY2 CAV1
GO:0060148	positive regulation of posttranscriptional gene silencing	3/117	23/18493	0.007	STAT3 TGFB1 DDX5
GO:0017038	protein import	7/117	214/18493	0.007	KPNB1 STAT3 TGFB1 SMAD3 KPNA2 KPNA4 HSPD1
GO:0018208	peptidyl-proline modification	4/117	56/18493	0.007	PPIA FKBP1A PPIG PPIH
GO:0050819	negative regulation of coagulation	4/117	56/18493	0.007	CD9 CEACAM1 ANXA2 SERPING1
GO:0048732	gland development	10/117	434/18493	0.007	STAT5A JUN TGFB1 SMAD3 COX2 BCL2 CEACAM1 ACO2 RPL19 CAV1
GO:1901653	cellular response to peptide	9/117	356/18493	0.007	STAT5A STAT3 TGFB1 CEACAM1 GSK3A GSK3B PARP1 CAV1 ATP6V1G1
GO:1903203	regulation of oxidative stress-induced neuron death	3/117	24/18493	0.008	MCL1 NONO PARP1
GO:0045216	cell-cell junction organization	6/117	155/18493	0.008	TGFB1 SMAD3 ACE2 CD9 IKBKB CAV1
GO:0043393	regulation of protein binding	7/117	217/18493	0.008	PPP1CA BCL2 ANXA2 GSK3B FKBP1A MARK3 CAV1
GO:1902110	positive regulation of mitochondrial membrane permeability involved in apoptotic process	4/117	57/18493	0.008	BCL2 NMT1 GSK3A GSK3B
GO:0006606	protein import into nucleus	6/117	157/18493	0.008	KPNB1 STAT3 TGFB1 SMAD3 KPNA2 KPNA4
GO:2000378	negative regulation of reactive oxygen species metabolic process	4/117	58/18493	0.008	STAT3 BCL2 HSPD1 CAV1
GO:0036475	neuron death in response to oxidative stress	3/117	25/18493	0.008	MCL1 NONO PARP1
GO:0045088	regulation of innate immune response	9/117	365/18493	0.009	CD209 IRF3 SFTPD IKBKB CEACAM1 HSPD1 NONO SERPING1 CAV1
GO:1902686	mitochondrial outer membrane permeabilization involved in programmed cell death	4/117	59/18493	0.009	BCL2 NMT1 GSK3A GSK3B
GO:0051225	spindle assembly	5/117	105/18493	0.009	KPNB1 CHMP4B KIF11 CHMP2B CHEK2
GO:1903037	regulation of leukocyte cell-cell adhesion	8/117	293/18493	0.009	TGFB1 CLEC4G CD209 SFTPD DPP4 CEACAM1 HSPD1 CAV1
GO:0061077	chaperone-mediated protein folding	4/117	60/18493	0.009	DNAJB1 HSPA9 FKBP1A SGTA
GO:0035794	positive regulation of mitochondrial membrane permeability	4/117	61/18493	0.01	BCL2 NMT1 GSK3A GSK3B

ID	Description	GeneRatio	BgRatio	p.adjust	geneID
GO:1904356	regulation of telomere maintenance via telomere lengthening	4/117	61/18493	0.01	HNRNPA1 HNRNPA2B1 PARP1 TERF1

2 HCOV-CLUSTER_SET

2.1 GO ENRICHMENT

GO Enrichment Analysis after FDR control of a gene set.

MOLECULAR FUNCTION

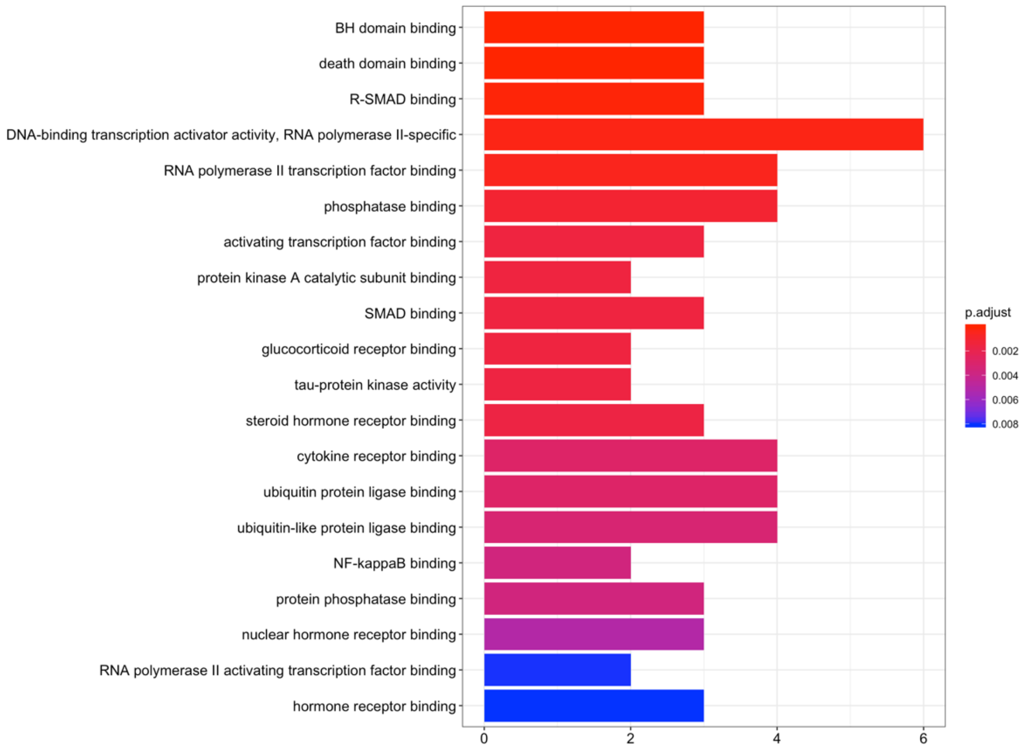

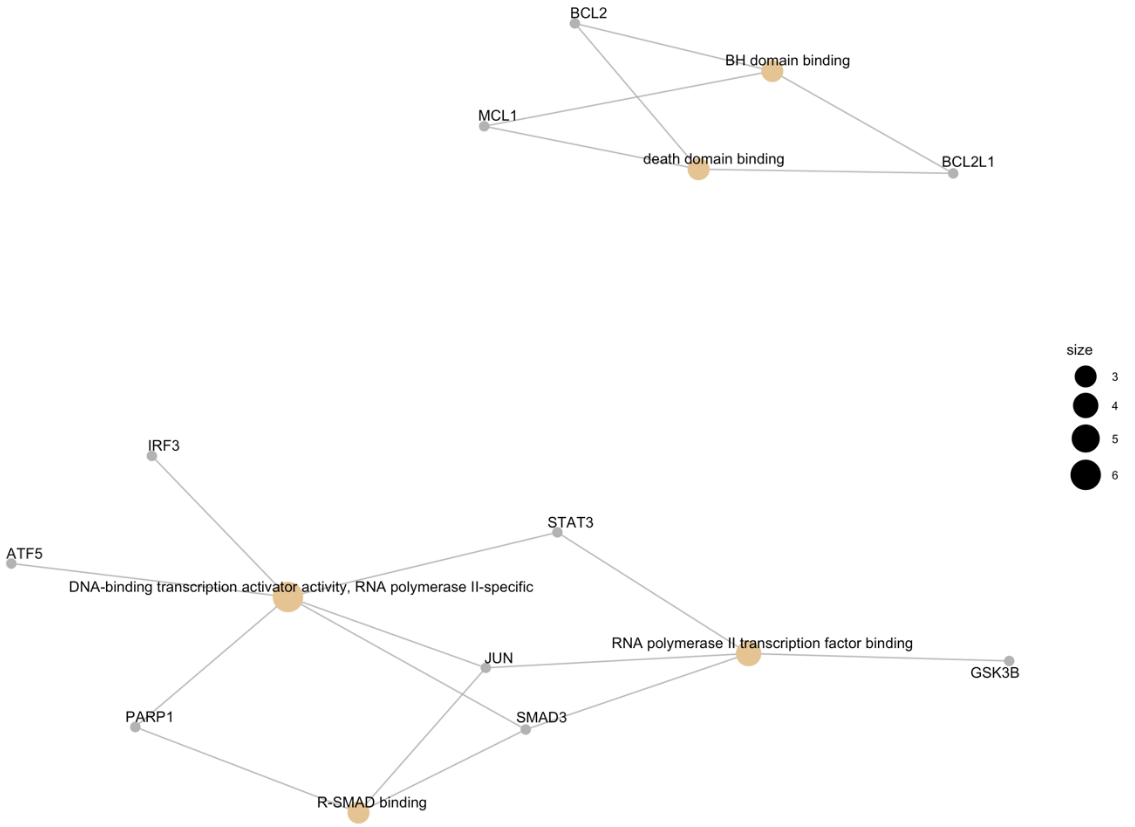

CELLULAR COMPONENT

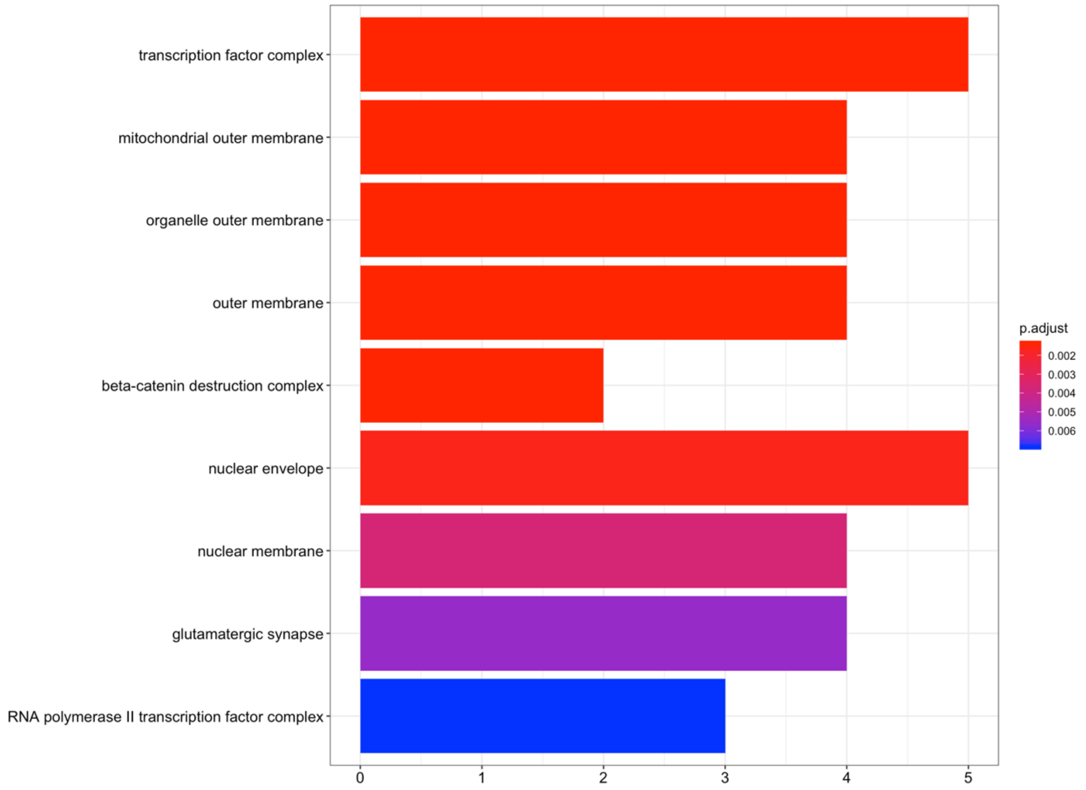

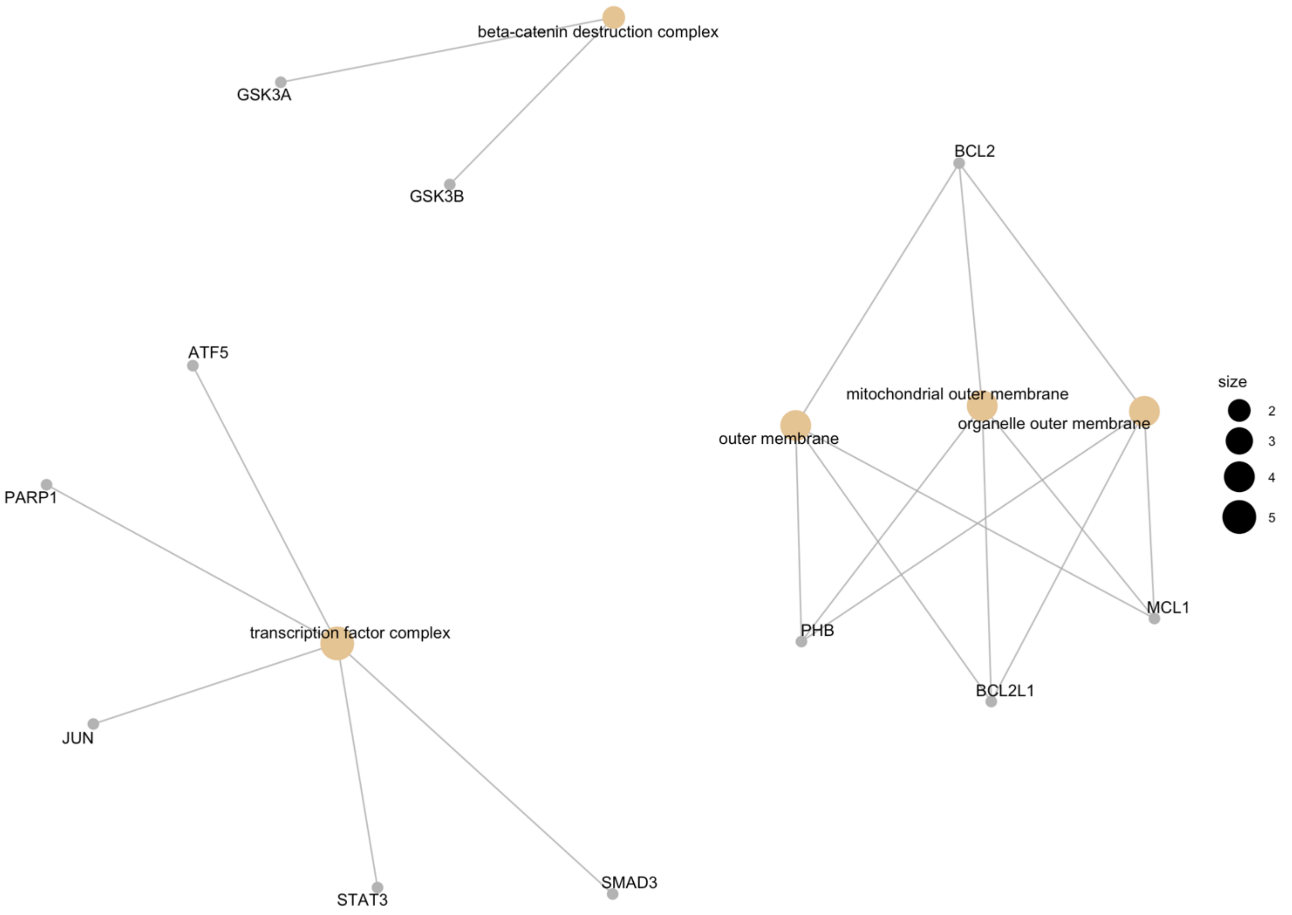

BIOLOGICAL PROCESS

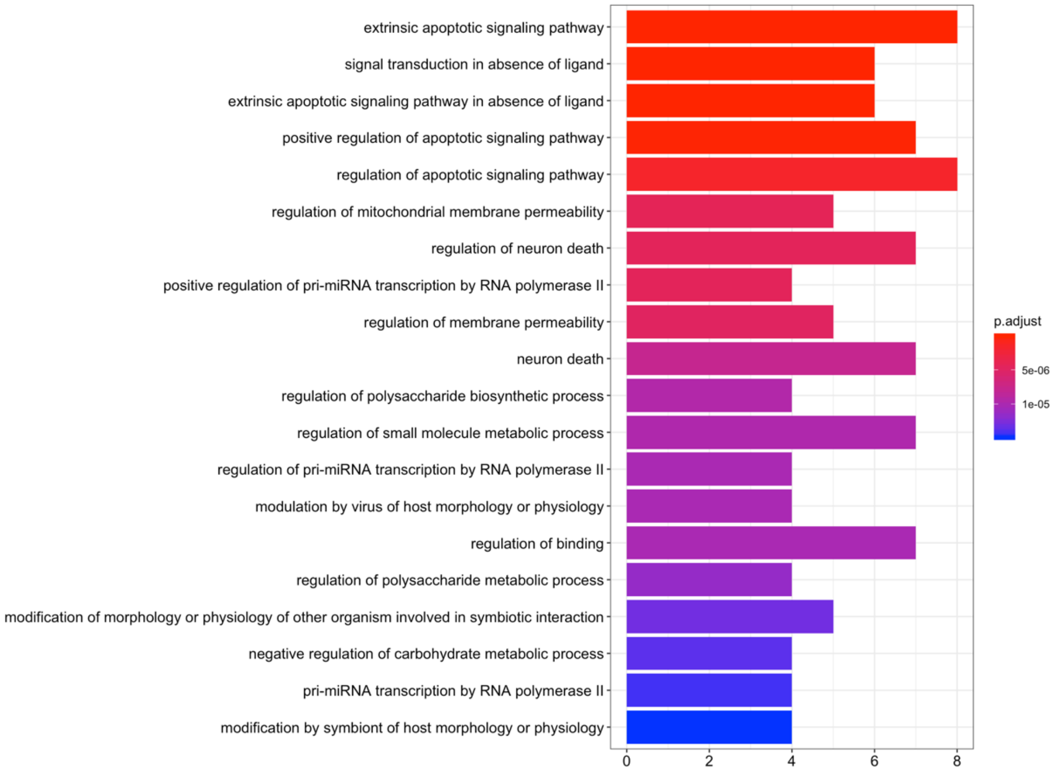

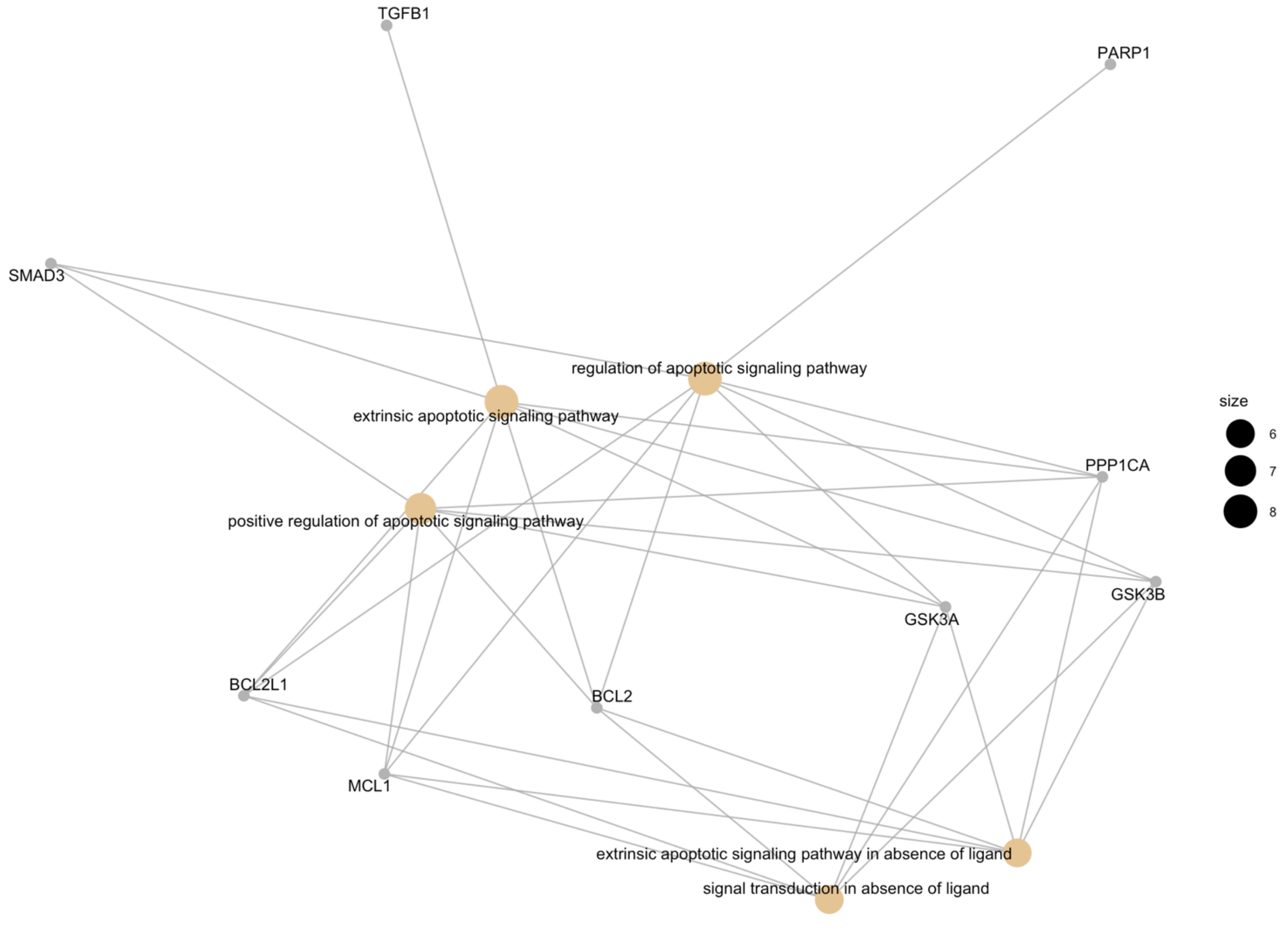

2.2 TABLES ENRICHMENT

2.2.1 MOLECULAR FUNCTION

ID	Description	GeneRatio	BgRatio	p.adjust	geneID
GO:0051400	BH domain binding	3/21	10/17632	0	BCL2L1 MCL1 BCL2
GO:0070513	death domain binding	3/21	10/17632	0	BCL2L1 MCL1 BCL2
GO:0070412	R-SMAD binding	3/21	23/17632	0	JUN SMAD3 PARP1
GO:0001228	DNA-binding transcription activator activity, RNA polymerase II-specific	6/21	449/17632	0	JUN STAT3 SMAD3 ATF5 IRF3 PARP1
GO:0001085	RNA polymerase II transcription factor binding	4/21	140/17632	0.001	JUN STAT3 SMAD3 GSK3B
GO:0019902	phosphatase binding	4/21	178/17632	0.001	STAT3 PPP1CA SMAD3 BCL2
GO:0033613	activating transcription factor binding	3/21	76/17632	0.001	JUN SMAD3 NPM1
GO:0034236	protein kinase A catalytic subunit binding	2/21	13/17632	0.001	GSK3A GSK3B
GO:0046332	SMAD binding	3/21	80/17632	0.001	JUN SMAD3 PARP1
GO:0035259	glucocorticoid receptor binding	2/21	14/17632	0.001	STAT3 SMAD3
GO:0050321	tau-protein kinase activity	2/21	15/17632	0.002	GSK3A GSK3B
GO:0035258	steroid hormone receptor binding	3/21	90/17632	0.002	STAT3 SMAD3 PARP1
GO:0005126	cytokine receptor binding	4/21	281/17632	0.003	STAT3 TGFB1 SMAD3 IL6
GO:0031625	ubiquitin protein ligase binding	4/21	286/17632	0.003	JUN SMAD3 BCL2 GSK3B
GO:0044389	ubiquitin-like protein ligase binding	4/21	301/17632	0.003	JUN SMAD3 BCL2 GSK3B
GO:0051059	NF-kappaB binding	2/21	28/17632	0.004	GSK3B NPM1
GO:0019903	protein phosphatase binding	3/21	133/17632	0.004	STAT3 PPP1CA BCL2
GO:0035257	nuclear hormone receptor binding	3/21	152/17632	0.005	STAT3 SMAD3 PARP1
GO:0001102	RNA polymerase II activating transcription factor binding	2/21	44/17632	0.008	JUN SMAD3
GO:0051427	hormone receptor binding	3/21	185/17632	0.008	STAT3 SMAD3 PARP1
GO:0051018	protein kinase A binding	2/21	48/17632	0.009	GSK3A GSK3B
GO:0005160	transforming growth factor beta receptor binding	2/21	51/17632	0.009	TGFB1 SMAD3

2.2.2 CELLULAR COMPONENT

ID	Description	GeneRatio	BgRatio	p.adjust	geneID
GO:0005667	transcription factor complex	5/21	356/19659	0.001	JUN STAT3 SMAD3 ATF5 PARP1
GO:0005741	mitochondrial outer membrane	4/21	180/19659	0.001	PHB BCL2L1 MCL1 BCL2
GO:0031968	organelle outer membrane	4/21	204/19659	0.001	PHB BCL2L1 MCL1 BCL2
GO:0019867	outer membrane	4/21	206/19659	0.001	PHB BCL2L1 MCL1 BCL2
GO:0030877	beta-catenin destruction complex	2/21	12/19659	0.001	GSK3A GSK3B
GO:0005635	nuclear envelope	5/21	461/19659	0.002	KPNB1 SMAD3 BCL2L1 BCL2 PARP1
GO:0031965	nuclear membrane	4/21	302/19659	0.004	KPNB1 SMAD3 BCL2L1 BCL2
GO:0098978	glutamatergic synapse	4/21	350/19659	0.006	PHB STAT3 PPP1CA GSK3B
GO:0090575	RNA polymerase II transcription factor complex	3/21	161/19659	0.007	JUN STAT3 SMAD3

2.2.3 BIOLOGICAL PROCESS

ID	Description	GeneRatio	BgRatio	p.adjust	geneID
GO:0097191	extrinsic apoptotic signaling pathway	8/20	224/18493	0	PPP1CA TGFB1 SMAD3 BCL2L1 MCL1 BCL2 GSK3A GSK3B
GO:0038034	signal transduction in absence of ligand	6/20	71/18493	0	PPP1CA BCL2L1 MCL1 BCL2 GSK3A GSK3B
GO:0097192	extrinsic apoptotic signaling pathway in absence of ligand	6/20	71/18493	0	PPP1CA BCL2L1 MCL1 BCL2 GSK3A GSK3B
GO:2001235	positive regulation of apoptotic signaling pathway	7/20	170/18493	0	PPP1CA SMAD3 BCL2L1 MCL1 BCL2 GSK3A GSK3B
GO:2001233	regulation of apoptotic signaling pathway	8/20	391/18493	0	PPP1CA SMAD3 BCL2L1 MCL1 BCL2 GSK3A GSK3B PARP1
GO:0046902	regulation of mitochondrial membrane permeability	5/20	73/18493	0	STAT3 BCL2L1 BCL2 GSK3A GSK3B
GO:1901214	regulation of neuron death	7/20	296/18493	0	JUN STAT3 BCL2L1 MCL1 BCL2 GSK3B PARP1
GO:1902895	positive regulation of pri-miRNA transcription by RNA polymerase II	4/20	27/18493	0	JUN STAT3 TGFB1 SMAD3
GO:0090559	regulation of membrane permeability	5/20	81/18493	0	STAT3 BCL2L1 BCL2 GSK3A GSK3B

ID	Description	GeneRatio	BgRatio	p.adjust	geneID
GO:0070997	neuron death	7/20	335/18493	0	JUN STAT3 BCL2L1 MCL1 BCL2 GSK3B PARP1
GO:0032885	regulation of polysaccharide biosynthetic process	4/20	35/18493	0	PPP1CA TGFB1 GSK3A GSK3B
GO:0062012	regulation of small molecule metabolic process	7/20	355/18493	0	KPNB1 STAT3 PPP1CA TGFB1 GSK3A GSK3B PARP1
GO:1902893	regulation of pri-miRNA transcription by RNA polymerase II	4/20	37/18493	0	JUN STAT3 TGFB1 SMAD3
GO:0019048	modulation by virus of host morphology or physiology	4/20	38/18493	0	KPNB1 TGFB1 SMAD3 BCL2L1
GO:0051098	regulation of binding	7/20	369/18493	0	JUN PPP1CA TGFB1 SMAD3 BCL2 GSK3B PARP1
GO:0032881	regulation of polysaccharide metabolic process	4/20	40/18493	0	PPP1CA TGFB1 GSK3A GSK3B
GO:0051817	modification of morphology or physiology of other organism involved in symbiotic interaction	5/20	112/18493	0	KPNB1 JUN TGFB1 SMAD3 BCL2L1
GO:0045912	negative regulation of carbohydrate metabolic process	4/20	43/18493	0	STAT3 TGFB1 GSK3A GSK3B
GO:0061614	pri-miRNA transcription by RNA polymerase II	4/20	44/18493	0	JUN STAT3 TGFB1 SMAD3
GO:0044003	modification by symbiont of host morphology or physiology	4/20	46/18493	0	KPNB1 TGFB1 SMAD3 BCL2L1
GO:2001239	regulation of extrinsic apoptotic signaling pathway in absence of ligand	4/20	46/18493	0	PPP1CA BCL2L1 MCL1 BCL2
GO:0008637	apoptotic mitochondrial changes	5/20	121/18493	0	JUN BCL2L1 BCL2 GSK3A GSK3B
GO:1904951	positive regulation of establishment of protein localization	7/20	437/18493	0	TGFB1 SMAD3 IRF3 BCL2 GSK3A GSK3B IL6
GO:0007006	mitochondrial membrane organization	5/20	131/18493	0	STAT3 BCL2L1 BCL2 GSK3A GSK3B
GO:0009314	response to radiation	7/20	443/18493	0	JUN PPP1CA TGFB1 BCL2L1 BCL2 NPM1 PARP1
GO:0034504	protein localization to nucleus	6/20	269/18493	0	KPNB1 STAT3 TGFB1 SMAD3 GSK3B PARP1
GO:0007183	SMAD protein complex assembly	3/20	13/18493	0	TGFB1 SMAD3 PARP1
GO:0009895	negative regulation of catabolic process	6/20	287/18493	0	PHB STAT3 SMAD3 MCL1 BCL2 GSK3A
GO:1901652	response to peptide	7/20	481/18493	0	STAT5A PHB STAT3 TGFB1 GSK3A GSK3B PARP1
GO:0035821	modification of morphology or physiology of other organism	5/20	161/18493	0	KPNB1 JUN TGFB1 SMAD3 BCL2L1

ID	Description	GeneRatio	BgRatio	p.adjust	geneID
GO:0006109	regulation of carbohydrate metabolic process	5/20	162/18493	0	STAT3 PPP1CA TGFB1 GSK3A GSK3B
GO:1903829	positive regulation of cellular protein localization	6/20	318/18493	0	TGFB1 SMAD3 BCL2 GSK3A GSK3B PARP1
GO:0060395	SMAD protein signal transduction	4/20	69/18493	0	JUN TGFB1 SMAD3 PARP1
GO:0046677	response to antibiotic	6/20	323/18493	0	PHB JUN STAT3 BCL2L1 BCL2 IL6
GO:0010721	negative regulation of cell development	6/20	326/18493	0	STAT3 TGFB1 BCL2 GSK3A GSK3B IL6
GO:0002363	alpha-beta T cell lineage commitment	3/20	19/18493	0	STAT3 BCL2 IL6
GO:0000271	polysaccharide biosynthetic process	4/20	74/18493	0	PPP1CA TGFB1 GSK3A GSK3B
GO:0030308	negative regulation of cell growth	5/20	184/18493	0	PHB TGFB1 SMAD3 BCL2 GSK3A
GO:0043369	CD4-positive or CD8-positive, alpha-beta T cell lineage commitment	3/20	21/18493	0	STAT3 BCL2 IL6
GO:1901653	cellular response to peptide	6/20	356/18493	0	STAT5A STAT3 TGFB1 GSK3A GSK3B PARP1
GO:0006913	nucleocytoplasmic transport	6/20	357/18493	0	KPNB1 STAT3 TGFB1 SMAD3 GSK3B NPM1
GO:1901216	positive regulation of neuron death	4/20	81/18493	0	JUN MCL1 GSK3B PARP1
GO:0051169	nuclear transport	6/20	360/18493	0	KPNB1 STAT3 TGFB1 SMAD3 GSK3B NPM1
GO:1901215	negative regulation of neuron death	5/20	201/18493	0	JUN STAT3 BCL2L1 BCL2 GSK3B
GO:0043523	regulation of neuron apoptotic process	5/20	202/18493	0	JUN BCL2L1 MCL1 BCL2 PARP1
GO:0048545	response to steroid hormone	6/20	390/18493	0	PHB TGFB1 BCL2 SKP2 PARP1 IL6
GO:0043255	regulation of carbohydrate biosynthetic process	4/20	92/18493	0	PPP1CA TGFB1 GSK3A GSK3B
GO:0002360	T cell lineage commitment	3/20	26/18493	0	STAT3 BCL2 IL6
GO:0051222	positive regulation of protein transport	6/20	402/18493	0	TGFB1 SMAD3 IRF3 GSK3A GSK3B IL6
GO:0062014	negative regulation of small molecule metabolic process	4/20	97/18493	0	STAT3 TGFB1 GSK3A PARP1
GO:0005979	regulation of glycogen biosynthetic process	3/20	28/18493	0	PPP1CA GSK3A GSK3B
GO:0010962	regulation of glucan biosynthetic process	3/20	28/18493	0	PPP1CA GSK3A GSK3B
GO:0001558	regulation of cell growth	6/20	416/18493	0	PHB TGFB1 SMAD3 BCL2 GSK3A GSK3B

ID	Description	GeneRatio	BgRatio	p.adjust	geneID
GO:0043434	response to peptide hormone	6/20	417/18493	0	STAT5A PHB STAT3 TGFB1 GSK3A PARP1
GO:0051402	neuron apoptotic process	5/20	230/18493	0	JUN BCL2L1 MCL1 BCL2 PARP1
GO:0097305	response to alcohol	5/20	231/18493	0	PHB STAT3 TGFB1 BCL2L1 PARP1
GO:0042391	regulation of membrane potential	6/20	424/18493	0	JUN BCL2L1 BCL2 GSK3A GSK3B PARP1
GO:0043467	regulation of generation of precursor metabolites and energy	4/20	105/18493	0	STAT3 PPP1CA GSK3A GSK3B
GO:0006839	mitochondrial transport	5/20	241/18493	0	STAT3 BCL2L1 BCL2 GSK3A GSK3B
GO:0005976	polysaccharide metabolic process	4/20	109/18493	0	PPP1CA TGFB1 GSK3A GSK3B
GO:0018107	peptidyl-threonine phosphorylation	4/20	109/18493	0	TGFB1 BCL2 GSK3A GSK3B
GO:1901030	positive regulation of mitochondrial outer membrane permeabilization involved in apoptotic signaling pathway	3/20	32/18493	0	BCL2 GSK3A GSK3B
GO:0033028	myeloid cell apoptotic process	3/20	33/18493	0	IRF3 BCL2 IL6
GO:0070873	regulation of glycogen metabolic process	3/20	33/18493	0	PPP1CA GSK3A GSK3B
GO:0071354	cellular response to interleukin-6	3/20	33/18493	0	PHB STAT3 IL6
GO:0018210	peptidyl-threonine modification	4/20	116/18493	0	TGFB1 BCL2 GSK3A GSK3B
GO:0045926	negative regulation of growth	5/20	260/18493	0	PHB TGFB1 SMAD3 BCL2 GSK3A
GO:0043368	positive T cell selection	3/20	35/18493	0	STAT3 BCL2 IL6
GO:1901099	negative regulation of signal transduction in absence of ligand	3/20	35/18493	0	BCL2L1 MCL1 BCL2
GO:2001240	negative regulation of extrinsic apoptotic signaling pathway in absence of ligand	3/20	35/18493	0	BCL2L1 MCL1 BCL2
GO:1900180	regulation of protein localization to nucleus	4/20	121/18493	0	TGFB1 SMAD3 GSK3B PARP1
GO:0070741	response to interleukin-6	3/20	37/18493	0	PHB STAT3 IL6
GO:0016049	cell growth	6/20	485/18493	0	PHB TGFB1 SMAD3 BCL2 GSK3A GSK3B
GO:0010677	negative regulation of cellular carbohydrate metabolic process	3/20	39/18493	0	STAT3 GSK3A GSK3B
GO:0035270	endocrine system development	4/20	128/18493	0	SMAD3 GSK3A GSK3B IL6
GO:1901028	regulation of mitochondrial outer membrane permeabilization involved in apoptotic signaling pathway	3/20	41/18493	0	BCL2 GSK3A GSK3B

ID	Description	GeneRatio	BgRatio	p.adjust	geneID
GO:0030522	intracellular receptor signaling pathway	5/20	288/18493	0	PHB STAT3 IRF3 SKP2 PARP1
GO:0034599	cellular response to oxidative stress	5/20	289/18493	0	JUN MCL1 BCL2 PARP1 IL6
GO:0010675	regulation of cellular carbohydrate metabolic process	4/20	134/18493	0	STAT3 PPP1CA GSK3A GSK3B
GO:0018209	peptidyl-serine modification	5/20	301/18493	0	TGFB1 BCL2 GSK3B PARP1 IL6
GO:0008631	intrinsic apoptotic signaling pathway in response to oxidative stress	3/20	45/18493	0	MCL1 BCL2 PARP1
GO:0031018	endocrine pancreas development	3/20	45/18493	0	GSK3A GSK3B IL6
GO:0071375	cellular response to peptide hormone stimulus	5/20	307/18493	0	STAT5A STAT3 TGFB1 GSK3A PARP1
GO:0005978	glycogen biosynthetic process	3/20	46/18493	0	PPP1CA GSK3A GSK3B
GO:0009250	glucan biosynthetic process	3/20	46/18493	0	PPP1CA GSK3A GSK3B
GO:0010212	response to ionizing radiation	4/20	146/18493	0	TGFB1 BCL2L1 BCL2 PARP1
GO:0007568	aging	5/20	311/18493	0	JUN STAT3 TGFB1 BCL2 NPM1
GO:0035264	multicellular organism growth	4/20	147/18493	0	STAT5A STAT3 ATF5 BCL2
GO:0009615	response to virus	5/20	315/18493	0	IRF3 BCL2L1 BCL2 SKP2 IL6
GO:0016202	regulation of striated muscle tissue development	4/20	149/18493	0	TGFB1 SMAD3 BCL2 GSK3A
GO:0043470	regulation of carbohydrate catabolic process	3/20	48/18493	0	STAT3 PPP1CA GSK3A
GO:0045058	T cell selection	3/20	48/18493	0	STAT3 BCL2 IL6
GO:1901861	regulation of muscle tissue development	4/20	151/18493	0	TGFB1 SMAD3 BCL2 GSK3A
GO:0045599	negative regulation of fat cell differentiation	3/20	49/18493	0	TGFB1 SMAD3 IL6
GO:0048634	regulation of muscle organ development	4/20	153/18493	0	TGFB1 SMAD3 BCL2 GSK3A
GO:2001236	regulation of extrinsic apoptotic signaling pathway	4/20	156/18493	0	PPP1CA BCL2L1 MCL1 BCL2
GO:0006606	protein import into nucleus	4/20	157/18493	0	KPNB1 STAT3 TGFB1 SMAD3
GO:0097345	mitochondrial outer membrane permeabilization	3/20	51/18493	0	BCL2 GSK3A GSK3B
GO:0090316	positive regulation of intracellular protein transport	4/20	158/18493	0	TGFB1 SMAD3 GSK3A GSK3B

ID	Description	GeneRatio	BgRatio	p.adjust	geneID
GO:0048638	regulation of developmental growth	5/20	334/18493	0	STAT5A STAT3 BCL2 GSK3A GSK3B
GO:2001242	regulation of intrinsic apoptotic signaling pathway	4/20	161/18493	0	BCL2L1 MCL1 BCL2 PARP1
GO:0010332	response to gamma radiation	3/20	53/18493	0	BCL2L1 BCL2 PARP1
GO:0031960	response to corticosteroid	4/20	166/18493	0.001	TGFB1 BCL2 PARP1 IL6
GO:0001541	ovarian follicle development	3/20	56/18493	0.001	PHB BCL2L1 BCL2
GO:0001654	eye development	5/20	351/18493	0.001	JUN STAT3 TGFB1 SMAD3 BCL2
GO:0045862	positive regulation of proteolysis	5/20	352/18493	0.001	PHB STAT3 SMAD3 GSK3A GSK3B
GO:0150063	visual system development	5/20	352/18493	0.001	JUN STAT3 TGFB1 SMAD3 BCL2
GO:1902110	positive regulation of mitochondrial membrane permeability involved in apoptotic process	3/20	57/18493	0.001	BCL2 GSK3A GSK3B
GO:2001244	positive regulation of intrinsic apoptotic signaling pathway	3/20	57/18493	0.001	BCL2L1 MCL1 BCL2
GO:0048880	sensory system development	5/20	357/18493	0.001	JUN STAT3 TGFB1 SMAD3 BCL2
GO:0001836	release of cytochrome c from mitochondria	3/20	58/18493	0.001	JUN BCL2L1 BCL2
GO:1902686	mitochondrial outer membrane permeabilization involved in programmed cell death	3/20	59/18493	0.001	BCL2 GSK3A GSK3B
GO:0010821	regulation of mitochondrion organization	4/20	176/18493	0.001	BCL2L1 BCL2 GSK3A GSK3B
GO:0051170	import into nucleus	4/20	178/18493	0.001	KPNB1 STAT3 TGFB1 SMAD3
GO:0050678	regulation of epithelial cell proliferation	5/20	368/18493	0.001	STAT5A JUN STAT3 TGFB1 SMAD3
GO:0035794	positive regulation of mitochondrial membrane permeability	3/20	61/18493	0.001	BCL2 GSK3A GSK3B
GO:1902108	regulation of mitochondrial membrane permeability involved in apoptotic process	3/20	63/18493	0.001	BCL2 GSK3A GSK3B
GO:1905710	positive regulation of membrane permeability	3/20	63/18493	0.001	BCL2 GSK3A GSK3B
GO:2000377	regulation of reactive oxygen species metabolic process	4/20	185/18493	0.001	STAT3 TGFB1 SMAD3 BCL2
GO:0071478	cellular response to radiation	4/20	186/18493	0.001	TGFB1 BCL2L1 NPM1 PARP1
GO:0001503	ossification	5/20	380/18493	0.001	PHB TGFB1 SMAD3 BCL2 IL6
GO:0031099	regeneration	4/20	188/18493	0.001	PHB JUN TGFB1 BCL2

ID	Description	GeneRatio	BgRatio	p.adjust	geneID
GO:0007179	transforming growth factor beta receptor signaling pathway	4/20	193/18493	0.001	JUN TGFB1 SMAD3 PARP1
GO:0007623	circadian rhythm	4/20	193/18493	0.001	JUN PPP1CA ATF5 GSK3B
GO:0030278	regulation of ossification	4/20	193/18493	0.001	TGFB1 SMAD3 BCL2 IL6
GO:0046824	positive regulation of nucleocytoplasmic transport	3/20	67/18493	0.001	TGFB1 SMAD3 GSK3B
GO:0033148	positive regulation of intracellular estrogen receptor signaling pathway	2/20	10/18493	0.001	SKP2 PARP1
GO:0071104	response to interleukin-9	2/20	10/18493	0.001	STAT5A STAT3
GO:0033692	cellular polysaccharide biosynthetic process	3/20	68/18493	0.001	PPP1CA GSK3A GSK3B
GO:0051881	regulation of mitochondrial membrane potential	3/20	68/18493	0.001	BCL2L1 BCL2 PARP1
GO:0040014	regulation of multicellular organism growth	3/20	69/18493	0.001	STAT5A STAT3 BCL2
GO:0050679	positive regulation of epithelial cell proliferation	4/20	203/18493	0.001	STAT5A JUN STAT3 TGFB1
GO:1901201	regulation of extracellular matrix assembly	2/20	11/18493	0.001	TGFB1 SMAD3
GO:2001020	regulation of response to DNA damage stimulus	4/20	206/18493	0.001	BCL2L1 MCL1 BCL2 PARP1
GO:0051090	regulation of DNA-binding transcription factor activity	5/20	412/18493	0.001	JUN TGFB1 SMAD3 NPM1 IL6
GO:0005977	glycogen metabolic process	3/20	73/18493	0.001	PPP1CA GSK3A GSK3B
GO:0016051	carbohydrate biosynthetic process	4/20	209/18493	0.001	PPP1CA TGFB1 GSK3A GSK3B
GO:0051701	interaction with host	4/20	209/18493	0.001	KPNB1 TGFB1 SMAD3 BCL2L1
GO:0006073	cellular glucan metabolic process	3/20	74/18493	0.001	PPP1CA GSK3A GSK3B
GO:0010611	regulation of cardiac muscle hypertrophy	3/20	74/18493	0.001	SMAD3 GSK3A PARP1
GO:0044042	glucan metabolic process	3/20	74/18493	0.001	PPP1CA GSK3A GSK3B
GO:0048762	mesenchymal cell differentiation	4/20	210/18493	0.001	TGFB1 SMAD3 BCL2 GSK3B
GO:0050673	epithelial cell proliferation	5/20	420/18493	0.001	STAT5A JUN STAT3 TGFB1 SMAD3
GO:0014743	regulation of muscle hypertrophy	3/20	76/18493	0.001	SMAD3 GSK3A PARP1
GO:0060391	positive regulation of SMAD protein signal transduction	2/20	12/18493	0.001	TGFB1 PARP1
GO:0072540	T-helper 17 cell lineage commitment	2/20	12/18493	0.001	STAT3 IL6

ID	Description	GeneRatio	BgRatio	p.adjust	geneID
GO:0017038	protein import	4/20	214/18493	0.001	KPNB1 STAT3 TGFB1 SMAD3
GO:0032388	positive regulation of intracellular transport	4/20	214/18493	0.001	TGFB1 SMAD3 GSK3A GSK3B
GO:0031016	pancreas development	3/20	77/18493	0.001	GSK3A GSK3B IL6
GO:0045444	fat cell differentiation	4/20	217/18493	0.001	TGFB1 SMAD3 ATF5 IL6
GO:1900182	positive regulation of protein localization to nucleus	3/20	78/18493	0.001	TGFB1 SMAD3 PARP1
GO:0048732	gland development	5/20	434/18493	0.001	STAT5A JUN TGFB1 SMAD3 BCL2
GO:0030279	negative regulation of ossification	3/20	79/18493	0.001	TGFB1 SMAD3 BCL2
GO:0033143	regulation of intracellular steroid hormone receptor signaling pathway	3/20	79/18493	0.001	PHB SKP2 PARP1
GO:0035723	interleukin-15-mediated signaling pathway	2/20	13/18493	0.001	STAT5A STAT3
GO:0060397	JAK-STAT cascade involved in growth hormone signaling pathway	2/20	13/18493	0.001	STAT5A STAT3
GO:0071350	cellular response to interleukin-15	2/20	13/18493	0.001	STAT5A STAT3
GO:0006979	response to oxidative stress	5/20	441/18493	0.001	JUN MCL1 BCL2 PARP1 IL6
GO:0051607	defense response to virus	4/20	228/18493	0.001	IRF3 BCL2 SKP2 IL6
GO:0042326	negative regulation of phosphorylation	5/20	448/18493	0.001	PHB JUN STAT3 TGFB1 NPM1
GO:0034637	cellular carbohydrate biosynthetic process	3/20	83/18493	0.001	PPP1CA GSK3A GSK3B
GO:0071216	cellular response to biotic stimulus	4/20	229/18493	0.001	TGFB1 IRF3 GSK3B IL6
GO:0042110	T cell activation	5/20	451/18493	0.001	STAT3 TGFB1 SMAD3 BCL2 IL6
GO:0045778	positive regulation of ossification	3/20	84/18493	0.001	TGFB1 SMAD3 IL6
GO:0034248	regulation of cellular amide metabolic process	5/20	453/18493	0.001	STAT3 PPP1CA GSK3A NPM1 IL6
GO:0033157	regulation of intracellular protein transport	4/20	232/18493	0.001	TGFB1 SMAD3 GSK3A GSK3B
GO:0043558	regulation of translational initiation in response to stress	2/20	14/18493	0.001	PPP1CA NPM1
GO:0070672	response to interleukin-15	2/20	14/18493	0.001	STAT5A STAT3
GO:0030217	T cell differentiation	4/20	234/18493	0.001	STAT3 TGFB1 BCL2 IL6
GO:0033002	muscle cell proliferation	4/20	236/18493	0.001	JUN STAT3 SKP2 IL6

ID	Description	GeneRatio	BgRatio	p.adjust	geneID
GO:0060828	regulation of canonical Wnt signaling pathway	4/20	236/18493	0.001	PPP1CA SMAD3 GSK3A GSK3B
GO:0006112	energy reserve metabolic process	3/20	87/18493	0.001	PPP1CA GSK3A GSK3B
GO:0001776	leukocyte homeostasis	3/20	88/18493	0.001	TGFB1 BCL2 IL6
GO:0033689	negative regulation of osteoblast proliferation	2/20	15/18493	0.001	SMAD3 BCL2
GO:2001170	negative regulation of ATP biosynthetic process	2/20	15/18493	0.001	STAT3 PARP1
GO:0071560	cellular response to transforming growth factor beta stimulus	4/20	242/18493	0.001	JUN TGFB1 SMAD3 PARP1
GO:0051899	membrane depolarization	3/20	90/18493	0.001	JUN BCL2 PARP1
GO:0031330	negative regulation of cellular catabolic process	4/20	245/18493	0.001	STAT3 MCL1 BCL2 GSK3A
GO:0016311	dephosphorylation	5/20	475/18493	0.001	PPP1CA TGFB1 SMAD3 BCL2 GSK3B
GO:0045639	positive regulation of myeloid cell differentiation	3/20	91/18493	0.001	JUN STAT3 TGFB1
GO:0048872	homeostasis of number of cells	4/20	248/18493	0.001	STAT3 TGFB1 BCL2 IL6
GO:0071559	response to transforming growth factor beta	4/20	249/18493	0.001	JUN TGFB1 SMAD3 PARP1
GO:0036473	cell death in response to oxidative stress	3/20	93/18493	0.001	MCL1 BCL2 PARP1
GO:0002295	T-helper cell lineage commitment	2/20	16/18493	0.001	STAT3 IL6
GO:0003007	heart morphogenesis	4/20	253/18493	0.002	JUN TGFB1 SMAD3 GSK3A
GO:0051091	positive regulation of DNA-binding transcription factor activity	4/20	255/18493	0.002	TGFB1 SMAD3 NPM1 IL6
GO:0001657	ureteric bud development	3/20	96/18493	0.002	TGFB1 SMAD3 BCL2
GO:2001243	negative regulation of intrinsic apoptotic signaling pathway	3/20	96/18493	0.002	BCL2L1 MCL1 BCL2
GO:0045165	cell fate commitment	4/20	257/18493	0.002	STAT3 MCL1 BCL2 IL6
GO:0072163	mesonephric epithelium development	3/20	97/18493	0.002	TGFB1 SMAD3 BCL2
GO:0072164	mesonephric tubule development	3/20	97/18493	0.002	TGFB1 SMAD3 BCL2
GO:0030809	negative regulation of nucleotide biosynthetic process	2/20	17/18493	0.002	STAT3 PARP1
GO:0033145	positive regulation of intracellular steroid hormone receptor signaling pathway	2/20	17/18493	0.002	SKP2 PARP1
GO:0043373	CD4-positive, alpha-beta T cell lineage commitment	2/20	17/18493	0.002	STAT3 IL6
GO:0070102	interleukin-6-mediated signaling pathway	2/20	17/18493	0.002	STAT3 IL6
GO:1900372	negative regulation of purine nucleotide biosynthetic process	2/20	17/18493	0.002	STAT3 PARP1

ID	Description	GeneRatio	BgRatio	p.adjust	geneID
GO:2000811	negative regulation of anoikis	2/20	17/18493	0.002	MCL1 BCL2
GO:0031334	positive regulation of protein complex assembly	4/20	259/18493	0.002	JUN TGFB1 GSK3B PARP1
GO:0071383	cellular response to steroid hormone stimulus	4/20	259/18493	0.002	PHB TGFB1 SKP2 PARP1
GO:0008585	female gonad development	3/20	98/18493	0.002	PHB BCL2L1 BCL2
GO:0048661	positive regulation of smooth muscle cell proliferation	3/20	98/18493	0.002	JUN SKP2 IL6
GO:0044264	cellular polysaccharide metabolic process	3/20	99/18493	0.002	PPP1CA GSK3A GSK3B
GO:0034976	response to endoplasmic reticulum stress	4/20	264/18493	0.002	JUN BCL2 GSK3A GSK3B
GO:0043555	regulation of translation in response to stress	2/20	18/18493	0.002	PPP1CA NPM1
GO:0001823	mesonephros development	3/20	101/18493	0.002	TGFB1 SMAD3 BCL2
GO:0046632	alpha-beta T cell differentiation	3/20	101/18493	0.002	STAT3 BCL2 IL6
GO:0008630	intrinsic apoptotic signaling pathway in response to DNA damage	3/20	102/18493	0.002	BCL2L1 MCL1 BCL2
GO:0060485	mesenchyme development	4/20	269/18493	0.002	TGFB1 SMAD3 BCL2 GSK3B
GO:0033138	positive regulation of peptidyl-serine phosphorylation	3/20	103/18493	0.002	TGFB1 BCL2 IL6
GO:0046545	development of primary female sexual characteristics	3/20	103/18493	0.002	PHB BCL2L1 BCL2
GO:2001237	negative regulation of extrinsic apoptotic signaling pathway	3/20	104/18493	0.002	BCL2L1 MCL1 BCL2
GO:0010523	negative regulation of calcium ion transport into cytosol	2/20	19/18493	0.002	TGFB1 BCL2
GO:0043502	regulation of muscle adaptation	3/20	106/18493	0.002	SMAD3 GSK3A PARP1
GO:0048511	rhythmic process	4/20	276/18493	0.002	JUN PPP1CA ATF5 GSK3B
GO:0072593	reactive oxygen species metabolic process	4/20	276/18493	0.002	STAT3 TGFB1 SMAD3 BCL2
GO:0003300	cardiac muscle hypertrophy	3/20	107/18493	0.002	SMAD3 GSK3A PARP1
GO:0044262	cellular carbohydrate metabolic process	4/20	278/18493	0.002	STAT3 PPP1CA GSK3A GSK3B
GO:0050768	negative regulation of neurogenesis	4/20	279/18493	0.002	STAT3 TGFB1 GSK3B IL6
GO:0045930	negative regulation of mitotic cell cycle	4/20	281/18493	0.002	TGFB1 SMAD3 BCL2L1 BCL2
GO:0003323	type B pancreatic cell development	2/20	20/18493	0.002	GSK3A GSK3B
GO:0023019	signal transduction involved in regulation of gene expression	2/20	20/18493	0.002	SMAD3 PARP1

ID	Description	GeneRatio	BgRatio	p.adjust	geneID
GO:1903055	positive regulation of extracellular matrix organization	2/20	20/18493	0.002	TGFB1 SMAD3
GO:0014897	striated muscle hypertrophy	3/20	109/18493	0.002	SMAD3 GSK3A PARP1
GO:0018105	peptidyl-serine phosphorylation	4/20	282/18493	0.002	TGFB1 BCL2 GSK3B IL6
GO:0060070	canonical Wnt signaling pathway	4/20	284/18493	0.002	PPP1CA SMAD3 GSK3A GSK3B
GO:0010906	regulation of glucose metabolic process	3/20	111/18493	0.002	PPP1CA GSK3A GSK3B
GO:0014896	muscle hypertrophy	3/20	111/18493	0.002	SMAD3 GSK3A PARP1
GO:1903828	negative regulation of cellular protein localization	3/20	111/18493	0.002	TGFB1 BCL2L1 GSK3B
GO:0097193	intrinsic apoptotic signaling pathway	4/20	287/18493	0.002	BCL2L1 MCL1 BCL2 PARP1
GO:0001938	positive regulation of endothelial cell proliferation	3/20	112/18493	0.002	STAT5A JUN STAT3
GO:0010822	positive regulation of mitochondrion organization	3/20	112/18493	0.002	BCL2 GSK3A GSK3B
GO:0046822	regulation of nucleocytoplasmic transport	3/20	112/18493	0.002	TGFB1 SMAD3 GSK3B
GO:0051900	regulation of mitochondrial depolarization	2/20	21/18493	0.002	BCL2 PARP1
GO:0060390	regulation of SMAD protein signal transduction	2/20	21/18493	0.002	TGFB1 PARP1
GO:0071359	cellular response to dsRNA	2/20	21/18493	0.002	IRF3 NPM1
GO:1904886	beta-catenin destruction complex disassembly	2/20	21/18493	0.002	PPP1CA GSK3B
GO:0051153	regulation of striated muscle cell differentiation	3/20	113/18493	0.002	TGFB1 BCL2 GSK3A
GO:0006925	inflammatory cell apoptotic process	2/20	22/18493	0.002	IRF3 IL6
GO:1903077	negative regulation of protein localization to plasma membrane	2/20	22/18493	0.002	TGFB1 BCL2L1
GO:2000637	positive regulation of gene silencing by miRNA	2/20	22/18493	0.002	STAT3 TGFB1
GO:0006754	ATP biosynthetic process	3/20	116/18493	0.002	STAT3 TGFB1 PARP1
GO:0001952	regulation of cell-matrix adhesion	3/20	117/18493	0.002	SMAD3 BCL2 GSK3B
GO:0051961	negative regulation of nervous system development	4/20	298/18493	0.002	STAT3 TGFB1 GSK3B IL6
GO:0046660	female sex differentiation	3/20	118/18493	0.002	PHB BCL2L1 BCL2
GO:0051882	mitochondrial depolarization	2/20	23/18493	0.002	BCL2 PARP1
GO:0060148	positive regulation of posttranscriptional gene silencing	2/20	23/18493	0.002	STAT3 TGFB1
GO:0060396	growth hormone receptor signaling pathway	2/20	23/18493	0.002	STAT5A STAT3
GO:1903579	negative regulation of ATP metabolic process	2/20	23/18493	0.002	STAT3 PARP1

ID	Description	GeneRatio	BgRatio	p.adjust	geneID
GO:0010506	regulation of autophagy	4/20	303/18493	0.002	TFEB MCL1 BCL2 GSK3A
GO:0043500	muscle adaptation	3/20	120/18493	0.002	SMAD3 GSK3A PARP1
GO:0051101	regulation of DNA binding	3/20	122/18493	0.002	JUN TGFB1 PARP1
GO:0009416	response to light stimulus	4/20	308/18493	0.002	PPP1CA BCL2 NPM1 PARP1
GO:0071378	cellular response to growth hormone stimulus	2/20	24/18493	0.002	STAT5A STAT3
GO:1903203	regulation of oxidative stress-induced neuron death	2/20	24/18493	0.002	MCL1 PARP1
GO:1904376	negative regulation of protein localization to cell periphery	2/20	24/18493	0.002	TGFB1 BCL2L1
GO:2000209	regulation of anoikis	2/20	24/18493	0.002	MCL1 BCL2
GO:2000679	positive regulation of transcription regulatory region DNA binding	2/20	24/18493	0.002	TGFB1 PARP1
GO:0030111	regulation of Wnt signaling pathway	4/20	311/18493	0.002	PPP1CA SMAD3 GSK3A GSK3B
GO:0060562	epithelial tube morphogenesis	4/20	313/18493	0.003	PPP1CA TGFB1 SMAD3 BCL2
GO:0002068	glandular epithelial cell development	2/20	25/18493	0.003	GSK3A GSK3B
GO:0003309	type B pancreatic cell differentiation	2/20	25/18493	0.003	GSK3A GSK3B
GO:0019054	modulation by virus of host process	2/20	25/18493	0.003	KPNB1 BCL2L1
GO:0036475	neuron death in response to oxidative stress	2/20	25/18493	0.003	MCL1 PARP1
GO:0085029	extracellular matrix assembly	2/20	25/18493	0.003	TGFB1 SMAD3
GO:1900543	negative regulation of purine nucleotide metabolic process	2/20	25/18493	0.003	STAT3 PARP1
GO:0009206	purine ribonucleoside triphosphate biosynthetic process	3/20	127/18493	0.003	STAT3 TGFB1 PARP1
GO:0006470	protein dephosphorylation	4/20	318/18493	0.003	PPP1CA TGFB1 BCL2 GSK3B
GO:0071214	cellular response to abiotic stimulus	4/20	319/18493	0.003	TGFB1 BCL2L1 NPM1 PARP1
GO:0104004	cellular response to environmental stimulus	4/20	319/18493	0.003	TGFB1 BCL2L1 NPM1 PARP1
GO:0009145	purine nucleoside triphosphate biosynthetic process	3/20	128/18493	0.003	STAT3 TGFB1 PARP1
GO:0045598	regulation of fat cell differentiation	3/20	128/18493	0.003	TGFB1 SMAD3 IL6
GO:0050921	positive regulation of chemotaxis	3/20	130/18493	0.003	TGFB1 SMAD3 IL6
GO:0045980	negative regulation of nucleotide metabolic process	2/20	26/18493	0.003	STAT3 PARP1
GO:0072539	T-helper 17 cell differentiation	2/20	26/18493	0.003	STAT3 IL6

ID	Description	GeneRatio	BgRatio	p.adjust	geneID
GO:0032496	response to lipopolysaccharide	4/20	324/18493	0.003	JUN TGFB1 IRF3 IL6
GO:0048565	digestive tract development	3/20	131/18493	0.003	TGFB1 SMAD3 BCL2
GO:0071496	cellular response to external stimulus	4/20	328/18493	0.003	JUN TGFB1 BCL2 SKP2
GO:0009201	ribonucleoside triphosphate biosynthetic process	3/20	133/18493	0.003	STAT3 TGFB1 PARP1
GO:0035883	enteroendocrine cell differentiation	2/20	27/18493	0.003	GSK3A GSK3B
GO:0035902	response to immobilization stress	2/20	27/18493	0.003	PHB TGFB1
GO:0046631	alpha-beta T cell activation	3/20	134/18493	0.003	STAT3 BCL2 IL6
GO:0001837	epithelial to mesenchymal transition	3/20	135/18493	0.003	TGFB1 SMAD3 GSK3B
GO:0033135	regulation of peptidyl-serine phosphorylation	3/20	135/18493	0.003	TGFB1 BCL2 IL6
GO:0009411	response to UV	3/20	136/18493	0.003	BCL2 NPM1 PARP1
GO:0033688	regulation of osteoblast proliferation	2/20	28/18493	0.003	SMAD3 BCL2
GO:0072538	T-helper 17 type immune response	2/20	28/18493	0.003	STAT3 IL6
GO:1904646	cellular response to amyloid-beta	2/20	28/18493	0.003	GSK3B PARP1
GO:0001889	liver development	3/20	137/18493	0.003	JUN TGFB1 SMAD3
GO:0002237	response to molecule of bacterial origin	4/20	337/18493	0.003	JUN TGFB1 IRF3 IL6
GO:0007178	transmembrane receptor protein serine/threonine kinase signaling pathway	4/20	340/18493	0.003	JUN TGFB1 SMAD3 PARP1
GO:0034250	positive regulation of cellular amide metabolic process	3/20	140/18493	0.003	GSK3A NPM1 IL6
GO:0061008	hepaticobiliary system development	3/20	140/18493	0.003	JUN TGFB1 SMAD3
GO:0072073	kidney epithelium development	3/20	140/18493	0.003	TGFB1 SMAD3 BCL2
GO:0044068	modulation by symbiont of host cellular process	2/20	29/18493	0.003	KPNB1 BCL2L1
GO:0070306	lens fiber cell differentiation	2/20	29/18493	0.003	TGFB1 SMAD3
GO:1902175	regulation of oxidative stress-induced intrinsic apoptotic signaling pathway	2/20	29/18493	0.003	MCL1 PARP1
GO:0009127	purine nucleoside monophosphate biosynthetic process	3/20	141/18493	0.003	STAT3 TGFB1 PARP1
GO:0009168	purine ribonucleoside monophosphate biosynthetic process	3/20	141/18493	0.003	STAT3 TGFB1 PARP1
GO:0042542	response to hydrogen peroxide	3/20	141/18493	0.003	JUN BCL2 IL6
GO:0030098	lymphocyte differentiation	4/20	344/18493	0.003	STAT3 TGFB1 BCL2 IL6
GO:0030518	intracellular steroid hormone receptor signaling pathway	3/20	142/18493	0.003	PHB SKP2 PARP1
GO:0055123	digestive system development	3/20	143/18493	0.003	TGFB1 SMAD3 BCL2

ID	Description	GeneRatio	BgRatio	p.adjust	geneID
GO:0038111	interleukin-7-mediated signaling pathway	2/20	30/18493	0.003	STAT5A STAT3
GO:1905476	negative regulation of protein localization to membrane	2/20	30/18493	0.003	TGFB1 BCL2L1
GO:0006959	humoral immune response	4/20	349/18493	0.003	TFEB PHB BCL2 IL6
GO:0050871	positive regulation of B cell activation	3/20	144/18493	0.003	TGFB1 BCL2 IL6
GO:0009142	nucleoside triphosphate biosynthetic process	3/20	145/18493	0.003	STAT3 TGFB1 PARP1
GO:0033687	osteoblast proliferation	2/20	31/18493	0.003	SMAD3 BCL2
GO:0045745	positive regulation of G protein-coupled receptor signaling pathway	2/20	31/18493	0.003	PHB GSK3A
GO:0098760	response to interleukin-7	2/20	31/18493	0.003	STAT5A STAT3
GO:0098761	cellular response to interleukin-7	2/20	31/18493	0.003	STAT5A STAT3
GO:1902230	negative regulation of intrinsic apoptotic signaling pathway in response to DNA damage	2/20	31/18493	0.003	BCL2L1 BCL2
GO:1904645	response to amyloid-beta	2/20	31/18493	0.003	GSK3B PARP1
GO:0007259	JAK-STAT cascade	3/20	147/18493	0.003	STAT5A STAT3 IL6
GO:0043524	negative regulation of neuron apoptotic process	3/20	147/18493	0.003	JUN BCL2L1 BCL2
GO:0010038	response to metal ion	4/20	358/18493	0.003	JUN PPP1CA BCL2 PARP1
GO:0051384	response to glucocorticoid	3/20	149/18493	0.003	TGFB1 BCL2 IL6
GO:0048754	branching morphogenesis of an epithelial tube	3/20	150/18493	0.004	PPP1CA TGFB1 BCL2
GO:0010574	regulation of vascular endothelial growth factor production	2/20	32/18493	0.004	TGFB1 IL6
GO:0097696	STAT cascade	3/20	151/18493	0.004	STAT5A STAT3 IL6
GO:0016052	carbohydrate catabolic process	3/20	152/18493	0.004	STAT3 PPP1CA GSK3A
GO:0009156	ribonucleoside monophosphate biosynthetic process	3/20	155/18493	0.004	STAT3 TGFB1 PARP1
GO:0010573	vascular endothelial growth factor production	2/20	34/18493	0.004	TGFB1 IL6
GO:0043276	anoikis	2/20	34/18493	0.004	MCL1 BCL2
GO:0042176	regulation of protein catabolic process	4/20	375/18493	0.004	PHB SMAD3 GSK3A GSK3B
GO:0010614	negative regulation of cardiac muscle hypertrophy	2/20	35/18493	0.004	SMAD3 GSK3A
GO:0030501	positive regulation of bone mineralization	2/20	35/18493	0.004	TGFB1 SMAD3
GO:0014706	striated muscle tissue development	4/20	379/18493	0.004	TGFB1 SMAD3 BCL2 GSK3A
GO:0060416	response to growth hormone	2/20	36/18493	0.004	STAT5A STAT3

ID	Description	GeneRatio	BgRatio	p.adjust	geneID
GO:0018108	peptidyl-tyrosine phosphorylation	4/20	387/18493	0.004	STAT5A STAT3 TGFB1 IL6
GO:0010634	positive regulation of epithelial cell migration	3/20	165/18493	0.004	STAT5A JUN TGFB1
GO:0051100	negative regulation of binding	3/20	165/18493	0.004	JUN PPP1CA GSK3B
GO:0018212	peptidyl-tyrosine modification	4/20	390/18493	0.004	STAT5A STAT3 TGFB1 IL6
GO:0014741	negative regulation of muscle hypertrophy	2/20	37/18493	0.004	SMAD3 GSK3A
GO:0009124	nucleoside monophosphate biosynthetic process	3/20	167/18493	0.005	STAT3 TGFB1 PARP1
GO:0048660	regulation of smooth muscle cell proliferation	3/20	168/18493	0.005	JUN SKP2 IL6
GO:0060537	muscle tissue development	4/20	395/18493	0.005	TGFB1 SMAD3 BCL2 GSK3A
GO:0000060	protein import into nucleus, translocation	2/20	38/18493	0.005	KPNB1 TGFB1
GO:0045785	positive regulation of cell adhesion	4/20	397/18493	0.005	TGFB1 SMAD3 GSK3B IL6
GO:0048659	smooth muscle cell proliferation	3/20	170/18493	0.005	JUN SKP2 IL6
GO:0033146	regulation of intracellular estrogen receptor signaling pathway	2/20	39/18493	0.005	SKP2 PARP1
GO:0043029	T cell homeostasis	2/20	39/18493	0.005	TGFB1 BCL2
GO:1902229	regulation of intrinsic apoptotic signaling pathway in response to DNA damage	2/20	39/18493	0.005	BCL2L1 BCL2
GO:1903053	regulation of extracellular matrix organization	2/20	39/18493	0.005	TGFB1 SMAD3
GO:0006417	regulation of translation	4/20	402/18493	0.005	STAT3 PPP1CA NPM1 IL6
GO:0007517	muscle organ development	4/20	403/18493	0.005	TGFB1 SMAD3 BCL2 GSK3A
GO:0002285	lymphocyte activation involved in immune response	3/20	174/18493	0.005	STAT3 TGFB1 IL6
GO:0030099	myeloid cell differentiation	4/20	405/18493	0.005	JUN STAT3 TGFB1 PARP1
GO:0001936	regulation of endothelial cell proliferation	3/20	175/18493	0.005	STAT5A JUN STAT3
GO:0070507	regulation of microtubule cytoskeleton organization	3/20	175/18493	0.005	ATF5 GSK3B NPM1
GO:0030225	macrophage differentiation	2/20	40/18493	0.005	TGFB1 PARP1
GO:0044275	cellular carbohydrate catabolic process	2/20	40/18493	0.005	PPP1CA GSK3A
GO:0001933	negative regulation of protein phosphorylation	4/20	408/18493	0.005	PHB JUN TGFB1 NPM1
GO:1905475	regulation of protein localization to membrane	3/20	177/18493	0.005	TGFB1 BCL2L1 BCL2
GO:0051052	regulation of DNA metabolic process	4/20	412/18493	0.005	JUN TGFB1 NPM1 PARP1

ID	Description	GeneRatio	BgRatio	p.adjust	geneID
GO:1901987	regulation of cell cycle phase transition	4/20	412/18493	0.005	TGFB1 ATF5 BCL2 NPM1
GO:0006611	protein export from nucleus	3/20	180/18493	0.005	TGFB1 GSK3B NPM1
GO:0051099	positive regulation of binding	3/20	181/18493	0.005	TGFB1 GSK3B PARP1
GO:0042307	positive regulation of protein import into nucleus	2/20	42/18493	0.005	TGFB1 SMAD3
GO:0051147	regulation of muscle cell differentiation	3/20	182/18493	0.005	TGFB1 BCL2 GSK3A
GO:0061138	morphogenesis of a branching epithelium	3/20	182/18493	0.005	PPP1CA TGFB1 BCL2
GO:0050864	regulation of B cell activation	3/20	183/18493	0.005	TGFB1 BCL2 IL6
GO:1903708	positive regulation of hemopoiesis	3/20	183/18493	0.005	JUN STAT3 TGFB1
GO:0001819	positive regulation of cytokine production	4/20	422/18493	0.005	TGFB1 SMAD3 IRF3 IL6
GO:0032386	regulation of intracellular transport	4/20	422/18493	0.005	TGFB1 SMAD3 GSK3A GSK3B
GO:0007212	dopamine receptor signaling pathway	2/20	43/18493	0.006	GSK3A GSK3B
GO:0032965	regulation of collagen biosynthetic process	2/20	43/18493	0.006	TGFB1 IL6
GO:0050731	positive regulation of peptidyl-tyrosine phosphorylation	3/20	186/18493	0.006	STAT3 TGFB1 IL6
GO:0048608	reproductive structure development	4/20	428/18493	0.006	TFEB PHB BCL2L1 BCL2
GO:0002639	positive regulation of immunoglobulin production	2/20	44/18493	0.006	TGFB1 IL6
GO:0070169	positive regulation of biomineral tissue development	2/20	44/18493	0.006	TGFB1 SMAD3
GO:1904591	positive regulation of protein import	2/20	44/18493	0.006	TGFB1 SMAD3
GO:0061458	reproductive system development	4/20	431/18493	0.006	TFEB PHB BCL2L1 BCL2
GO:0001935	endothelial cell proliferation	3/20	190/18493	0.006	STAT5A JUN STAT3
GO:1901654	response to ketone	3/20	190/18493	0.006	TGFB1 BCL2L1 PARP1
GO:0043401	steroid hormone mediated signaling pathway	3/20	191/18493	0.006	PHB SKP2 PARP1
GO:0030857	negative regulation of epithelial cell differentiation	2/20	46/18493	0.006	GSK3A GSK3B
GO:0031103	axon regeneration	2/20	46/18493	0.006	JUN BCL2
GO:0035196	production of miRNAs involved in gene silencing by miRNA	2/20	46/18493	0.006	TGFB1 SMAD3
GO:0051168	nuclear export	3/20	194/18493	0.006	TGFB1 GSK3B NPM1
GO:0002067	glandular epithelial cell differentiation	2/20	47/18493	0.006	GSK3A GSK3B
GO:0006953	acute-phase response	2/20	47/18493	0.006	STAT3 IL6
GO:0150076	neuroinflammatory response	2/20	47/18493	0.006	JUN IL6

ID	Description	GeneRatio	BgRatio	p.adjust	geneID
GO:0001763	morphogenesis of a branching structure	3/20	196/18493	0.006	PPP1CA TGFB1 BCL2
GO:0002573	myeloid leukocyte differentiation	3/20	197/18493	0.006	JUN TGFB1 PARP1
GO:0003254	regulation of membrane depolarization	2/20	48/18493	0.006	BCL2 PARP1
GO:0010712	regulation of collagen metabolic process	2/20	48/18493	0.006	TGFB1 IL6
GO:0043525	positive regulation of neuron apoptotic process	2/20	48/18493	0.006	JUN MCL1
GO:0098586	cellular response to virus	2/20	48/18493	0.006	IRF3 BCL2L1
GO:2000725	regulation of cardiac muscle cell differentiation	2/20	48/18493	0.006	TGFB1 GSK3A
GO:0071222	cellular response to lipopolysaccharide	3/20	199/18493	0.006	TGFB1 IRF3 IL6
GO:0043254	regulation of protein complex assembly	4/20	451/18493	0.007	JUN TGFB1 GSK3B PARP1
GO:0010718	positive regulation of epithelial to mesenchymal transition	2/20	49/18493	0.007	TGFB1 SMAD3
GO:0031050	dsRNA processing	2/20	49/18493	0.007	TGFB1 SMAD3
GO:0048546	digestive tract morphogenesis	2/20	49/18493	0.007	SMAD3 BCL2
GO:0070918	production of small RNA involved in gene silencing by RNA	2/20	49/18493	0.007	TGFB1 SMAD3
GO:0006006	glucose metabolic process	3/20	204/18493	0.007	PPP1CA GSK3A GSK3B
GO:0032886	regulation of microtubule-based process	3/20	204/18493	0.007	ATF5 GSK3B NPM1
GO:0001954	positive regulation of cell-matrix adhesion	2/20	50/18493	0.007	SMAD3 GSK3B
GO:0050670	regulation of lymphocyte proliferation	3/20	205/18493	0.007	TGFB1 BCL2 IL6
GO:0032944	regulation of mononuclear cell proliferation	3/20	206/18493	0.007	TGFB1 BCL2 IL6
GO:0035303	regulation of dephosphorylation	3/20	206/18493	0.007	TGFB1 SMAD3 GSK3B
GO:0071219	cellular response to molecule of bacterial origin	3/20	206/18493	0.007	TGFB1 IRF3 IL6
GO:0016055	Wnt signaling pathway	4/20	463/18493	0.007	PPP1CA SMAD3 GSK3A GSK3B
GO:2000677	regulation of transcription regulatory region DNA binding	2/20	51/18493	0.007	TGFB1 PARP1
GO:0198738	cell-cell signaling by wnt	4/20	465/18493	0.007	PPP1CA SMAD3 GSK3A GSK3B
GO:0006914	autophagy	4/20	466/18493	0.007	TFEB MCL1 BCL2 GSK3A
GO:0061919	process utilizing autophagic mechanism	4/20	466/18493	0.007	TFEB MCL1 BCL2 GSK3A
GO:0002699	positive regulation of immune effector process	3/20	209/18493	0.007	PHB TGFB1 IL6
GO:0010810	regulation of cell-substrate adhesion	3/20	209/18493	0.007	SMAD3 BCL2 GSK3B

ID	Description	GeneRatio	BgRatio	p.adjust	geneID
GO:0002763	positive regulation of myeloid leukocyte differentiation	2/20	52/18493	0.007	JUN TGFB1
GO:0032964	collagen biosynthetic process	2/20	52/18493	0.007	TGFB1 IL6
GO:0048641	regulation of skeletal muscle tissue development	2/20	52/18493	0.007	TGFB1 BCL2
GO:2001169	regulation of ATP biosynthetic process	2/20	52/18493	0.007	STAT3 PARP1
GO:0050920	regulation of chemotaxis	3/20	210/18493	0.007	TGFB1 SMAD3 IL6
GO:0002526	acute inflammatory response	3/20	211/18493	0.007	PHB STAT3 IL6
GO:0006091	generation of precursor metabolites and energy	4/20	471/18493	0.007	STAT3 PPP1CA GSK3A GSK3B
GO:0043331	response to dsRNA	2/20	53/18493	0.007	IRF3 NPM1
GO:0001649	osteoblast differentiation	3/20	214/18493	0.007	PHB SMAD3 IL6
GO:0008406	gonad development	3/20	216/18493	0.008	PHB BCL2L1 BCL2
GO:0070663	regulation of leukocyte proliferation	3/20	216/18493	0.008	TGFB1 BCL2 IL6
GO:0002009	morphogenesis of an epithelium	4/20	479/18493	0.008	PPP1CA TGFB1 SMAD3 BCL2
GO:0043393	regulation of protein binding	3/20	217/18493	0.008	PPP1CA BCL2 GSK3B
GO:0044839	cell cycle G2/M phase transition	3/20	218/18493	0.008	ATF5 SKP2 NPM1
GO:0031102	neuron projection regeneration	2/20	55/18493	0.008	JUN BCL2
GO:0048146	positive regulation of fibroblast proliferation	2/20	55/18493	0.008	JUN TGFB1
GO:0051054	positive regulation of DNA metabolic process	3/20	220/18493	0.008	JUN TGFB1 PARP1
GO:0031663	lipopolysaccharide-mediated signaling pathway	2/20	56/18493	0.008	TGFB1 IRF3
GO:0035306	positive regulation of dephosphorylation	2/20	56/18493	0.008	TGFB1 SMAD3
GO:0045843	negative regulation of striated muscle tissue development	2/20	56/18493	0.008	TGFB1 GSK3A
GO:0046605	regulation of centrosome cycle	2/20	56/18493	0.008	ATF5 NPM1
GO:1903749	positive regulation of establishment of protein localization to mitochondrion	2/20	56/18493	0.008	BCL2 GSK3A
GO:0007160	cell-matrix adhesion	3/20	222/18493	0.008	SMAD3 BCL2 GSK3B
GO:0045137	development of primary sexual characteristics	3/20	222/18493	0.008	PHB BCL2L1 BCL2
GO:2001234	negative regulation of apoptotic signaling pathway	3/20	222/18493	0.008	BCL2L1 MCL1 BCL2
GO:0002066	columnar/cuboidal epithelial cell development	2/20	57/18493	0.008	GSK3A GSK3B
GO:0042093	T-helper cell differentiation	2/20	57/18493	0.008	STAT3 IL6
GO:0048635	negative regulation of muscle organ development	2/20	57/18493	0.008	TGFB1 GSK3A

ID	Description	GeneRatio	BgRatio	p.adjust	geneID
GO:0000302	response to reactive oxygen species	3/20	224/18493	0.008	JUN BCL2 IL6
GO:0030520	intracellular estrogen receptor signaling pathway	2/20	58/18493	0.008	SKP2 PARP1
GO:0043388	positive regulation of DNA binding	2/20	58/18493	0.008	TGFB1 PARP1
GO:1901862	negative regulation of muscle tissue development	2/20	58/18493	0.008	TGFB1 GSK3A
GO:2000378	negative regulation of reactive oxygen species metabolic process	2/20	58/18493	0.008	STAT3 BCL2
GO:0002294	CD4-positive, alpha-beta T cell differentiation involved in immune response	2/20	59/18493	0.009	STAT3 IL6
GO:0001658	branching involved in ureteric bud morphogenesis	2/20	60/18493	0.009	TGFB1 BCL2
GO:0002287	alpha-beta T cell activation involved in immune response	2/20	60/18493	0.009	STAT3 IL6
GO:0002293	alpha-beta T cell differentiation involved in immune response	2/20	60/18493	0.009	STAT3 IL6
GO:0090092	regulation of transmembrane receptor protein serine/threonine kinase signaling pathway	3/20	235/18493	0.009	TGFB1 SMAD3 PARP1
GO:0009152	purine ribonucleotide biosynthetic process	3/20	239/18493	0.01	STAT3 TGFB1 PARP1
GO:0002637	regulation of immunoglobulin production	2/20	63/18493	0.01	TGFB1 IL6
GO:0009755	hormone-mediated signaling pathway	3/20	240/18493	0.01	PHB SKP2 PARP1
GO:0002260	lymphocyte homeostasis	2/20	64/18493	0.01	TGFB1 BCL2
GO:0042306	regulation of protein import into nucleus	2/20	64/18493	0.01	TGFB1 SMAD3
GO:1905207	regulation of cardiocyte differentiation	2/20	64/18493	0.01	TGFB1 GSK3A
GO:0019318	hexose metabolic process	3/20	244/18493	0.01	PPP1CA GSK3A GSK3B
GO:0050730	regulation of peptidyl-tyrosine phosphorylation	3/20	245/18493	0.01	STAT3 TGFB1 IL6
GO:0070374	positive regulation of ERK1 and ERK2 cascade	3/20	245/18493	0.01	PHB JUN TGFB1
GO:0051926	negative regulation of calcium ion transport	2/20	65/18493	0.01	TGFB1 BCL2
GO:0060675	ureteric bud morphogenesis	2/20	65/18493	0.01	TGFB1 BCL2
GO:1904888	cranial skeletal system development	2/20	65/18493	0.01	TGFB1 SMAD3

PATHWAY ENRICHMENT:

1 HCOV-HOST_SET

1.1 KEGG PATHWAY ENRICHMENT

BARPLOT

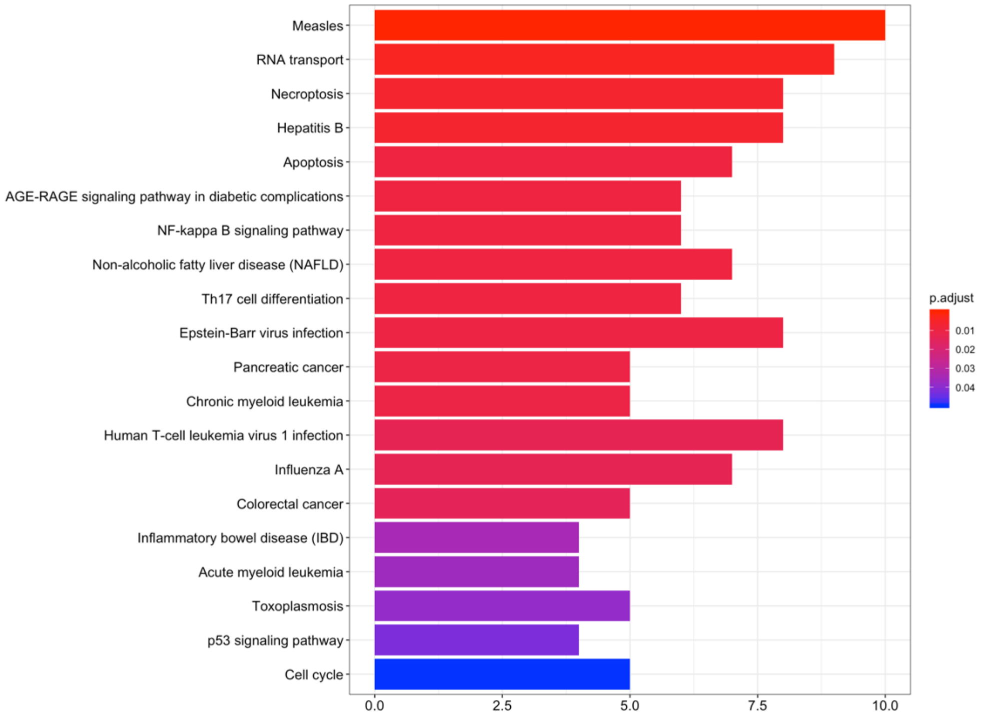

NETPLOT

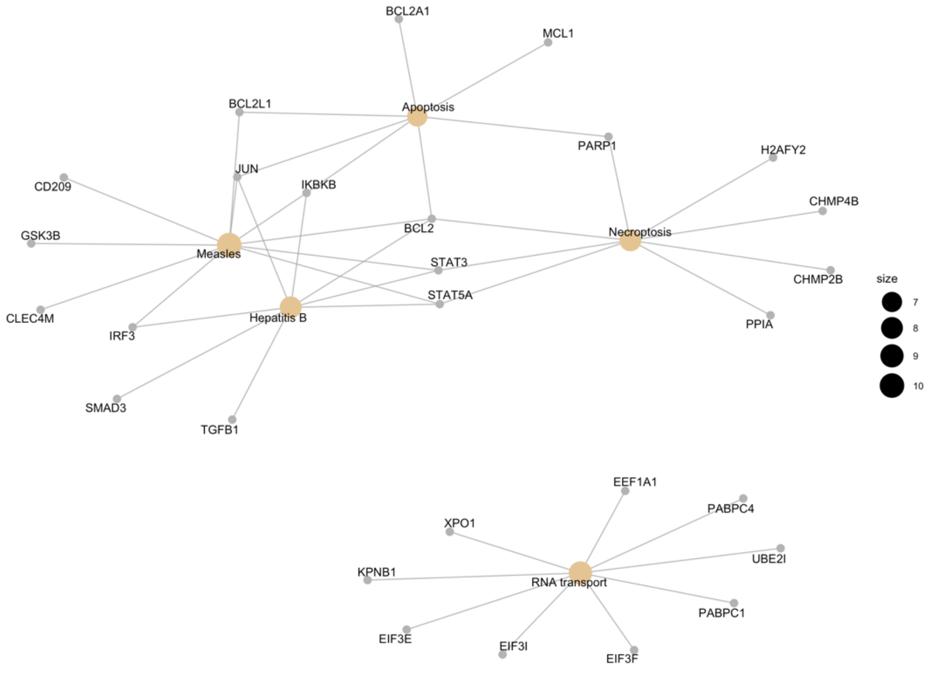

ID	Description	GeneRatio	BgRatio	p.adjust	geneID
hsa05162	Measles	10/76	138/8018	0	STAT5A JUN STAT3 CD209 CLEC4M IRF3 BCL2L1 BCL2 IKBKB GSK3B
hsa03013	RNA transport	9/76	179/8018	0.004	KPNB1 XPO1 EIF3E EIF3I EIF3F PABPC1 PABPC4 EEF1A1 UBE2I
hsa04217	Necroptosis	8/76	159/8018	0.006	STAT5A STAT3 PPIA BCL2 CHMP4B PARP1 CHMP2B H2AFY2
hsa05161	Hepatitis B	8/76	162/8018	0.006	STAT5A JUN STAT3 TGFB1 SMAD3 IRF3 BCL2 IKBKB
hsa04210	Apoptosis	7/76	136/8018	0.01	JUN BCL2L1 MCL1 BCL2A1 BCL2 IKBKB PARP1
hsa04933	AGE-RAGE signaling pathway in diabetic complications	6/76	100/8018	0.01	STAT5A JUN STAT3 TGFB1 SMAD3 BCL2
hsa04064	NF-kappa B signaling pathway	6/76	104/8018	0.01	BCL2L1 BCL2A1 BCL2 IKBKB PARP1 UBE2I
hsa04932	Non-alcoholic fatty liver disease (NAFLD)	7/76	149/8018	0.01	JUN TGFB1 COX2 IKBKB GSK3A GSK3B NDUFA10
hsa04659	Th17 cell differentiation	6/76	107/8018	0.01	STAT5A JUN STAT3 TGFB1 SMAD3 IKBKB
hsa05169	Epstein-Barr virus infection	8/76	201/8018	0.01	PSMC2 JUN STAT3 IRF3 BCL2 SKP2 IKBKB PSMD1
hsa05212	Pancreatic cancer	5/76	76/8018	0.01	STAT3 TGFB1 SMAD3 BCL2L1 IKBKB
hsa05220	Chronic myeloid leukemia	5/76	76/8018	0.01	STAT5A TGFB1 SMAD3 BCL2L1 IKBKB
hsa05166	Human T-cell leukemia virus 1 infection	8/76	219/8018	0.013	STAT5A JUN XPO1 TGFB1 SMAD3 BCL2L1 IKBKB CHEK2
hsa05164	Influenza A	7/76	170/8018	0.013	XPO1 IRF3 KPNA2 TMPRSS2 IKBKB DNAJB1 TPSAB1
hsa05210	Colorectal cancer	5/76	86/8018	0.015	JUN TGFB1 SMAD3 BCL2 GSK3B
hsa05321	Inflammatory bowel disease (IBD)	4/76	65/8018	0.035	JUN STAT3 TGFB1 SMAD3
hsa05221	Acute myeloid leukemia	4/76	67/8018	0.036	STAT5A STAT3 BCL2A1 IKBKB
hsa05145	Toxoplasmosis	5/76	112/8018	0.039	STAT3 TGFB1 BCL2L1 BCL2 IKBKB
hsa04115	p53 signaling pathway	4/76	72/8018	0.042	BCL2L1 BCL2 RRM2 CHEK2
hsa04110	Cell cycle	5/76	124/8018	0.05	TGFB1 SMAD3 SKP2 GSK3B CHEK2
hsa01521	EGFR tyrosine kinase inhibitor resistance	4/76	79/8018	0.05	STAT3 BCL2L1 BCL2 GSK3B
hsa03018	RNA degradation	4/76	79/8018	0.05	HSPD1 HSPA9 PABPC1 PABPC4
hsa05131	Shigellosis	7/76	236/8018	0.05	JUN IRF3 BCL2L1 BCL2 IKBKB GSK3A GSK3B
hsa05152	Tuberculosis	6/76	180/8018	0.05	TGFB1 CD209 CLEC4M BCL2 HSPD1 HSPA9

1.2 WIKI PATHWAY

BARPLOT

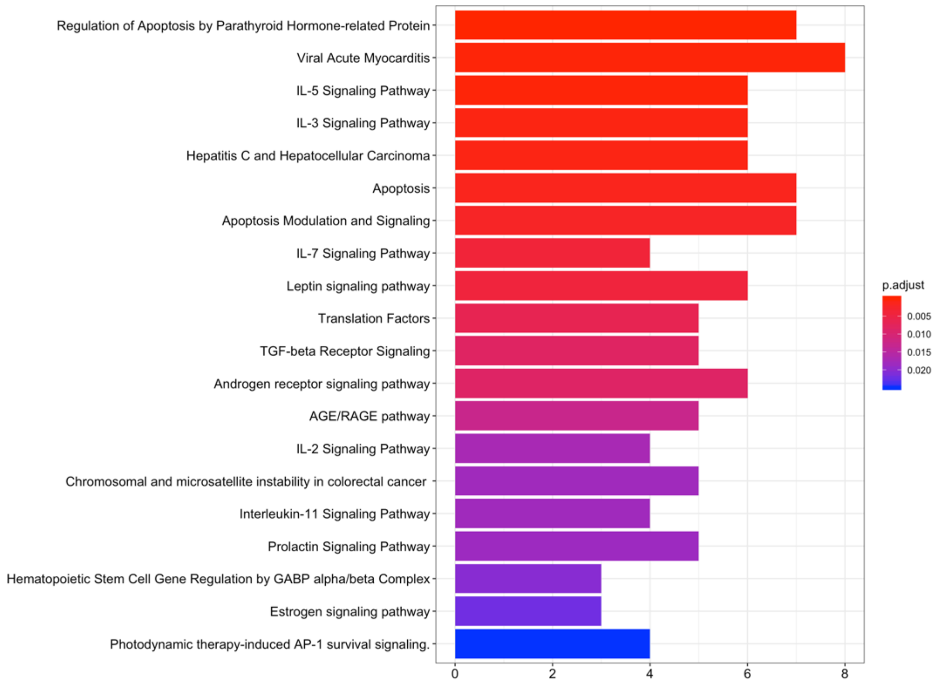

NETPLOT

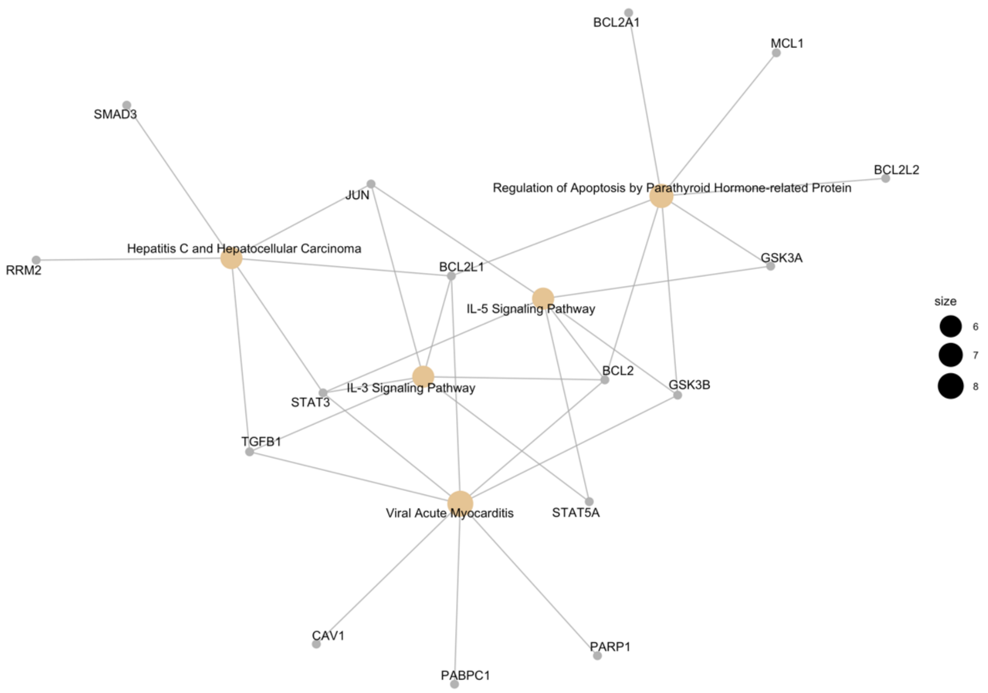

ID	Description	GeneRatio	BgRatio	p.adjust	geneID
WP3872	Regulation of Apoptosis by Parathyroid Hormone-related Protein	7/70	22/6249	0	BCL2L1 BCL2L2 MCL1 BCL2A1 BCL2 GSK3A GSK3B
WP4298	Viral Acute Myocarditis	8/70	85/6249	0	STAT3 TGFB1 BCL2L1 BCL2 GSK3B PABPC1 PARP1 CAV1
WP127	IL-5 Signaling Pathway	6/70	40/6249	0	STAT5A JUN STAT3 BCL2 GSK3A GSK3B
WP286	IL-3 Signaling Pathway	6/70	49/6249	0.001	STAT5A JUN STAT3 TGFB1 BCL2L1 BCL2
WP3646	Hepatitis C and Hepatocellular Carcinoma	6/70	51/6249	0.001	JUN STAT3 TGFB1 SMAD3 BCL2L1 RRM2
WP254	Apoptosis	7/70	86/6249	0.001	JUN IRF3 BCL2L1 BCL2L2 MCL1 BCL2 IKBKB
WP1772	Apoptosis Modulation and Signaling	7/70	94/6249	0.002	JUN BCL2L1 BCL2L2 MCL1 BCL2A1 BCL2 IKBKB
WP205	IL-7 Signaling Pathway	4/70	25/6249	0.004	STAT5A STAT3 BCL2L1 GSK3B
WP2034	Leptin signaling pathway	6/70	76/6249	0.004	STAT3 BCL2L1 KPNA4 IKBKB GSK3A GSK3B
WP107	Translation Factors	5/70	54/6249	0.006	EIF3E EIF3I EIF3F PABPC1 EEF1A1
WP560	TGF-beta Receptor Signaling	5/70	58/6249	0.008	JUN STAT3 TGFB1 SMAD3 FKBP1A
WP138	Androgen receptor signaling pathway	6/70	91/6249	0.009	JUN STAT3 SMAD3 GSK3B CAV1 UBE2I
WP2324	AGE/RAGE pathway	5/70	66/6249	0.013	STAT5A JUN STAT3 SMAD3 IKBKB
WP49	IL-2 Signaling Pathway	4/70	42/6249	0.017	STAT5A JUN STAT3 BCL2
WP4216	Chromosomal and microsatellite instability in colorectal cancer	5/70	74/6249	0.018	JUN TGFB1 SMAD3 BCL2 GSK3B
WP2332	Interleukin-11 Signaling Pathway	4/70	44/6249	0.018	STAT3 TGFB1 BCL2 IKBKB
WP2037	Prolactin Signaling Pathway	5/70	76/6249	0.018	STAT5A JUN STAT3 PPIA GSK3B
WP3657	Hematopoietic Stem Cell Gene Regulation by GABP alpha/beta Complex	3/70	22/6249	0.02	BCL2L1 MCL1 BCL2
WP712	Estrogen signaling pathway	3/70	23/6249	0.022	JUN BCL2 IKBKB
WP3611	Photodynamic therapy-induced AP-1 survival signaling.	4/70	51/6249	0.025	JUN BCL2L1 MCL1 BCL2
WP4263	Pancreatic adenocarcinoma pathway	5/70	89/6249	0.029	STAT3 TGFB1 SMAD3 BCL2L1 IKBKB
WP4217	Ebola Virus Pathway on Host	6/70	129/6249	0.029	CLEC4G CD209 CLEC4M IRF3 PRKRA CAV1
WP366	TGF-beta Signaling Pathway	6/70	133/6249	0.031	JUN TGFB1 SMAD3 HGS CAV1 UBE2I

ID	Description	GeneRatio	BgRatio	p.adjust	geneID
WP411	mRNA Processing	6/70	133/6249	0.031	HNRNPA1 PTBP1 HNRNPA2B1 SRP54 NONO DDX1
WP3972	PDGFR-beta pathway	3/70	29/6249	0.033	STAT5A JUN STAT3
WP3888	VEGFA-VEGFR2 Signaling Pathway	8/70	238/6249	0.037	JUN STAT3 BCL2L1 BCL2 GSK3B NCL HGS CAV1
WP2112	IL17 signaling pathway	3/70	31/6249	0.037	STAT3 IKBKB GSK3B
WP615	Senescence and Autophagy in Cancer	5/70	106/6249	0.047	JUN TGFB1 SMAD3 BCL2 GSK3B
WP3617	Photodynamic therapy-induced NF-kB survival signaling	3/70	35/6249	0.048	BCL2L2 BCL2A1 IKBKB

2 HCOV-CLUSTER_SET

2.1 KEGG PATHWAY ENRICHMENT

BARPLOT

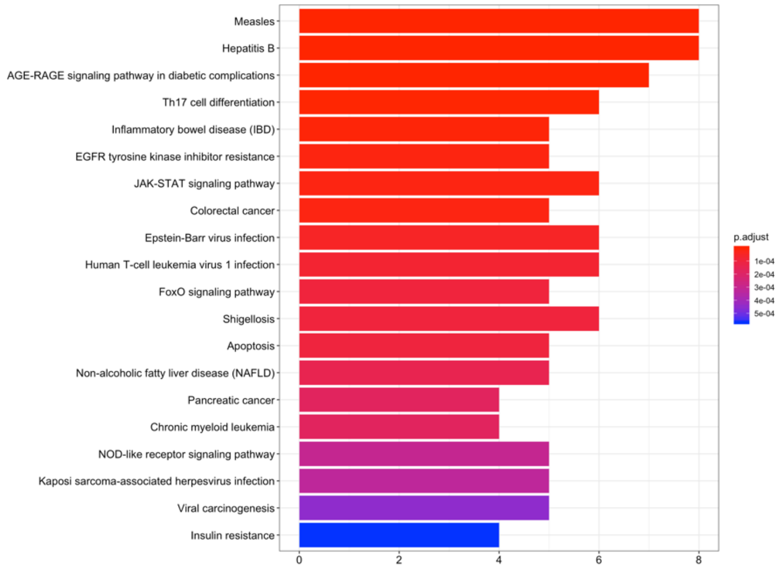

NETPLOT

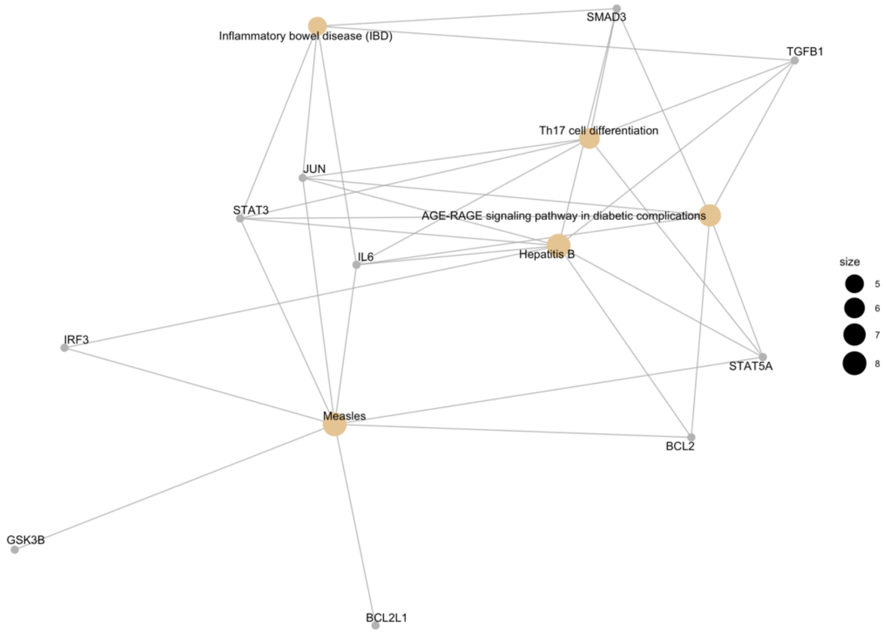

ID	Description	GeneRatio	BgRatio	p.adjust	geneID
hsa05162	Measles	8/18	138/8018	0	STAT5A JUN STAT3 IRF3 BCL2L1 BCL2 GSK3B IL6
hsa05161	Hepatitis B	8/18	162/8018	0	STAT5A JUN STAT3 TGFB1 SMAD3 IRF3 BCL2 IL6
hsa04933	AGE-RAGE signaling pathway in diabetic complications	7/18	100/8018	0	STAT5A JUN STAT3 TGFB1 SMAD3 BCL2 IL6
hsa04659	Th17 cell differentiation	6/18	107/8018	0	STAT5A JUN STAT3 TGFB1 SMAD3 IL6
hsa05321	Inflammatory bowel disease (IBD)	5/18	65/8018	0	JUN STAT3 TGFB1 SMAD3 IL6
hsa01521	EGFR tyrosine kinase inhibitor resistance	5/18	79/8018	0	STAT3 BCL2L1 BCL2 GSK3B IL6
hsa04630	JAK-STAT signaling pathway	6/18	162/8018	0	STAT5A STAT3 BCL2L1 MCL1 BCL2 IL6
hsa05210	Colorectal cancer	5/18	86/8018	0	JUN TGFB1 SMAD3 BCL2 GSK3B
hsa05169	Epstein-Barr virus infection	6/18	201/8018	0	JUN STAT3 IRF3 BCL2 SKP2 IL6
hsa05166	Human T-cell leukemia virus 1 infection	6/18	219/8018	0	STAT5A JUN TGFB1 SMAD3 BCL2L1 IL6
hsa04068	FoxO signaling pathway	5/18	131/8018	0	STAT3 TGFB1 SMAD3 SKP2 IL6
hsa05131	Shigellosis	6/18	236/8018	0	JUN IRF3 BCL2L1 BCL2 GSK3A GSK3B
hsa04210	Apoptosis	5/18	136/8018	0	JUN BCL2L1 MCL1 BCL2 PARP1
hsa04932	Non-alcoholic fatty liver disease (NAFLD)	5/18	149/8018	0	JUN TGFB1 GSK3A GSK3B IL6
hsa05212	Pancreatic cancer	4/18	76/8018	0	STAT3 TGFB1 SMAD3 BCL2L1
hsa05220	Chronic myeloid leukemia	4/18	76/8018	0	STAT5A TGFB1 SMAD3 BCL2L1
hsa04621	NOD-like receptor signaling pathway	5/18	181/8018	0	JUN IRF3 BCL2L1 BCL2 IL6
hsa05167	Kaposi sarcoma-associated herpesvirus infection	5/18	186/8018	0	JUN STAT3 IRF3 GSK3B IL6
hsa05203	Viral carcinogenesis	5/18	201/8018	0	STAT5A JUN STAT3 IRF3 SKP2
hsa04931	Insulin resistance	4/18	108/8018	0.001	STAT3 PPP1CA GSK3B IL6
hsa05145	Toxoplasmosis	4/18	112/8018	0.001	STAT3 TGFB1 BCL2L1 BCL2
hsa05135	Yersinia infection	4/18	120/8018	0.001	JUN IRF3 GSK3B IL6
hsa04110	Cell cycle	4/18	124/8018	0.001	TGFB1 SMAD3 SKP2 GSK3B
hsa05226	Gastric cancer	4/18	149/8018	0.002	TGFB1 SMAD3 BCL2 GSK3B
hsa04390	Hippo signaling pathway	4/18	157/8018	0.002	PPP1CA TGFB1 SMAD3 GSK3B
hsa04217	Necroptosis	4/18	159/8018	0.002	STAT5A STAT3 BCL2 PARP1
hsa04218	Cellular senescence	4/18	160/8018	0.002	PPP1CA TGFB1 SMAD3 IL6

ID	Description	GeneRatio	BgRatio	p.adjust	geneID
hsa04137	Mitophagy - animal	3/18	65/8018	0.002	TFEB JUN BCL2L1
hsa05225	Hepatocellular carcinoma	4/18	168/8018	0.002	TGFB1 SMAD3 BCL2L1 GSK3B
hsa04917	Prolactin signaling pathway	3/18	70/8018	0.002	STAT5A STAT3 GSK3B
hsa05133	Pertussis	3/18	76/8018	0.003	JUN IRF3 IL6
hsa04012	ErbB signaling pathway	3/18	85/8018	0.004	STAT5A JUN GSK3B
hsa04510	Focal adhesion	4/18	199/8018	0.004	JUN PPP1CA BCL2 GSK3B
hsa04151	PI3K-Akt signaling pathway	5/18	354/8018	0.004	BCL2L1 MCL1 BCL2 GSK3B IL6
hsa05222	Small cell lung cancer	3/18	92/8018	0.004	BCL2L1 BCL2 SKP2
hsa05170	Human immunodeficiency virus 1 infection	4/18	212/8018	0.004	JUN IRF3 BCL2L1 BCL2
hsa05323	Rheumatoid arthritis	3/18	93/8018	0.004	JUN TGFB1 IL6
hsa04657	IL-17 signaling pathway	3/18	94/8018	0.004	JUN GSK3B IL6
hsa05163	Human cytomegalovirus infection	4/18	225/8018	0.005	STAT3 IRF3 GSK3B IL6
hsa05142	Chagas disease (American trypanosomiasis)	3/18	102/8018	0.005	JUN TGFB1 IL6
hsa04064	NF-kappa B signaling pathway	3/18	104/8018	0.005	BCL2L1 BCL2 PARP1
hsa04620	Toll-like receptor signaling pathway	3/18	104/8018	0.005	JUN IRF3 IL6
hsa04066	HIF-1 signaling pathway	3/18	109/8018	0.006	STAT3 BCL2 IL6
hsa04722	Neurotrophin signaling pathway	3/18	119/8018	0.007	JUN BCL2 GSK3B
hsa04935	Growth hormone synthesis, secretion and action	3/18	119/8018	0.007	STAT5A STAT3 GSK3B
hsa04215	Apoptosis - multiple species	2/18	32/8018	0.007	BCL2L1 BCL2
hsa04728	Dopaminergic synapse	3/18	132/8018	0.009	PPP1CA GSK3A GSK3B
hsa04550	Signaling pathways regulating pluripotency of stem cells	3/18	142/8018	0.01	STAT3 SMAD3 GSK3B
hsa05168	Herpes simplex virus 1 infection	5/18	491/8018	0.01	PPP1CA IRF3 BCL2L1 BCL2 IL6
hsa05160	Hepatitis C	3/18	155/8018	0.013	STAT3 IRF3 GSK3B
hsa04310	Wnt signaling pathway	3/18	160/8018	0.014	JUN SMAD3 GSK3B
hsa04672	Intestinal immune network for IgA production	2/18	49/8018	0.014	TGFB1 IL6
hsa04340	Hedgehog signaling pathway	2/18	50/8018	0.014	BCL2 GSK3B
hsa05144	Malaria	2/18	50/8018	0.014	TGFB1 IL6
hsa05014	Amyotrophic lateral sclerosis (ALS)	2/18	57/8018	0.017	BCL2L1 BCL2
hsa05152	Tuberculosis	3/18	180/8018	0.017	TGFB1 BCL2 IL6

ID	Description	GeneRatio	BgRatio	p.adjust	geneID
hsa04062	Chemokine signaling pathway	3/18	189/8018	0.02	STAT3 GSK3A GSK3B
hsa04623	Cytosolic DNA-sensing pathway	2/18	63/8018	0.02	IRF3 IL6
hsa05223	Non-small cell lung cancer	2/18	66/8018	0.022	STAT5A STAT3
hsa05221	Acute myeloid leukemia	2/18	67/8018	0.022	STAT5A STAT3
hsa05205	Proteoglycans in cancer	3/18	204/8018	0.022	STAT3 PPP1CA TGFB1
hsa05031	Amphetamine addiction	2/18	69/8018	0.022	JUN PPP1CA
hsa05211	Renal cell carcinoma	2/18	69/8018	0.022	JUN TGFB1
hsa04115	p53 signaling pathway	2/18	72/8018	0.024	BCL2L1 BCL2
hsa05132	Salmonella infection	3/18	214/8018	0.024	JUN BCL2 IL6
hsa01524	Platinum drug resistance	2/18	73/8018	0.024	BCL2L1 BCL2
hsa05140	Leishmaniasis	2/18	77/8018	0.026	JUN TGFB1
hsa04662	B cell receptor signaling pathway	2/18	82/8018	0.029	JUN GSK3B
hsa05235	PD-L1 expression and PD-1 checkpoint pathway in cancer	2/18	89/8018	0.033	JUN STAT3
hsa05410	Hypertrophic cardiomyopathy (HCM)	2/18	90/8018	0.033	TGFB1 IL6
hsa04658	Th1 and Th2 cell differentiation	2/18	92/8018	0.034	STAT5A JUN
hsa04350	TGF-beta signaling pathway	2/18	94/8018	0.035	TGFB1 SMAD3
hsa05215	Prostate cancer	2/18	97/8018	0.037	BCL2 GSK3B
hsa01522	Endocrine resistance	2/18	98/8018	0.037	JUN BCL2
hsa05146	Amoebiasis	2/18	102/8018	0.04	TGFB1 IL6
hsa04625	C-type lectin receptor signaling pathway	2/18	104/8018	0.04	JUN IL6
hsa04660	T cell receptor signaling pathway	2/18	104/8018	0.04	JUN GSK3B
hsa04928	Parathyroid hormone synthesis, secretion and action	2/18	106/8018	0.041	BCL2 NACA
hsa04668	TNF signaling pathway	2/18	112/8018	0.045	JUN IL6

2.2 WIKI PATHWAY

BARPLOT

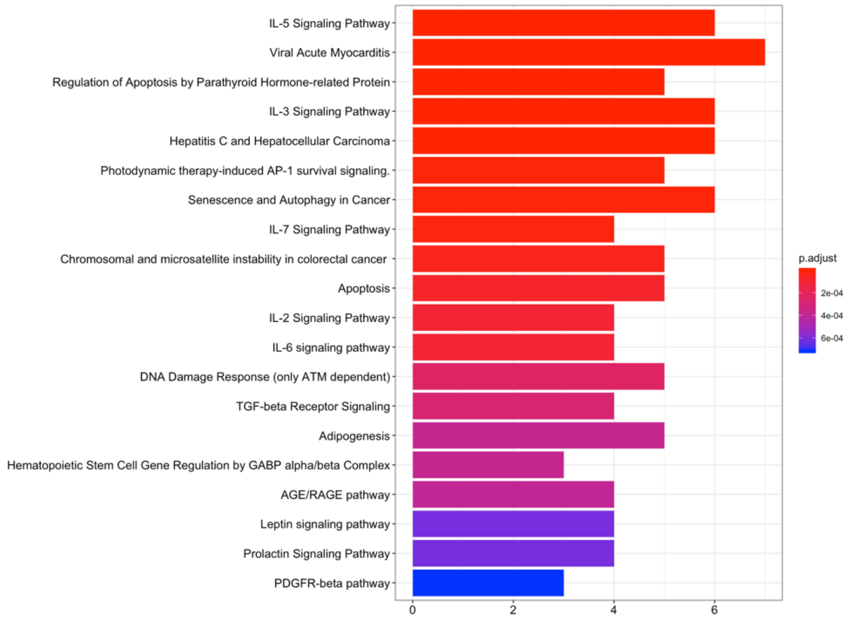

NETPLOT

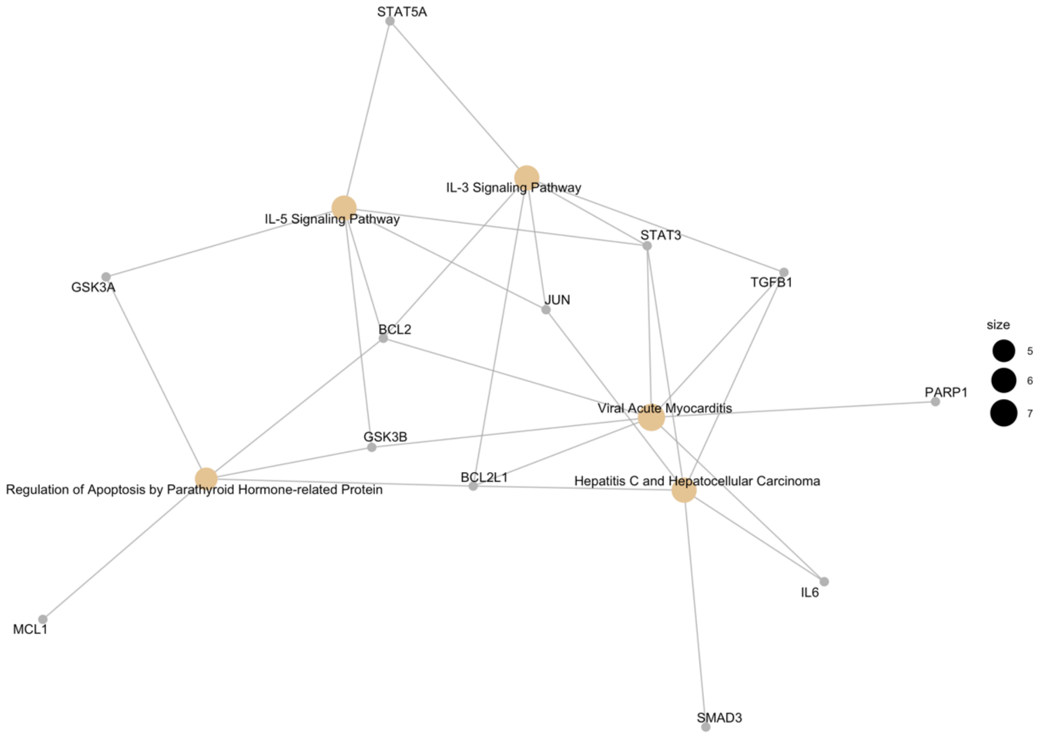

ID	Description	GeneRatio	BgRatio	p.adjust	geneID
WP127	IL-5 Signaling Pathway	6/19	40/6249	0	STAT5A JUN STAT3 BCL2 GSK3A GSK3B
WP4298	Viral Acute Myocarditis	7/19	85/6249	0	STAT3 TGFB1 BCL2L1 BCL2 GSK3B PARP1 IL6
WP3872	Regulation of Apoptosis by Parathyroid Hormone-related Protein	5/19	22/6249	0	BCL2L1 MCL1 BCL2 GSK3A GSK3B
WP286	IL-3 Signaling Pathway	6/19	49/6249	0	STAT5A JUN STAT3 TGFB1 BCL2L1 BCL2
WP3646	Hepatitis C and Hepatocellular Carcinoma	6/19	51/6249	0	JUN STAT3 TGFB1 SMAD3 BCL2L1 IL6
WP3611	Photodynamic therapy-induced AP-1 survival signaling.	5/19	51/6249	0	JUN BCL2L1 MCL1 BCL2 IL6
WP615	Senescence and Autophagy in Cancer	6/19	106/6249	0	JUN TGFB1 SMAD3 BCL2 GSK3B IL6
WP205	IL-7 Signaling Pathway	4/19	25/6249	0	STAT5A STAT3 BCL2L1 GSK3B
WP4216	Chromosomal and microsatellite instability in colorectal cancer	5/19	74/6249	0	JUN TGFB1 SMAD3 BCL2 GSK3B
WP254	Apoptosis	5/19	86/6249	0	JUN IRF3 BCL2L1 MCL1 BCL2
WP49	IL-2 Signaling Pathway	4/19	42/6249	0	STAT5A JUN STAT3 BCL2
WP364	IL-6 signaling pathway	4/19	43/6249	0	STAT3 BCL2L1 GSK3B IL6
WP710	DNA Damage Response (only ATM dependent)	5/19	115/6249	0	JUN TGFB1 SMAD3 BCL2 GSK3B
WP560	TGF-beta Receptor Signaling	4/19	58/6249	0	JUN STAT3 TGFB1 SMAD3
WP236	Adipogenesis	5/19	131/6249	0	STAT5A STAT3 TGFB1 SMAD3 IL6
WP3657	Hematopoietic Stem Cell Gene Regulation by GABP alpha/beta Complex	3/19	22/6249	0	BCL2L1 MCL1 BCL2
WP2324	AGE/RAGE pathway	4/19	66/6249	0	STAT5A JUN STAT3 SMAD3
WP2034	Leptin signaling pathway	4/19	76/6249	0.001	STAT3 BCL2L1 GSK3A GSK3B
WP2037	Prolactin Signaling Pathway	4/19	76/6249	0.001	STAT5A JUN STAT3 GSK3B
WP3972	PDGFR-beta pathway	3/19	29/6249	0.001	STAT5A JUN STAT3
WP4263	Pancreatic adenocarcinoma pathway	4/19	89/6249	0.001	STAT3 TGFB1 SMAD3 BCL2L1
WP138	Androgen receptor signaling pathway	4/19	91/6249	0.001	JUN STAT3 SMAD3 GSK3B

ID	Description	GeneRatio	BgRatio	p.adjust	geneID
WP2355	Corticotropin-releasing hormone signaling pathway	4/19	93/6249	0.001	TGFB1 BCL2 GSK3B PARP1
WP1772	Apoptosis Modulation and Signaling	4/19	94/6249	0.001	JUN BCL2L1 MCL1 BCL2
WP2036	TNF related weak inducer of apoptosis (TWEAK) Signaling Pathway	3/19	42/6249	0.002	JUN GSK3B IL6
WP2332	Interleukin-11 Signaling Pathway	3/19	44/6249	0.002	STAT3 TGFB1 BCL2
WP314	Fas Ligand (FasL) pathway and Stress induction of Heat Shock Proteins (HSP) regulation	3/19	44/6249	0.002	JUN BCL2 PARP1
WP3594	Circadian rythm related genes	5/19	207/6249	0.002	JUN PPP1CA ATF5 GSK3B IL6
WP2203	Thymic Stromal LymphoPoietin (TSLP) Signaling Pathway	3/19	47/6249	0.002	STAT5A STAT3 IL6
WP179	Cell Cycle	4/19	122/6249	0.002	TGFB1 SMAD3 SKP2 GSK3B
WP673	ErbB Signaling Pathway	3/19	54/6249	0.003	STAT5A JUN GSK3B
WP1559	TFs Regulate miRNAs related to cardiac hypertrophy	2/19	12/6249	0.003	STAT3 TGFB1
WP3888	VEGFA-VEGFR2 Signaling Pathway	5/19	238/6249	0.003	JUN STAT3 BCL2L1 BCL2 GSK3B
WP2881	Estrogen Receptor Pathway	2/19	13/6249	0.003	JUN STAT3
WP304	Kit receptor signaling pathway	3/19	60/6249	0.004	STAT5A STAT3 BCL2
WP2380	Brain-Derived Neurotrophic Factor (BDNF) signaling pathway	4/19	144/6249	0.004	STAT5A JUN STAT3 GSK3B
WP1984	Integrated Breast Cancer Pathway	4/19	154/6249	0.005	PHB JUN BCL2 GSK3A
WP3680	Association Between Physico-Chemical Features and Toxicity Associated Pathways	3/19	68/6249	0.005	STAT5A JUN GSK3B
WP3874	Canonical and Non-Canonical TGF-B signaling	2/19	17/6249	0.005	TGFB1 SMAD3
WP3859	TGF-B Signaling in Thyroid Cells for Epithelial-Mesenchymal Transition	2/19	18/6249	0.006	TGFB1 SMAD3
WP22	IL-9 Signaling Pathway	2/19	19/6249	0.006	STAT5A STAT3
WP3287	Overview of nanoparticle effects	2/19	19/6249	0.006	BCL2 IL6
WP2507	Nanomaterial induced apoptosis	2/19	21/6249	0.007	BCL2L1 MCL1
WP712	Estrogen signaling pathway	2/19	23/6249	0.008	JUN BCL2
WP69	T-Cell antigen Receptor (TCR) Signaling Pathway	3/19	91/6249	0.009	JUN TGFB1 IL6
WP1528	Physiological and Pathological Hypertrophy of the Heart	2/19	25/6249	0.009	JUN STAT3
WP231	TNF alpha Signaling Pathway	3/19	94/6249	0.01	JUN BCL2L1 IL6
WP581	EPO Receptor Signaling	2/19	26/6249	0.01	STAT5A STAT3

ID	Description	GeneRatio	BgRatio	p.adjust	geneID
WP306	Focal Adhesion	4/19	202/6249	0.01	JUN PPP1CA BCL2 GSK3B
WP530	Cytokines and Inflammatory Response	2/19	27/6249	0.01	TGFB1 IL6
WP23	B Cell Receptor Signaling Pathway	3/19	98/6249	0.01	JUN GSK3A GSK3B
WP4172	PI3K-Akt Signaling Pathway	5/19	345/6249	0.01	BCL2L1 MCL1 BCL2 GSK3B IL6
WP4300	Extracellular vesicles in the crosstalk of cardiac cells	2/19	28/6249	0.01	STAT3 IL6
WP1544	MicroRNAs in cardiomyocyte hypertrophy	3/19	102/6249	0.01	STAT3 TGFB1 GSK3B
WP2583	T-Cell Receptor and Co-stimulatory Signaling	2/19	29/6249	0.01	GSK3A GSK3B
WP75	Toll-like Receptor Signaling Pathway	3/19	103/6249	0.01	JUN IRF3 IL6
WP2855	Dopaminergic Neurogenesis	2/19	30/6249	0.01	STAT3 TGFB1
WP2870	Extracellular vesicle-mediated signaling in recipient cells	2/19	30/6249	0.01	TGFB1 SMAD3
WP3844	PI3K-AKT-mTOR signaling pathway and therapeutic opportunities	2/19	30/6249	0.01	TFEB GSK3B
WP3851	TLR4 Signaling and Tolerance	2/19	30/6249	0.01	IRF3 IL6
WP2112	IL17 signaling pathway	2/19	31/6249	0.011	STAT3 GSK3B
WP3850	Factors and pathways affecting insulin-like growth factor (IGF1)-Akt signaling	2/19	31/6249	0.011	SMAD3 GSK3B
WP4149	White fat cell differentiation	2/19	33/6249	0.012	STAT5A IRF3
WP313	Signaling of Hepatocyte Growth Factor Receptor	2/19	34/6249	0.013	JUN STAT3
WP4241	Type 2 papillary renal cell carcinoma	2/19	36/6249	0.014	TFEB TGFB1
WP3931	ESC Pluripotency Pathways	3/19	119/6249	0.014	JUN STAT3 GSK3B
WP3614	Photodynamic therapy-induced HIF-1 survival signaling	2/19	37/6249	0.014	BCL2L1 MCL1
WP3995	Prion disease pathway	2/19	37/6249	0.014	STAT3 BCL2
WP2447	Amyotrophic lateral sclerosis (ALS)	2/19	38/6249	0.014	BCL2L1 BCL2
WP4136	Fibrin Complement Receptor 3 Signaling Pathway	2/19	38/6249	0.014	IRF3 IL6
WP2526	PDGF Pathway	2/19	39/6249	0.015	JUN STAT3
WP500	Glycogen Metabolism	2/19	40/6249	0.015	GSK3A GSK3B
WP366	TGF-beta Signaling Pathway	3/19	133/6249	0.017	JUN TGFB1 SMAD3
WP1971	Integrated Cancer Pathway	2/19	46/6249	0.02	SMAD3 BCL2
WP1449	Regulation of toll-like receptor signaling pathway	3/19	145/6249	0.02	JUN IRF3 IL6
WP2848	Differentiation Pathway	2/19	48/6249	0.02	TGFB1 IL6
WP2873	Aryl Hydrocarbon Receptor Pathway	2/19	48/6249	0.02	JUN TGFB1

ID	Description	GeneRatio	BgRatio	p.adjust	geneID
WP2038	Regulation of Microtubule Cytoskeleton	2/19	49/6249	0.021	STAT3 GSK3B
WP2406	Cardiac Progenitor Differentiation	2/19	53/6249	0.024	TGFB1 GSK3B
WP363	Wnt Signaling Pathway	2/19	53/6249	0.024	GSK3A GSK3B
WP4262	Breast cancer pathway	3/19	157/6249	0.024	JUN GSK3B PARP1
WP3286	Copper homeostasis	2/19	54/6249	0.024	JUN GSK3B
WP289	Myometrial Relaxation and Contraction Pathways	3/19	158/6249	0.024	JUN ATF5 IL6
WP395	IL-4 Signaling Pathway	2/19	55/6249	0.024	STAT5A STAT3
WP585	Interferon type I signaling pathways	2/19	55/6249	0.024	STAT5A STAT3
WP481	Insulin Signaling	3/19	161/6249	0.024	JUN GSK3A GSK3B
WP4239	Epithelial to mesenchymal transition in colorectal cancer	3/19	164/6249	0.025	TGFB1 SMAD3 GSK3B
WP437	EGF/EGFR Signaling Pathway	3/19	164/6249	0.025	STAT5A JUN STAT3
WP2795	Cardiac Hypertrophic Response	2/19	57/6249	0.025	TGFB1 GSK3B
WP3981	miRNA regulation of prostate cancer signaling pathways	2/19	59/6249	0.026	BCL2 GSK3B
WP4205	MET in type 1 papillary renal cell carcinoma	2/19	59/6249	0.026	JUN STAT3
WP3863	T-Cell antigen Receptor (TCR) pathway during Staphylococcus aureus infection	2/19	62/6249	0.028	JUN GSK3B
WP2849	Hematopoietic Stem Cell Differentiation	2/19	63/6249	0.028	STAT5A IL6
WP3624	Lung fibrosis	2/19	63/6249	0.028	TGFB1 IL6
WP61	Notch Signaling Pathway	2/19	63/6249	0.028	STAT3 GSK3B
WP4255	Non-small cell lung cancer	2/19	66/6249	0.03	STAT5A STAT3
WP2032	Human Thyroid Stimulating Hormone (TSH) signaling pathway	2/19	68/6249	0.032	JUN STAT3
WP3303	RAC1/PAK1/p38/MMP2 Pathway	2/19	69/6249	0.032	STAT5A STAT3
WP1982	Sterol Regulatory Element-Binding Proteins (SREBP) signalling	2/19	73/6249	0.035	KPNB1 GSK3A
WP4018	Pathways in clear cell renal cell carcinoma	2/19	87/6249	0.049	STAT3 TGFB1
WP2840	Hair Follicle Development: Cytodifferentiation (Part 3 of 3)	2/19	88/6249	0.049	JUN TGFB1

CLUSTER ANALYSIS

1 CLUSTER COMPARISON

GO

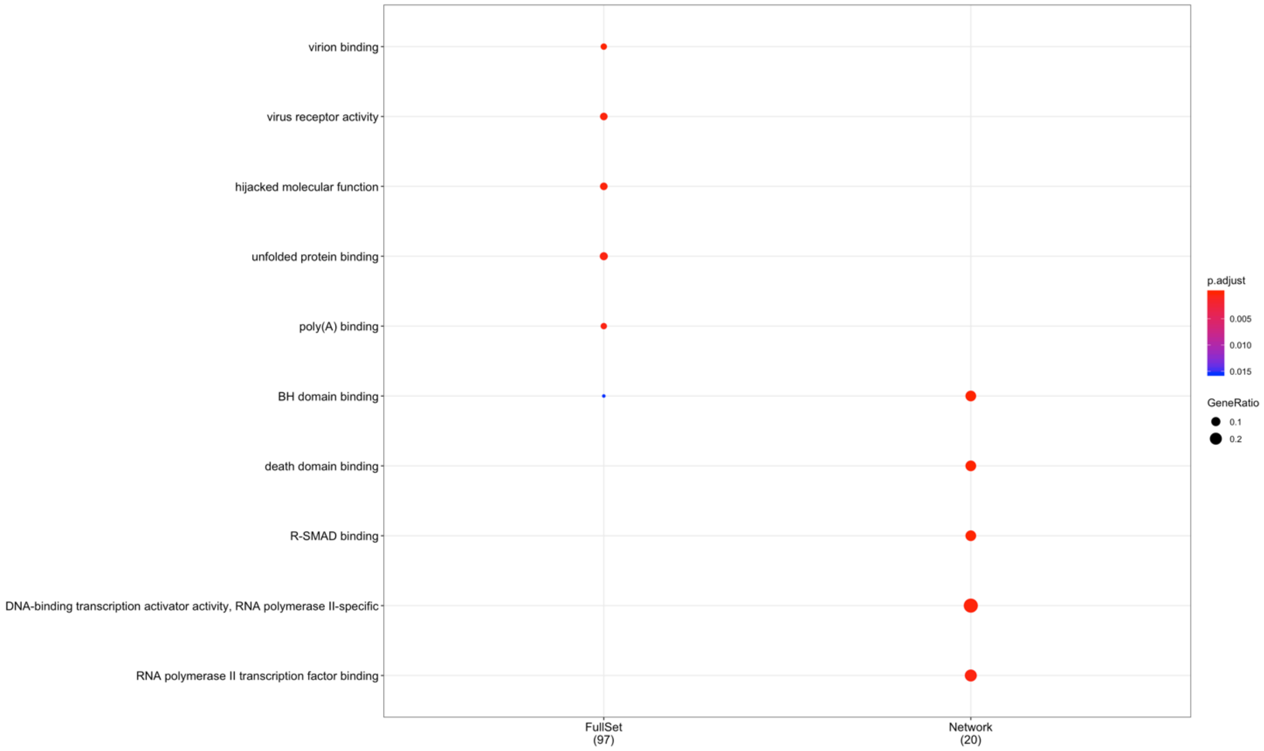

KEGG PATHWAY

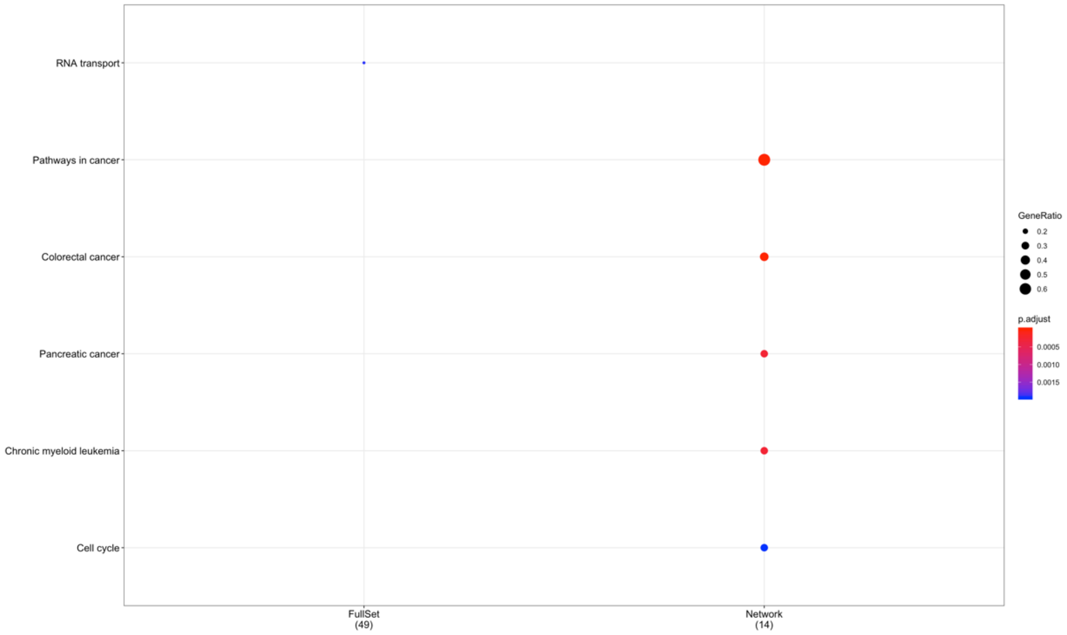

GO.LOCATION

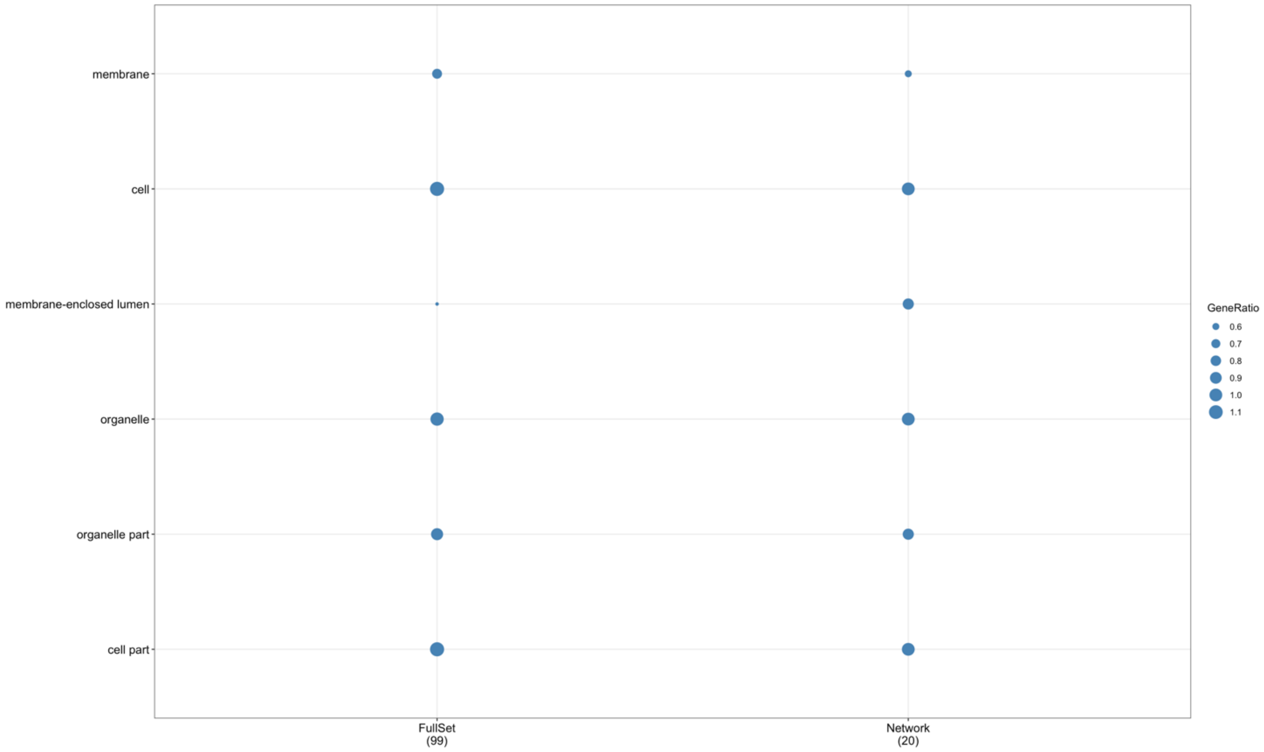

2 TABLES

2.1 GO

Cluster	group	ID	Description	GeneRatio	BgRatio	p.adjust	geneID
FullSet	FullSet	GO:0046790	virion binding	4/97	10/17632	0	30835 10332 5478 302
FullSet	FullSet	GO:0001618	virus receptor activity	6/97	74/17632	0	59272 339390 30835 10332 290 1803
FullSet	FullSet	GO:0104005	hijacked molecular function	6/97	74/17632	0	59272 339390 30835 10332 290 1803
FullSet	FullSet	GO:0051082	unfolded protein binding	7/97	127/17632	0	5478 3337 3329 3313 9360 10465 5204
FullSet	FullSet	GO:0008143	poly(A) binding	4/97	24/17632	0.001	10492 26986 8761 1653
FullSet	FullSet	GO:0003727	single-stranded RNA binding	6/97	90/17632	0.001	3178 10492 5725 26986 8761 1653
FullSet	FullSet	GO:0070717	poly-purine tract binding	4/97	30/17632	0.001	10492 26986 8761 1653
FullSet	FullSet	GO:0033218	amide binding	10/97	348/17632	0.001	91452 30835 10332 3838 5478 290 3329 6729 9360 10465
FullSet	FullSet	GO:0098505	G-rich strand telomeric DNA binding	3/97	11/17632	0.001	3178 3181 7013
FullSet	FullSet	GO:0042277	peptide binding	9/97	285/17632	0.001	30835 10332 3838 5478 290 3329 6729 9360 10465
FullSet	FullSet	GO:0043047	single-stranded telomeric DNA binding	3/97	13/17632	0.001	3178 3181 7013
FullSet	FullSet	GO:0003755	peptidyl-prolyl cis-trans isomerase activity	4/97	37/17632	0.001	5478 2280 9360 10465
FullSet	FullSet	GO:0042162	telomeric DNA binding	4/97	38/17632	0.001	3178 3181 4691 7013
FullSet	FullSet	GO:0098847	sequence-specific single stranded DNA binding	3/97	15/17632	0.001	3178 3181 7013
FullSet	FullSet	GO:0045296	cadherin binding	9/97	323/17632	0.001	11344 10652 6811 302 3337 3646 128866 51429 25978
FullSet	FullSet	GO:0016859	cis-trans isomerase activity	4/97	41/17632	0.001	5478 2280 9360 10465

Cluster	group	ID	Description	GeneRatio	BgRatio	p.adjust	geneID
FullSet	FullSet	GO:0016018	cyclosporin A binding	3/97	19/17632	0.003	5478 9360 10465
FullSet	FullSet	GO:0140142	nucleocytoplasmic carrier activity	3/97	19/17632	0.003	7514 3838 3840
FullSet	FullSet	GO:0003729	mRNA binding	10/97	463/17632	0.004	3178 3181 220988 23521 26986 8761 10146 9908 4691 1655
FullSet	FullSet	GO:0050839	cell adhesion molecule binding	10/97	486/17632	0.005	11344 10652 6811 928 302 3337 3646 128866 51429 25978
FullSet	FullSet	GO:0036002	pre-mRNA binding	4/97	62/17632	0.005	3178 5725 3181 1655
FullSet	FullSet	GO:0005484	SNAP receptor activity	3/97	30/17632	0.008	10652 6811 116841
FullSet	FullSet	GO:0008187	poly-pyrimidine tract binding	3/97	33/17632	0.01	5725 26986 8761
FullSet	FullSet	GO:0003730	mRNA 3'-UTR binding	4/97	80/17632	0.013	3181 26986 8761 1655
FullSet	FullSet	GO:0051400	BH domain binding	2/97	10/17632	0.015	599 597
FullSet	FullSet	GO:0061608	nuclear import signal receptor activity	2/97	10/17632	0.015	3838 3840
FullSet	FullSet	GO:0044389	ubiquitin-like protein ligase binding	7/97	301/17632	0.016	3329 3313 5707 51429 9146 7917 11200
FullSet	FullSet	GO:0008135	translation factor activity, RNA binding	4/97	89/17632	0.016	3646 8668 8665 1915
FullSet	FullSet	GO:0140104	molecular carrier activity	3/97	43/17632	0.018	7514 3838 3840
FullSet	FullSet	GO:0008494	translation activator activity	2/97	13/17632	0.023	26986 57409
FullSet	FullSet	GO:0003724	RNA helicase activity	3/97	48/17632	0.023	10146 1653 1655
FullSet	FullSet	GO:0003697	single-stranded DNA binding	4/97	105/17632	0.026	3178 3181 3329 7013
FullSet	FullSet	GO:0003743	translation initiation factor activity	3/97	51/17632	0.026	3646 8668 8665
FullSet	FullSet	GO:0016887	ATPase activity	8/97	445/17632	0.028	5701 3329 3313 3832 10146 1653 1655 9550
FullSet	FullSet	GO:0042623	ATPase activity, coupled	7/97	357/17632	0.031	5701 3313 3832 10146 1653 1655 9550

Cluster	group	ID	Description	GeneRatio	BgRatio	p.adjust	geneID
FullSet	FullSet	GO:0048306	calcium-dependent protein binding	3/97	62/17632	0.041	10332 302 1655
FullSet	FullSet	GO:0031625	ubiquitin protein ligase binding	6/97	286/17632	0.041	3329 3313 5707 51429 7917 11200
FullSet	FullSet	GO:0002020	protease binding	4/97	125/17632	0.041	1803 302 3329 7917
FullSet	FullSet	GO:0043021	ribonucleoprotein complex binding	4/97	128/17632	0.043	6729 10465 7917 1655
FullSet	FullSet	GO:0005537	mannose binding	2/97	22/17632	0.049	30835 10332
Network	Network	GO:0051400	BH domain binding	3/20	10/17632	0	598 4170 596
Network	Network	GO:0070513	death domain binding	3/20	10/17632	0	598 4170 596
Network	Network	GO:0070412	R-SMAD binding	3/20	23/17632	0	3725 4088 142
Network	Network	GO:0001228	DNA-binding transcription activator activity, RNA polymerase II-specific	6/20	449/17632	0	3725 6774 4088 22809 3661 142
Network	Network	GO:0001085	RNA polymerase II transcription factor binding	4/20	140/17632	0	3725 6774 4088 2932
Network	Network	GO:0019902	phosphatase binding	4/20	178/17632	0.001	6774 5499 4088 596
Network	Network	GO:0033613	activating transcription factor binding	3/20	76/17632	0.001	3725 4088 4869
Network	Network	GO:0034236	protein kinase A catalytic subunit binding	2/20	13/17632	0.001	2931 2932
Network	Network	GO:0046332	SMAD binding	3/20	80/17632	0.001	3725 4088 142
Network	Network	GO:0035259	glucocorticoid receptor binding	2/20	14/17632	0.001	6774 4088
Network	Network	GO:0050321	tau-protein kinase activity	2/20	15/17632	0.001	2931 2932
Network	Network	GO:0035258	steroid hormone receptor binding	3/20	90/17632	0.001	6774 4088 142
Network	Network	GO:0031625	ubiquitin protein ligase binding	4/20	286/17632	0.003	3725 4088 596 2932
Network	Network	GO:0044389	ubiquitin-like protein ligase binding	4/20	301/17632	0.003	3725 4088 596 2932
Network	Network	GO:0019903	protein phosphatase binding	3/20	133/17632	0.003	6774 5499 596
Network	Network	GO:0051059	NF-kappaB binding	2/20	28/17632	0.003	2932 4869
Network	Network	GO:0035257	nuclear hormone receptor binding	3/20	152/17632	0.005	6774 4088 142

Cluster	group	ID	Description	GeneRatio	BgRatio	p.adjust	geneID
Network	Network	GO:0001102	RNA polymerase II activating transcription factor binding	2/20	44/17632	0.007	3725 4088
Network	Network	GO:0051427	hormone receptor binding	3/20	185/17632	0.007	6774 4088 142
Network	Network	GO:0051018	protein kinase A binding	2/20	48/17632	0.008	2931 2932
Network	Network	GO:0005160	transforming growth factor beta receptor binding	2/20	51/17632	0.009	7040 4088
Network	Network	GO:0005126	cytokine receptor binding	3/20	281/17632	0.02	6774 7040 4088
Network	Network	GO:0008013	beta-catenin binding	2/20	82/17632	0.02	4088 2932
Network	Network	GO:0001077	proximal promoter DNA-binding transcription activator activity, RNA polymerase II-specific	3/20	298/17632	0.022	3725 6774 3661
Network	Network	GO:0008565	protein transporter activity	2/20	88/17632	0.022	3837 4170
Network	Network	GO:0047485	protein N-terminus binding	2/20	107/17632	0.03	7040 142
Network	Network	GO:0042826	histone deacetylase binding	2/20	112/17632	0.032	5245 142
Network	Network	GO:0031490	chromatin DNA binding	2/20	119/17632	0.035	6774 4088
Network	Network	GO:0002020	protease binding	2/20	125/17632	0.037	596 2932
Network	Network	GO:0043021	ribonucleoprotein complex binding	2/20	128/17632	0.037	5499 4869
Network	Network	GO:0035326	enhancer binding	2/20	133/17632	0.039	3725 4088
Network	Network	GO:0005072	transforming growth factor beta receptor, cytoplasmic mediator activity	1/20	10/17632	0.042	4088
Network	Network	GO:0030957	Tat protein binding	1/20	10/17632	0.042	4869
Network	Network	GO:0034713	type I transforming growth factor beta receptor binding	1/20	11/17632	0.045	7040
Network	Network	GO:0000982	transcription factor activity, RNA polymerase II proximal promoter sequence-specific DNA binding	3/20	447/17632	0.045	3725 6774 3661
Network	Network	GO:0034452	dynactin binding	1/20	12/17632	0.045	2932
Network	Network	GO:0070410	co-SMAD binding	1/20	12/17632	0.045	4088
Network	Network	GO:0043023	ribosomal large subunit binding	1/20	13/17632	0.047	4869

Cluster	group	ID	Description	GeneRatio	BgRatio	p.adjust	geneID
Network	Network	GO:0001846	opsonin binding	1/20	14/17632	0.048	5245
Network	Network	GO:0035497	cAMP response element binding	1/20	14/17632	0.048	3725

2.2 KEGG

Cluster	group	ID	Description	GeneRatio	BgRatio	p.adjust	geneID
FullSet	FullSet	hsa03013	RNA transport	8/49	152/5894	0.002	7514 3646 8668 8665 26986 8761 1915 7329
Network	Network	hsa05200	Pathways in cancer	9/14	327/5894	0	6776 3725 6774 7040 4088 598 596 6502 2932
Network	Network	hsa05210	Colorectal cancer	5/14	62/5894	0	3725 7040 4088 596 2932
Network	Network	hsa05212	Pancreatic cancer	4/14	70/5894	0	6774 7040 4088 598
Network	Network	hsa05220	Chronic myeloid leukemia	4/14	73/5894	0	6776 7040 4088 598
Network	Network	hsa04110	Cell cycle	4/14	128/5894	0.002	7040 4088 6502 2932
Network	Network	hsa05145	Toxoplasmosis	4/14	133/5894	0.002	6774 7040 598 596
Network	Network	hsa05222	Small cell lung cancer	3/14	85/5894	0.006	598 596 6502
Network	Network	hsa04510	Focal adhesion	4/14	200/5894	0.006	3725 5499 596 2932
Network	Network	hsa04012	ErbB signaling pathway	3/14	87/5894	0.006	6776 3725 2932
Network	Network	hsa05142	Chagas disease (American trypanosomiasis)	3/14	104/5894	0.009	3725 7040 4088
Network	Network	hsa04722	Neurotrophin signaling pathway	3/14	127/5894	0.015	3725 596 2932
Network	Network	hsa05160	Hepatitis C	3/14	135/5894	0.017	6774 3661 2932
Network	Network	hsa04310	Wnt signaling pathway	3/14	151/5894	0.021	3725 4088 2932
Network	Network	hsa04630	Jak-STAT signaling pathway	3/14	155/5894	0.021	6776 6774 598
Network	Network	hsa05014	Amyotrophic lateral sclerosis (ALS)	2/14	54/5894	0.026	598 596
Network	Network	hsa05221	Acute myeloid leukemia	2/14	58/5894	0.028	6776 6774

Cluster	group	ID	Description	GeneRatio	BgRatio	p.adjust	geneID
Network	Network	hsa04062	Chemokine signaling pathway	3/14	189/5894	0.03	6774 2931 2932
Network	Network	hsa05211	Renal cell carcinoma	2/14	70/5894	0.036	3725 7040
Network	Network	hsa05140	Leishmaniasis	2/14	73/5894	0.037	3725 7040
Network	Network	hsa04662	B cell receptor signaling pathway	2/14	75/5894	0.037	3725 2932
Network	Network	hsa04350	TGF-beta signaling pathway	2/14	85/5894	0.044	7040 4088
Network	Network	hsa04210	Apoptosis	2/14	89/5894	0.044	598 596
Network	Network	hsa05215	Prostate cancer	2/14	89/5894	0.044	596 2932
Network	Network	hsa05323	Rheumatoid arthritis	2/14	92/5894	0.045	3725 7040

2.3 GO LOCATION

Cluster	group	ID	Description	GeneRatio	geneID
FullSet	FullSet	GO:0016020	membrane	75/99	23256 5701 10652 6811 91452 92521 7514 4539 4513 3178 59272 339390 30835 10332 3838 6441 5478 599 597 3840 8575 928 7113 3551 290 1803 10492 5725 634 302 3181 3329 9276 23521 3646 4836 128866 8665 8665 8729 3832 5707 51429 4841 26986 6143 4691 1653 408050 408050 2280 4140 9146 7917 7917 7917 7917 7917 7917 7917 819 25978 116841 81926 4705 4705 79001 81887 6224 10540 1655 1915 857 6449 9550
FullSet	FullSet	GO:0005576	extracellular region	53/99	5701 11344 10875 3178 59272 30835 10332 6441 5478 928 7113 290 1803 5725 634 302 3181 3337 3329 3313 3646 8668 128866 5707 51429 26986 4691 4140 9146 7917 7917 7917 7917 7917 7917 7917 23564 23564 23564 23564 23564 23564 23564 25978 7177 710 5683 55506 6224 10540 1655 1915 9550
FullSet	FullSet	GO:0005623	cell	113/99	689 23256 5701 11344 10652 6811 51187 91452 92521 10875 7514 4539 4513 3178 59272 339390 30835 10332 3838 6441 5478 599 597 3840 8575 928 7113 3551 290 85437 1803 10492 5725 634 302 3181 220988 50 3337 3329 3313 9276 23521 3646 8668 4836 128866 8665 8665 8729 6241 3832 5707 6729 84955 51429 4841 26986 8761 10146 9908 6143 4691 1653 23429 23429 408050 408050 2280 9360 4140 10465 11123 9146 7917 7917 7917 7917 7917 7917 7917 23564 23564 23564 23564 23564 23564 23564 819 25978 116841 23609 710 5683 81926 5204 57409 4705 4705 79001 81887 55506 6224 11200 7013 10540 1655 119710 1915 857 7329 6449 9550
FullSet	FullSet	GO:0009295	nucleoid	1/99	3313
FullSet	FullSet	GO:0030054	cell junction	12/99	5478 928 1803 634 302 3313 23521 26986 10146 6143 81926 857
FullSet	FullSet	GO:0031974	membrane-enclosed lumen	53/99	5701 51187 91452 10875 7514 3178 3838 5478 3840 8575 85437 10492 5725 302 3181 220988 50 3337 3329 3313 23521 3646 8729 5707 6729 84955 4841 4691 1653 23429 23429 9360 10465 7917 7917 7917 7917 7917 7917 7917 710 5683 57409 4705 4705 81887 55506 6224 11200 7013 1655 1915 7329

Cluster	group	ID	Description	GeneRatio	geneID
FullSet	FullSet	GO:0032991	protein-containing complex	70/99	5701 10652 6811 51187 92521 10875 7514 4539 4513 3178 6441 5478 599 8575 3551 85437 10492 634 302 3181 220988 3329 9276 23521 3646 8668 128866 8665 8665 3832 5707 6729 4841 26986 8761 10146 9908 6143 4691 1653 23429 23429 2280 10465 9146 7917 7917 7917 7917 7917 7917 7917 25978 116841 7681 5683 5204 4705 4705 81887 55506 6224 7013 10540 1655 1915 857 7329 6449 9550
FullSet	FullSet	GO:0043226	organelle	104/99	689 23256 5701 11344 10652 6811 51187 91452 92521 10875 7514 4539 4513 3178 59272 3838 6441 5478 599 597 3840 8575 928 7113 3551 290 85437 1803 10492 5725 634 302 3181 220988 50 3337 3329 3313 9276 23521 3646 8668 4836 128866 8729 3832 5707 6729 84955 51429 4841 26986 8761 10146 6143 4691 1653 23429 23429 408050 408050 2280 9360 4140 10465 9146 7917 7917 7917 7917 7917 7917 7917 23564 23564 23564 23564 23564 23564 23564 819 25978 116841 710 5683 81926 5204 57409 4705 4705 79001 81887 55506 6224 11200 7013 10540 1655 119710 1915 857 7329 6449 9550
FullSet	FullSet	GO:0044215	other organism	3/99	7514 30835 10332
FullSet	FullSet	GO:0044217	other organism part	3/99	7514 30835 10332
FullSet	FullSet	GO:0044421	extracellular region part	49/99	11344 10875 3178 59272 6441 5478 928 7113 290 1803 5725 634 302 3181 3337 3329 3313 3646 8668 128866 51429 26986 4691 4140 9146 7917 7917 7917 7917 7917 7917 7917 23564 23564 23564 23564 23564 23564 23564 25978 7177 710 5683 55506 6224 10540 1655 1915 9550
FullSet	FullSet	GO:0044422	organelle part	92/99	23256 5701 11344 10652 6811 51187 91452 92521 10875 7514 4539 4513 3178 3838 6441 5478 599 597 3840 8575 928 290 85437 1803 10492 5725 634 302 3181 220988 50 3337 3329 3313 9276 23521 3646 128866 8729 3832 5707 6729 84955 51429 4841 26986 6143 4691 1653 23429 23429 408050 408050 2280 9360 10465 9146 7917 7917 7917 7917 7917 7917 7917 23564 23564 23564 23564 23564 23564 23564 25978 116841 710 5683 81926 57409 4705 4705 79001 81887 55506 6224 11200 7013 10540 1655 1915 857 7329 6449 9550
FullSet	FullSet	GO:0044425	membrane part	39/99	23256 10652 6811 91452 7514 4539 4513 59272 339390 30835 10332 3838 6441 928 7113 3551 290 1803 634 302 3329 9276 4836 128866 51429 408050 408050 2280 819 25978 116841 81926 4705 4705 79001 1915 857 6449 9550
FullSet	FullSet	GO:0044456	synapse part	8/99	5701 3838 220988 3337 25978 116841 81926 6449
FullSet	FullSet	GO:0044464	cell part	113/99	689 23256 5701 11344 10652 6811 51187 91452 92521 10875 7514 4539 4513 3178 59272 339390 30835 10332 3838 6441 5478 599 597 3840 8575 928 7113 3551 290 85437 1803 10492 5725 634 302 3181 220988 50 3337 3329 3313 9276 23521 3646 8668 4836 128866 8665 8665 8729 6241 3832 5707 6729 84955 51429 4841 26986 8761 10146 9908 6143 4691 1653 23429 23429 408050 408050 2280 9360 4140 10465 11123 9146 7917 7917 7917 7917 7917 7917 7917 23564 23564 23564 23564 23564 23564 23564 819 25978 116841 23609 710 5683 81926 5204 57409 4705 4705 79001 81887 55506 6224 11200 7013 10540 1655 119710 1915 857 7329 6449 9550
FullSet	FullSet	GO:0045202	synapse	11/99	5701 3838 220988 3337 6143 25978 116841 81926 6224 7329 6449
FullSet	FullSet	GO:0099080	supramolecular complex	5/99	11344 92521 3832 2280 10540
Network	Network	GO:0016020	membrane	12/20	3837 5245 6774 5499 7040 4088 598 4170 596 2932 4869 142
Network	Network	GO:0005576	extracellular region	4/20	3837 5245 5499 7040

Cluster	group	ID	Description	GeneRatio	geneID
Network	Network	GO:0005623	cell	20/20	7942 3837 6776 5245 3725 6774 5499 7040 4088 22809 3661 598 4170 596 6502 4666 2931 2932 4869 142
Network	Network	GO:0030054	cell junction	3/20	5499 598 4869
Network	Network	GO:0031974	membrane-enclosed lumen	17/20	3837 6776 5245 3725 6774 5499 7040 4088 22809 3661 598 4170 596 6502 2932 4869 142
Network	Network	GO:0032991	protein-containing complex	15/20	3837 3725 6774 5499 4088 22809 598 4170 596 6502 4666 2931 2932 4869 142
Network	Network	GO:0043226	organelle	20/20	7942 3837 6776 5245 3725 6774 5499 7040 4088 22809 3661 598 4170 596 6502 4666 2931 2932 4869 142
Network	Network	GO:0044215	other organism	1/20	3837
Network	Network	GO:0044217	other organism part	1/20	3837
Network	Network	GO:0044421	extracellular region part	4/20	3837 5245 5499 7040
Network	Network	GO:0044422	organelle part	17/20	3837 6776 5245 3725 6774 5499 7040 4088 22809 3661 598 4170 596 6502 2932 4869 142
Network	Network	GO:0044425	membrane part	4/20	5245 598 4170 596
Network	Network	GO:0044456	synapse part	6/20	5245 6774 5499 598 2931 2932
Network	Network	GO:0044464	cell part	20/20	7942 3837 6776 5245 3725 6774 5499 7040 4088 22809 3661 598 4170 596 6502 4666 2931 2932 4869 142
Network	Network	GO:0045202	synapse	6/20	5245 6774 5499 598 2931 2932
