# Supplementary material for: IL6-mediated HCoV-host interactome regulatory network and GO/Pathway enrichment analysis
Source: PLoS Comput Biol. 2020 Sep 30;16(9):e1008238. doi: 10.1371/journal.pcbi.1008238 (PMC7561109; doi:10.1371/journal.pcbi.1008238)
Supplement: S1 Instruction — (PDF) [file pcbi.1008238.s005.pdf]

Instructions to access theRING Database and reproduce the paper results

1) Go to https://precious.polito.it/theringdb/
2) Click on the access link.
3) In the Dashboard, click on the Menu icon (top-left) and then on "Graph Tools"

Cluster network

4) Verify that the Filter panel has the following buttons green: Validated, Manually curated, Directional, Gene, TF. The other ones have to be white (disabled)
5) Click on "Entity Names"
6) Copy and Paste the following list of genes into the input box and then click Search (might take several minutes).

BTF3, SCFD1, PSMC2, TFEB, TWF2, YKT6, KPNB1, STX5, STAT5A, RSL24D1, ACBD5, PHB, JUN, STAT3, PPP1CA, SPECC1, FGL2, XPO1, TGFB1, FGL2, SMAD3, BTF3, ATF5, ND4L, COX2, HNRNPA1, ACE2, CLEC4G, CD209, CLEC4M, IRF3, KPNA2, SFTPD, PPIA, BCL2L1, BCL2L2, MCL1, BCL2A1, BCL2, SKP2, KPNA4, PRKRA, CD9, TMPRSS2, IKBKB, ANPEP, ACE2, ZCRB1, DPP4, HNRNPA1, SYNCRIP, PTBP1, CEACAM1, ZCRB1, ACE2, ANXA2, HNRNPA2B1, HNRNPA3, ACO2, DNAJB1, HSPD1, HSPA9, COPB2, RPL13A, EIF3E, EIF3I, NMT1, CHMP4B, EIF3F, GBF1, RRM2, KIF11, PSMD1, SRP54, NUDCD1, NACA, SNX9, NONO, GSK3A, GSK3B, PABPC1, PABPC4, HNRNPA1, HNRNPA2B1, NPM1, G3BP1, G3BP2, RPL19, PARP1, NCL, DDX1, RYBP, PPIA, NOMO3, FKBP1A, PPIG, MARK3, PPIH, RCAN3, HGS, BAG6, DDAH2, CAMLG, CHMP2B, SNAP47, MKRN2, TPSAB1, SERPING1, MKRN3, PSMA2, ABHD17A, PFDN5, MIF4GD, NDUFA10, VKORC1, LAS1L, H2AFY2, RPS20, CHEK2, TERF1, DCTN2, DDX5, C11orf74, EIF3F, EEF1A1, CAV1, IKBKB, UBE2I, SGTA, ATP6V1G1

7) Layout the resulting network with Prefuse Force layout.
8) Manually select (keeping the Shift button pressed) all disconnected nodes.
9) Click on the DELETE Icon to remove them from the network

MiRNA enrichment

10) Select all remaining nodes by clicking the Layer 0 button
11) Click on the Expand-entity 1-level GO! Button.
12) In the pop-up panel select only MIRNA, MIRIAD (intronic) and MIRIAD (intragenic)
13) Select the "ALL INTERACTORS and ALL their CONNECTIONS option"
14) Click on the "Expand Node" button
15) This operation creates 3 miRNA nodes and attaches them to their hosts.
16) Layout the network again

IL6

17) Click on Utils: "ADD new entity(s)" and write IL6 in the textbox.
18) Click on GO! To add the node.
19) In the Advanced Filters section, select the Validated, and make sure the following DBs are selected: TRRUST, PSCAN, TCOF, SIGNOR, MINT, IrefIndex
20) Click on the "GO!" button
21) This operation attaches IL6 to the cluster.
22) Layout the network again

DRUGS enrichment

23) Select all nodes by clicking the Layer 0 button
24) Click on the Expand-entity 1-level GO! Button.
25) In the pop-up panel select only Manually curated, DGIdb, DRUGBANK pathways, and DRUGBANK targets.
26) Select the “INTERACTORS with AT LEAST 2 CONNECTIONS to the network”
27) Click on the “Expand Node” button
28) Layout the network again (if necessary click on FreeHand to layout the nodes manually)

Further instructions on the use of theRING Database can be found in the Supplemental Material of the following paper:

Politano, G., Di Carlo, S. and Benso, A. '*One DB to rule them all' — the RING: a Regulatory INteraction Graph combining TFs, genes/proteins, SNPs, diseases and drugs.* Database (2018) Vol. 2019: article ID baz108; doi: 10.1093/database/baz108
