# Supplementary material for: IL6-mediated HCoV-host interactome regulatory network and GO/Pathway enrichment analysis
Source: PLoS Comput Biol. 2020 Sep 30;16(9):e1008238. doi: 10.1371/journal.pcbi.1008238 (PMC7561109; doi:10.1371/journal.pcbi.1008238)
Supplement: Attachment — Submitted filename: Responses to reviewers.doc [file pcbi.1008238.s007.doc]

Turin June 3^rd^, 2020

Answers to Reviewer's Comments

**Comments to the Authors:
Please note here if the review is uploaded as an attachment.**

Reviewer #1: In this paper, the authors performed some preliminary analysis on the HCoV-host interactome.

I am aware that some of the provided results may be useful for the community. As stated by the authors, the computed enrichment analysis may allow scientists to better and more reliably infer candidate pathways and GOs for further inspection.

However, the present paper does not provide a significant improvement in terms of methodologies, nor provides a significant outcome in terms of results. Rather, it may be considered as the result of few queries performed on existing online repositories.

The “few queries” are the result of a 2 years of development of a software integrating tens of available repositories about Regulatory Data, that, queried individually, would make our “simple” analysis extremely time consuming. Integrating public repositories is not as simple as it may look mainly due to different data format, naming standards, and data quality.

Therefore, although such results may deserve to be shown to the community, since the paper lacks both methodological originality and significance of results, I think that it cannot be published on PLOS Computational Biology in its current form.

Reviewer #2: This paper by politano and benso describes a computational approach to study the HCoV-host interactome. The purpose is to enable scientists with limited experience with compuational analyses to study the HCoV infections and potential drug repurposing/targeting. The paper elegantly shows how many of the results presented in a recent paper by Zhou et al can be achieved by non-programmers using extremely easy-to-use tools previously developed by the authors and adapted to this specific case. These types of workflows are really useful to speed up research in highly time sensitive cases such as the COVID-19 pandemic. The manuscript is well written, and with a few exceptions (listed below) the results are presented in a clear and concise manner.

*One major issue that must be resolved before publication can be considered: The link* <https://precious.polito.it/covid-19/> *doesn't work, so it's not really possible to evaluate the feature*

Done: The link has been counterchecked in the paper and it now works correctly.

Minor comments and suggestions:
It seems to me that the title promises a lot more than what is delivered in this paper. May I suggest a re-wording to more accurately reflect what I see as the ultimate value of this paper: the fact that it enables fast and easy reproduction of extremely advanced computational analyses by scientists with no explicit programming experience.

We changed the title as suggested by the reviewer

*The first paragraph of materials and methods feels like it belongs in the introduction as it concisely describes the scope and provides essential context for the rest of the work presented.*

Done: the paragraph has been moved at the end of the Introduction section

*Figure 1 and 2: Legends seem incomplete. What do the node and edge colors/shapes represent? I would also suggest to include the virus proteins in these graphs.*

Done: captions of both the figures have been improved by adding nodes/edges legend explanation.

*Figure 2: All of these drugs interact with human proteins, none with virus proteins. What do these drugs do to the proteins they target?*

*They* ***may not affect HCoV-host interaction at all (or they may even enhance it).*** *I realize that it's not within the scope of the paper to dive into the biology, but these results should be discussed a bit more to highlight the value of this feature.*

As a premise, there’s still no consensus about clinical effects of drugs administered in real patients after months of wait&watch treatments. Researchers are still debating about chloroquine capabilities, for instance, so we think the effect (direct/intermediate/long-term/..) of all those drugs cannot be easily discussed starting only from network analysis. We think that the presentation of the drugs list may be useful for researcher who already have previous knowledge on one or some of them. Based on this premise is our idea to highlight (among the other) their possible association to SARS_CoV pathological context; of course, their specific mechanism of action, especially in such a unprecedent scenario, should be confirmed with ad hoc experiments.

Detailed references to drugs-protein interactions are reported in the file Edges.csv (that contains the list of all the interaction identified in the cluster network, along with their database source and literature references, when available). It’s out to the scope of this paper to provide an accurate assessment about the way of interaction of any interacting drug. Given the high variability of their mechanism of action, the difficulty to provide a comprehensive discussion, and the impossibility to countercheck with a wet-lab assessment, we pointed out direct references the original databases that reported the interaction.

*Section 3.2. "three main branches" - they are the only three ontologies in the gene ontology*

Done, rephrased the text according to the suggestion. It now reads: “the three Gene Ontologies (GO [5]) (i.e., Molecular Function, Cellular Component and Biological Process);”

Reviewer #3: In the manuscript titled “A Systems Biology study of an IL6-mediated HCoV-host interactome: Drug Repurposing and GO/Pathway Enrichment analysis” by Politano and Benso, the authors describe a computational systems biology analysis of the HCoV-host interactome aimed at the identification of candidate drugs with anti-SARS-CoV-2 effects.

The main and critical point that should be addressed is to provide a clear and adequate rationale for this study, highlighting the differences with the study that it is based on (Zhou et al, 2020). The authors have re-analyzed the HCoV-host interactome described in Zhou et al, and performed a different type of drug repurposing analysis whose results shared only one candidate drug with the original study.
More specifically:

1) *Zhou et al generated their HCoV-host interactome based on validated published data. The authors of this novel manuscript found that 115 of the 135 proteins in the HCoV-host interactome showed no obvious interactions and focused their analysis on a set of 20 proteins forming a well-defined interactome. How are 115 proteins in the original HCoV-host interactome not connected? The authors should discuss their findings.*

Zhou et al. made an excellent and very useful work in identifying the HCoV-host interactome and provided an high level of statistical validation (GSEA) for grouping their findings, however we focused our attention on: “*[…] reliable interactions, […] most conservative settings, where all interactions are validated, manually curated, and limited to signaling or regulatory actions […]”* as we explained in the paper in section 3.1. This means we are not claiming that some protein appears statistically enriched in a given context, we instead provided for any edge of the network one or more literature references that provide a biological explanation of the interaction. Given this higher level of reliability, of course we only connected genes that currently have a biological established rationale behind their linkage, which of course results in a limited set of very reliable interactions. Which is also the complimentary role of our paper when compared against Zhou et al. work.

2) *The added value and improvement over the original analysis is not clear besides the addition of IL-6 (other cytokines might be considered as well).*

Added value is explained in the previous answer.

Regarding IL6 and other cytokines:

The choice of IL6 has been suggested by the fact that at the time of writing that specific interleukin was reported as the responsible for the excessive inflammatory response that result in higher respiratory distress. See Xiaohua Chen, Binghong Zhao, Yueming Qu, Yurou Chen, Jie Xiong, Yong Feng, Dong Men, Qianchuan Huang, Ying Liu, Bo Yang, Jinya Ding, Feng Li, *Detectable serum SARS-CoV-2 viral load (RNAaemia) is closely associated with drastically elevated interleukin 6 (IL-6) level in critically ill COVID-19 patients.* 2020.

Zhou et al performed network-based drug repurposing and gene-set enrichment analysis. The authors of this manuscript should describe what weaknesses in the original work they address here with their analysis and compare and discuss the findings of the two analyses, highlighting the added value and improved accuracy of their study.

See answer to point 1

*Two almost completely different sets of drugs generated from the same data may raise more questions than provide answers.*

Given the current level of uncertainty regarding COVID therapies (two months after sending this paper to Plos) it is in our opinion better to have a large set of hypothesis to test, than just few options due to a limited perspective. Our goal is to propagate knowledge, this knowledge is currently conserved in several repositories. We agree it is interesting that this strange behavior arises, but the required level of validation overcome our wet-lab unavailability.

*Thus, it is essential that the authors provide evidence that their analysis is, at the very least, significantly complementary to the original study.*

See answer to point 1

*3) What is the added value introduced by the integration of miRNA in the interaction map?*

miRna are known to mediate several regulatory mechnisms, but also to be powerful biomarkers for several diseases. The usefulness of highlighting miRNAs possibly involved in the Covid interactome seem absolutely obvious according to the goal of the paper: to share and propagate knowledge that may help other scientists involved in SARS-CoV-2 research to better design their studies or uncover new hypothesis.

Two minor points:
1) Overall, the manuscript reads well but it should be carefully reviewed for syntax and incomplete sentences, e.g. “The resulting network is presented in” on Page 3 is incomplete. Similarly, the sentence “Concluding that IL-6 induces Bcl-2 expression to perform cytoprotective functions in response to oxygen toxicity, and that this effect is mediated by alterations in the interactions between BAK and MFNS” is incomplete as well.

The text has been corrected.

*2) The link* <https://precious.polito.it/covid-19/> *is broken.*
The link has been counterchecked in the paper and it now works correctly.
